# A Convenient Strategy to Access Diverse Libraries of Amphiphilic Compounds Based on Glycopeptoids

**DOI:** 10.1002/chir.70121

**Published:** 2026-07-09

**Authors:** Ludovica Dei, Stefano Cadeddu, Claude Taillefumier, Chiara Migone, Angela Fabiano, Anna Maria Piras, Maria Chiara Santangelo, Sebastiano Di Pietro, Valeria Di Bussolo, Gaetano Angelici

**Affiliations:** ^1^ Dipartimento di Chimica e Chimica Industriale Università di Pisa Pisa Italy; ^2^ Université Clermont Auvergne, Clermont Auvergne INP, CNRS, ICCF Clermont‐Ferrand France; ^3^ Dipartimento di Farmacia Università di Pisa Pisa Italy

**Keywords:** amphiphilic molecules, glycopeptoids, micelles, peptoids, self‐assembly

## Abstract

A convergent synthetic approach has been developed to access a potentially wide library of amphiphilic glycopeptoids. The intrinsic modularity of apolar peptoid units, coupled with the ability to conjugate various two‐headed monosaccharide units, enables the exploration of a vast chemical space for the precise regulation of amphiphilic properties and the formation of diverse nanomaterials through self‐assembly. A full characterization of the physical and chemical properties of the self‐assembled materials obtained has been conducted on some representative molecular structures designed for the formation of aggregates. The comprehension of the relation between the molecular structure of this family of amphiphilic compounds and their self‐assembly could lead to the “on demand synthesis” of functional excipient for drug delivery or compounds for the stabilization and crystallization of membrane proteins.

## Graphical abstract

A synthetic platform based on a convergent synthetic strategy enables rapid access to a diverse amphiphilic glycopeptoids library. These modular systems exhibit self‐assembly into nanoscale aggregates, highlighting their potential as customizable platforms for drug delivery, biomimetic materials, and membrane protein stabilization.

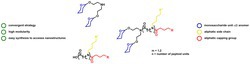

## 1 Introduction

**FIGURE 1:**
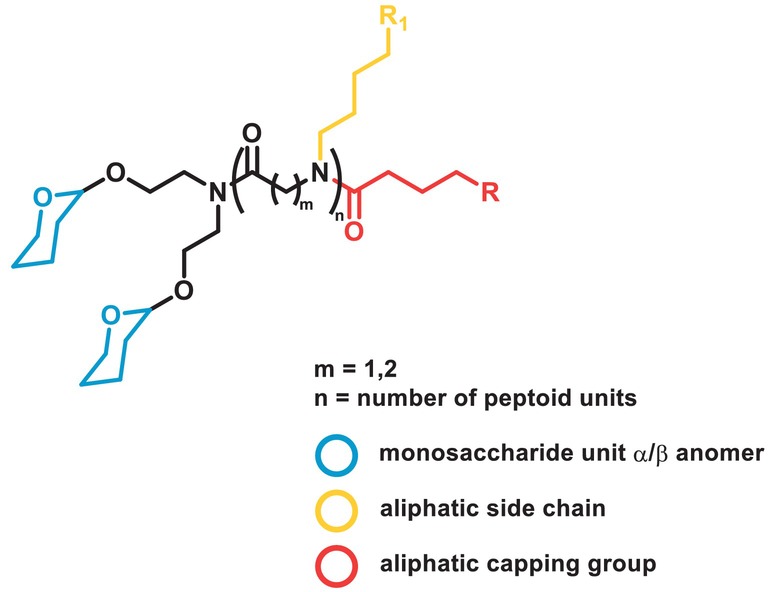
Representation of the possible variations applicable to this family of two‐headed glycopeptoids.

Amphiphilic compounds, as a result of their hydrophilic/hydrophobic dual nature, are able to self‐assemble on a large variety of structures, such as micelles, vesicles, nanotubes, nanofibers, and lamellae [1, 2, 3, 4, 5, 6, 7, 8]. Such versatility opens the way for a plethora of different applications in drug delivery [9, 10, 11, 12], emulsification [13, 14], surface modification [15, 16, 17, 18], biomembrane mimicry [19, 20, 21], surfactants [7], nanoparticle stabilization [22, 23, 24], and membrane protein crystallization [25, 26, 27, 28, 29]. Several chemical structures have been proposed in the last decades to access a large variety of amphiphile libraries and explore different nanostructures, like hemifluorinated detergents [30, 31, 32, 33], tripod detergents [34, 35, 36], cholesterol‐based molecules [37, 38, 39], amphiphilic polymers [40, 41, 42, 43], and peptide surfactants [44, 45, 46, 47]. However, despite past and current efforts, a pressing need for new amphiphiles remains. Among those possibilities, we were interested in the use of a particular family of compounds, with peculiar features, called glycopeptoids [48]. Peptoids are peptidomimetic oligomers composed of *N*‐substituted glycine units [49]. Due to their intrinsic modularity, excellent cell membrane permeability, wide variety of side chains, and easy synthesis, they represent an extremely promising choice to obtain suitable apolar tails [50, 51, 52]. Moreover, compared to peptides, peptoids have the advantage of reduced hydrogen bonding capacities due to their *N*‐substituted backbones, which reduces the energetic penalty for desolvating tertiary amides. Recent advances in the control of the conformational labile *cis*/*trans* isomerism of the peptoid backbone, depending on the nature of the sidechain, allow today for the synthesis of conformationally stable oligomers, eventually adding another level of control on their self‐assembly [53]. Carbohydrate derivatives are attractive candidates as polar head groups in amphiphilic molecules due to their biocompatibility, availability, inherent chirality, and depending on the chosen stereochemistry of the monosaccharide, the potential to enable targeted drug delivery strategies. Glycopeptoids have already been reported in the literature, dating back to the pioneering work of Roy et al., who synthesized peptoids bearing glycomoieties on the side chains of long oligomers [54]. However, these were primarily developed to mimic bioactive glycopeptides, rather than as amphiphilic systems. Different types of linkages have been used, such as *N*‐ [55, 56, 57], *O*‐ [58, 59, 60], *C*‐ [61, 62, 63], or *N*‐alkylaminooxy‐linkers [64, 65, 66]. Nevertheless, most of the designed glycoderivatives feature monosaccharides or linear di‐ and polysaccharides as polar heads, which limits the possible explorations of the geometrical parameters related to their shape and size [67]. In contrast, the design proposed in this work (see Figure 1) is based on the direct conjugation of a peptoid hydrophobic tail with a two‐headed carbohydrate‐based polar group, via a glycosidic bond. We hypothesized that a two‐headed polar group may more effectively satisfy the packing parameters required for micelle formation, similarly to what has been shown by Gellman with amphiphilic tripods [34], and by Durand with hybrid fluorinated and hydrogenated double‐chain surfactants [68].

Considering the various differentiating factors, including the easy accessibility of diverse apolar primary amines for peptoid oligomer synthesis, the modularity of peptoids, the possibility to incorporate *α*‐ or *β*‐peptoid units, the choice of different monosaccharides, and the control over anomeric isomerism through the glycosylation reaction, glycopeptoids offer a highly versatile platform for tuning the physical properties of micelles and other self‐assembled structures. Despite this potential, a major limitation of this approach has been the absence of an optimized and practical synthetic methodology. In this work, we report the development of an optimized synthesis, detailing the key steps that may present challenges when scaling up to larger libraries. As a case study, we describe the synthesis and self‐assembly behavior of a selected family of glycopeptoids featuring a two‐headed *β*‐d‐glucose‐based polar group and two hydrophobic tails: one derived from an aliphatic side chain of varying lengths attached to the peptoid nitrogen, and the other from an aliphatic capping group of adjustable length.

## 2 Materials and Methods

All reactions, unless otherwise specified, were run under a positive pressure of argon, with exclusion of moisture from reagents and glassware, transferring solvents and liquid reagents with hypodermic syringes, according to the Schlenk technique. Glassware has been dried with a heating gun under vacuum and allowed to cool under argon. Anhydrous solvents were used as purchased (glass bottles with Sure/Seal caps) or were dried over aluminum oxide via a solvent purification system (MBRAUN Solvent Purification System SPS‐5). The remaining commercial reagents, unless otherwise specified, were used as received. Et_3_N was freshly distilled in an argon atmosphere. TLC analyses were performed with Macherey‐Nagel 60 UV 254‐nm (0.2 mm) silica gel plates. LC‐MS‐DAD analyses of the intermediate compounds were performed on an Acquity UPLC Water instrument (Phase A: 95/5 H_2_O/ACN + 0.1% Formic Acid, Phase B: ACN + 0.1% Formic Acid; Acquity UPLC 2.1 × 100‐mm column, BEH C18, 1.7 μm; Flow 0.5 mL/min) coupled with an Acquity QDa Water mass spectrometer (Probe temperature: 600°C; ESI capillary voltage 1.5 kV; Cone voltage 15 V; Mass range 60–1000 Da) and a PDA el Detector (wavelength range 200–800 nm). Separation was achieved under the following chromatographic conditions: 0% B for 1.5 min then increased linearly to 100% B in 6.5 min and held for 1.5 min, then returned to 0% B over 1 min and re‐equilibrated for 2 min. The temperature of the chromatographic column was maintained at 40°C. Purifications by flash chromatography were performed using Merck 60 silica gel. ^1^H‐NMR and ^13^C‐NMR spectra were recorded at 400/500 MHz and 100/126 MHz, respectively, with JEOL 400‐MHz/500‐MHz spectrometers using the signal of the deuterated solvent used for analysis as a reference. Some ^1^H‐NMR and ^13^C‐NMR spectra were recorded at 400 and 100 MHz with a Bruker Avance III 400‐MHz spectrometer. The following multiplicity abbreviations are used: (s) singlet, (ls) large singlet, (d) doublet, (t) triplet, (q) quartet, (m) multiplet, and (br) broad. DOSY map was recorded with a Bruker Avance III 400‐MHz spectrometer. ESI‐HRMS spectra of the final and tested compounds were recorded by direct injection at flow rate 7 μL/min into an Orbitrap high‐resolution mass spectrometer (Thermo, San Jose, CA, USA) equipped with an H‐ESI source. The working conditions are as follows: negative polarity, atomization voltage −3.2 kV, capillary temperature 290°C, S‐lens RF level 50. Sheath and auxiliary gases were set to 28 and 4 (arbitrary units), respectively. Xcalibur 4.2 software (Thermo) was used for acquisition and analysis. A nominal resolution (at m/z 200) of 140,000 was used to acquire the spectra.

For biological evaluations: Dulbecco's modified Eagle's medium (DMEM), calf bovine serum (CBS), Dulbecco's Phosphate Buffer Saline (DPBS), penicillin, and streptomycin were purchased from Sigma‐Aldrich (Milan, Italy). Cell proliferation reagent (WST‐1) was provided by Roche diagnostic (Milan, Italy).

### 2.1 General Synthetic Procedures

#### 2.1.1 General Procedure A: SN_2_ of Bromides With *n*‐Nonylamine

To a solution of bromoacetic ester (1.2 equiv.) in anhydrous THF (0.2 M), cooled to 0°C, Et₃N (2.0 equiv.) was added. Subsequently, *N*‐nonylamine (1.0 equiv.) was added dropwise at 0°C, and the reaction mixture was stirred at room temperature. After completion of the reaction, as monitored by TLC, the mixture was filtered through cotton and the solvent was removed under reduced pressure. The crude residue was used directly in the next step without further purification or characterization.

#### 2.1.2 General Procedure B: Anhydride Preparation

To a solution of DCC (1.0 equiv.) in anhydrous CH_2_Cl_2_ (0.15 M), the acid (2.0 equiv.) was added, and the reaction mixture was stirred at room temperature for 1 h. After precipitation of Dicyclohexylurea (DCU), the mixture was filtered through Celite, and the filtrate was used directly in the next step.

#### 2.1.3 General Procedure C: Acylation of *N*‐Nonylglycine Benzyl Ester

To a solution of *N*‐nonylglycine benzyl ester (1.0 equiv.) in anhydrous EtOAc (0.14 M) was added Et₃N (1.5 equiv.). A solution of freshly prepared anhydride in CH_2_Cl_2_ (1.5 equiv.) was then added dropwise, and the reaction mixture was stirred at room temperature overnight. After completion of the reaction, as monitored by TLC, the mixture was successively washed with 1‐N HCl and saturated NaHCO₃ (aq). The combined organic layers were washed with brine, dried over Na_2_SO₄, and concentrated under reduced pressure. The crude residue was purified by flash chromatography on silica gel to afford the desired product.

#### 2.1.4 General Procedure D: Deprotection of Benzyl Esters via Hydrogenolysis

To a solution of the benzyl ester (1.0 equiv.) in MeOH (0.04 M) was added Pd/C (10% w/w). The reaction flask, under an argon atmosphere, was then filled with H_2_, and three vacuum/H_2_ cycles were performed. The reaction mixture was stirred under a H_2_‐saturated atmosphere at room temperature overnight. After completion of the reaction, as monitored by TLC, the mixture was filtered through Celite, and the solvent was removed under reduced pressure to afford the desired product.

#### 2.1.5 General Procedure E: Peptoid Coupling Reaction

To a solution of the peptoid (1.0 equiv.) in anhydrous CH_2_Cl_2_ (0.01 M) was added HATU (1.1 equiv.), and the mixture was stirred at room temperature for 20 min. Diethanolamine diglucosyl tetrabenzoylate (1.0 equiv.) and Et₃N (3.0 equiv.) were then added successively dropwise, and the reaction mixture was stirred at room temperature. After completion of the reaction, as monitored by TLC, the mixture was diluted with CH_2_Cl_2_ and washed successively with 1‐N HCl and saturated NaHCO₃ (aq). The combined organic layers were washed with brine, dried over Na_2_SO₄, and concentrated under reduced pressure. The crude residue was purified by flash chromatography on silica gel to afford the desired product.

#### 2.1.6 General Procedure F: Glycopeptoid Formation

To a solution of diglucosyl tetrabenzoate peptoid (1.0 equiv.) in MeOH (0.06 M) was added dropwise a freshly prepared solution of MeONa in MeOH (15.0 equiv., 0.39 M), and the reaction mixture was stirred at room temperature for 3 h. After completion of the reaction, as monitored by TLC, Amberlite IR‐120 resin (previously washed with MeOH) was added until neutral pH was reached and then removed by filtration. The filtrate was concentrated under reduced pressure, and the crude residue was purified by trituration with isopropyl ether to remove methyl benzoate, affording the desired product.

#### 2.1.7 Aggregation of Glycopeptoids in ater: Size Distribution, Critical Aggregation Concentration (CAC) and Zeta Potential Measurements

The size distribution of glycopeptoid aggregates in aqueous solution was obtained by dynamic light scattering (DLS) measurements with a Zetasizer Nano ZS nanoparticle analyzer (Malvern, detection angle = 173°) at 25°C. Samples were prepared using Milli‐Q water, and the resulting solutions, obtained with the help of ultrasound, were filtered through cellulose acetate (0.45 μm) filters. At least four separate measurements were made for each solution. The CAC of the glycopeptoid **22c** was determined by measuring the surface tension (Tensiometer du Noüy, Force Tensiometer K6 from KRÜSS (GmbH)) at 20°C of aqueous solutions of **22c** with decreasing concentrations starting from a stock solution ([**22c**]: 4.09 mM) prepared in Milli‐Q H_2_O. Milli‐Q H_2_O was used as a reference, with γ equal to 72.8 mN/m at 20°C [69]. Surface tension values were plotted versus logarithms of the **22c**'s concentrations, linear fits were applied to the data points, and CAC was determined as the intersection of the two linear fits relative to the initial plateau and the further surface tension increasing (*y = −0.6169x + 29.312 and y = −23.86x − 66.299*). The zeta potential value of the glycopeptoid **22c** aggregates in water (26.56 mM) was evaluated by using a Zetasizer Nano ZS (Malvern) at 25°C.

#### 2.1.8 Cell Culture Condition

Fibroblast cells BALB/3T3 clone A31 (CCL‐163) were purchased from American Type Culture Collection (USA). Cells were grown in a CO_2_ incubator at 37°C and 5% CO_2_ with cells subcultured at 70% confluency, in Dulbecco's Modified Eagle's Medium (DMEM), supplemented with 2 mM l‐glutamine and 1% penicillin/streptomycin and 10% calf bovine.

#### 2.1.9 Cell Viability

Fibroblast cells BALB/3T3 clone A31 were seeded in 96‐well culture plates at a concentration of 1 × 10^4^ per well for the analysis of cell viability at 4 h. Cells were incubated at 37°C and 5% CO_2_ and left to proliferate for 24 h prior to the incubation with **22c**. The culture medium from each well was removed and replaced with complete medium containing the sample diluted with complete DMEM at different concentrations in the range 0.079–0.79 mM. After 4 h of incubation, cell viability was assessed using WST‐1 tetrazolium salt reagent diluted to 1:10 and incubated for 4 h at 37°C and 5% CO_2_. Measurements of formazan dye absorbance were carried out at 450 nm, with the reference wavelength of 655 nm, using a microplate reader (BioTek 800/TS, Thermo Scientific).

## 3 Results and Discussion

**SCHEME 1:**
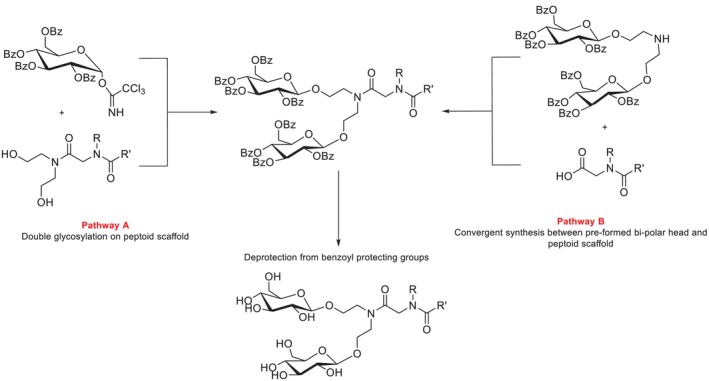
Tested synthetic strategies for the synthesis of two‐headed glucopeptoids.

The synthetic strategies explored to obtain these molecules are illustrated in Scheme 1. In Pathway A, the peptoid comprising two hydroxyethyl groups at its *C*‐terminal end is designed to act as a glycosyl acceptor. In contrast, Pathway B adopts a convergent approach, in which the presynthesized polar head and the apolar peptoid chain are joined via an amide bond through peptide coupling. In both cases, the connection between the polar head and the apolar tail happens at the late synthetic stage, allowing for a potential preparation of separated libraries of protected molecules, ready to be combined to explore their self‐assembly properties. However, to establish the most convenient pathway, it is necessary to optimize each reaction step. We have performed this initial optimization for Pathways A and B, choosing *β*‐d‐glucopyranose as the monosaccharide target, and as R and R′ a C9 and a C1 linear chains, respectively. Syntheses were performed in solution to allow possible scale up of the reactions.

### 3.1 Pathway A

#### 3.1.1 Apolar Peptoid Tail Synthesis

**SCHEME 2:**
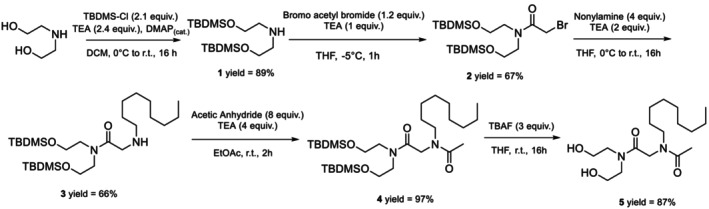
Synthesis of apolar tail following Pathway A.

Peptoid synthesis was carried out in solution using the classical submonomer approach, starting from diethanolamine protected at both hydroxyl groups with *tert*‐butyldimethylsilyl (TBDMS) groups **1**, as illustrated in Scheme 2. The synthesis proceeded with the acylation of the secondary amine using bromoacetyl bromide to afford compound **2**, which was subsequently reacted with nonylamine to yield compound **3**. This intermediate was then acylated with acetic anhydride to form compound **4**. Finally, compound **4** was deprotected by treatment with TBAF to remove the TBDMS groups, affording the desired glycosyl acceptor **5**. The reaction sequence was completed in good to excellent yields across all steps, without substantial issues, as shown in Scheme 2.

#### 3.1.2 Glycosylation of Peptoid Tail

**SCHEME 3:**
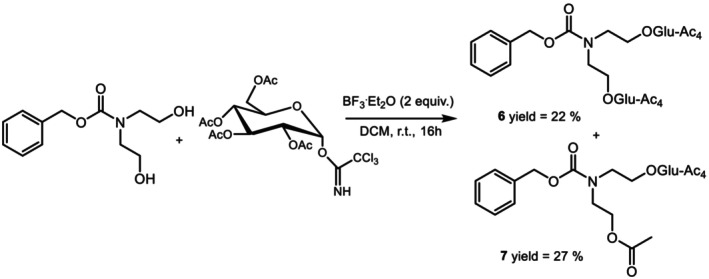
Double glycosylation reaction attempt using a peracetylated glucosyl trichloroacetimidate donor.

**SCHEME 4:**
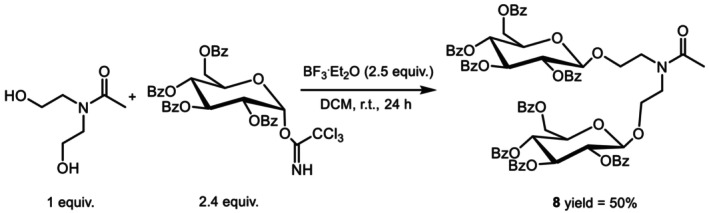
Double glycosylation test using perbenzoylated glucosyl donor.

We initially tested the double glycosylation reaction on model molecules using as glycosyl donor a freshly prepared trichloroacetamidate derivative of peracetylated glucose, as shown in Scheme 3. The choice of using the simple acetyl group, as hydroxyl protecting group, was made in the attempt to improve the atom economy of the synthesis. Moreover, these conditions constitute the most reliable approach to stereoselective glycosylations via neighboring group participation. The ester functionality promotes acetoxonium ion formation on the same face as the ester, enforcing nucleophilic attack of the glycosyl acceptor at the anomeric center from the less hindered face, thereby affording 1,2‐*trans*‐glycosides. Glucosyl‐type donors thus yield *β*‐linked products [70, 71, 72]. However, low yields of the double‐glycosylated compound **6** (22%) were obtained because of the concomitant formation of monosubstituted compound **7** (27%), as shown in Scheme 3. This is likely due to acetyl group migration onto the second free hydroxyl moiety, a known side reaction already reported in the literature [73]. Therefore, it was necessary to use the trichloroacetimidate derivative of d‐glucose protected by a more stable benzoyl group. The test glycosylation reaction towards the perbenzoylated *α*‐d‐glucopyranosyl trichloroacetimidate was tested on the very simple test molecule *N*,*N*‐bis(2‐hydroxyethyl)acetamide, as shown in Scheme 4. In this case, no migration of benzoyl was observed, obtaining exclusively product **8** with an isolated yield of 50%.

While the reaction could benefit from further optimization to improve the yield and reduce the amount of promoter required, the results were sufficient to proceed with the synthesis and evaluate Pathway A. Therefore, we applied the conditions used in Scheme 4 for the double glycosylation of compound **5**, using the trichloroacetimidate derivative of perbenzoylated d‐glucose.

Unfortunately, the same condition was not effective on **5**, leading to a poor yield of 5%.

**SCHEME 5:**
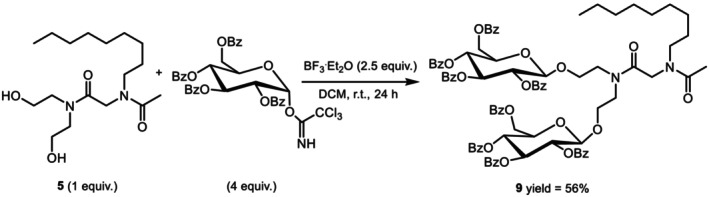
Double glycosylation of compound **5**.

A screening for the best condition for the glycosylation of **5** with the trichloroacetimidate derivative of perbenzoylated d‐glucose donor (Scheme 5) led to the use of an excess of glycosyl donor (4 eq) and BF_3_.OEt_2_ activator (2.5 eq), paying particular attention to maintain rigorous anhydrous condition. This protocol determined a 56% yield in the double‐glycosylated compound **9**.

The substrate‐dependent glycosylation process underlined the limit of Pathway A that might have required for each different peptoid a specific optimization of the reaction conditions. Such a strategy would not be compatible with the development of a straightforward synthetic platform to access diverse molecular structures and, subsequentially, different assembled materials. Therefore, we decided to explore the convergent strategy of Pathway B.

### 3.2 Pathway B

#### 3.2.1 Apolar Peptoid Tail Synthesis

**SCHEME 6:**
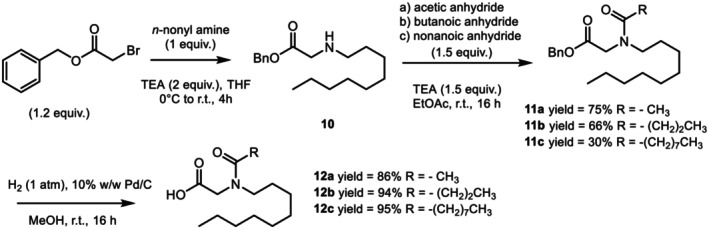
Synthesis of apolar tail moieties **12a**–**12c** through Pathway B.

Peptoid chain was synthesized in solution using the classical submonomer approach, starting from benzyl bromoacetate and *N*‐nonylamine obtaining intermediate **10**, which was afterwards filtered and, without further purifications, acetylated with aliphatic symmetric anhydride of different lengths, as shown in Scheme 6, obtaining good yields over two steps for **11a** and **11b**. The low yield of 30% obtained for **11c** is probably due to the more sterically hindered nonanoic anhydride. The deprotection of the benzyl ester, obtained through Palladium catalyzed hydrogenation reaction, proceeds in excellent yields affording **12a**–**12c**.

**SCHEME 7:**
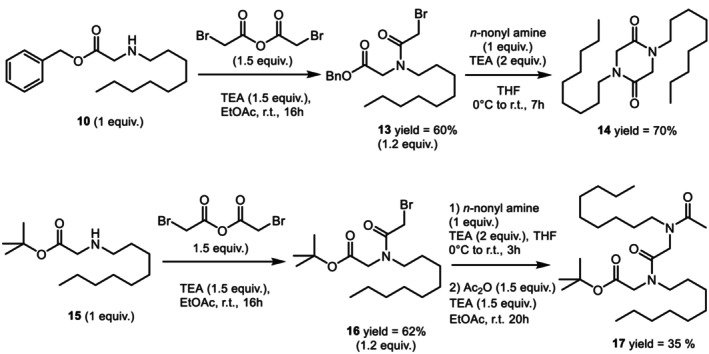
Diketopiperazine **14** formation and synthesis of di‐peptoid **17**.

It is worth mentioning some common precautions generally known in the synthesis of peptoids, which however are often not explicitly stated in the literature, as most peptoid oligomers are typically synthesized on solid phase amide resins. For solution phase synthesis, the choice of the ester as protecting group of the *C*‐terminus of the peptoid is essential to avoid diketopiperazine formation. In our case, the choice of benzyl ester as protecting group is advantageous because of the easy cleavage reaction through catalytic hydrogenation and because we initially limited our exploration adding a second apolar tail as a capping group. However, if we want to explore more complex geometries elongating the number of peptoid units it is necessary to use a *tert*‐butyl ester as protecting group, which avoid the formation of diketopiperazine as shown in Scheme 7. The benzyl ester intermediate **13** would indeed form efficiently the diketopiperazine **14**, if exposed to the elongation reaction of the peptoid, in solution. On the other hand, the use of *tert*‐Butyl ester intermediate **16** allows the formation of di‐peptoid **17**, in solution.

#### 3.2.2 Synthesis of the Two‐Headed Polar Group

**SCHEME 8:**
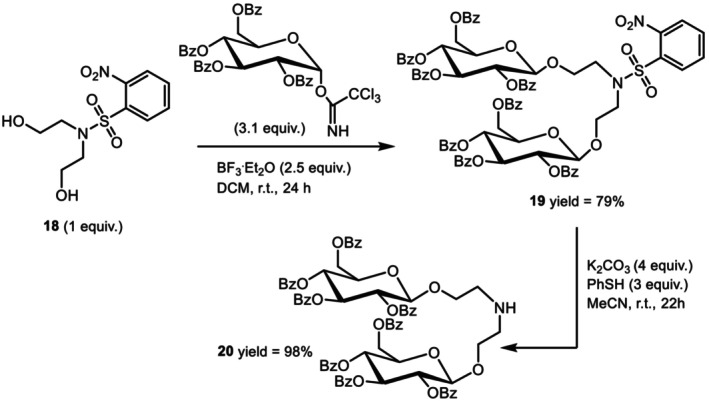
Double glycosylation of *N*‐nosyl protected diethanolamine.

For the synthesis of the two‐headed polar group, it is necessary to selectively protect the amine group of diethanolamine with an orthogonal protecting group towards the benzoyl ester, which protects the hydroxyl moieties of glucose. Therefore, a sulfonamide protecting group, such as the nosyl (Ns) group, was preferred, as it can be cleaved under very mild conditions, as illustrated in Scheme 8, in excellent yield [74]. Alternatively, a phenylsulfonamide protecting group can be used. However, although the final deprotection step using magnesium and ultrasound is effective, it results in the formation of *magnesium milk*, which complicates filtration and makes the process inefficient. In contrast, the nosyl group offers a more practical solution due to its ease of removal under mild conditions.

The glycosylation step was performed on compound **18** using an excess of the glycosyl donor (3.1 equiv.) and activator (2.5 equiv.) leading to a 79% yield of the bis‐glycosylated product **19**, as depicted in Scheme 8. The deprotection of the nosyl group led to the formation of compound **20** in excellent yield.

**SCHEME 9:**
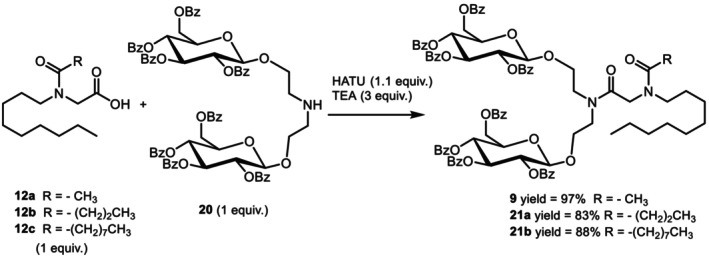
Coupling reaction between the synthesized apolar tails **12a**–**12c** and the free secondary amine **20**.

The key converging step involves the coupling of the free carboxylic acids **12a**–**12c** with the free secondary amine **20**, to obtain the products **9, 21a**, **and** −**21b**, respectively. This reaction was carried out under standard peptide coupling conditions using HATU as the coupling reagent and proceeded with excellent yields, as shown in Scheme 9.

**SCHEME 10:**
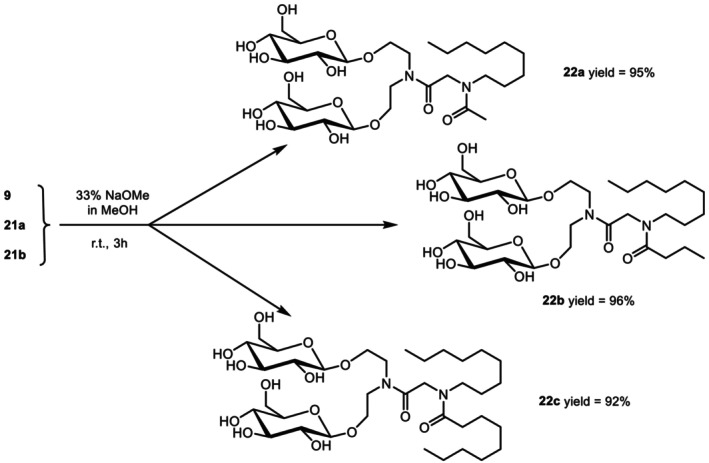
Deprotection of the glucose moieties from benzoyl protecting groups.

The last step is the deprotection of the benzoyl groups from the d‐glucose moieties of **9, 21a**, **and** −**21b**, using sodium methoxide in methanol in very mild conditions and obtaining the final amphiphilic molecules **22a**–**22c** in excellent yields as shown in Scheme 10.

Considering that Pathway B, unlike Pathway A, proceeds with consistently high yields at each step; that both the polar head and the apolar tail can be synthesized separately on a multigram scale and stored as stock compounds; and that the convergent coupling step proceeds with very good yields, it can be concluded that Pathway B is the more practical and efficient route. This paves the way for the synthesis of a broader and more complex library of amphiphilic molecules.

### 3.3 Self‐Assembly Studies

#### 3.3.1 Dimensional and Zeta Potential Analysis

Glycopeptoids' aggregation was studied by measuring the CAC and the size distribution of formed aggregates in water solution [75]. Since no studies about the self‐assembly properties of amphiphilic glycopeptoids are reported, the GNGs' (Glucose‐Neopentyl Glycol) CAC values synthesized by Gellman's group were used as references [76]. Among those, the compound GNG‐5 was mainly considered because it is the most similar to the proposed glycopeptoids. This molecule, indeed, has an amphiphilic structure that bears a polar head consisting of two glucopyranoside units arranged in parallel and a long‐chain double amide‐containing tail, structural features also found in **22a**–**22c** [77].

**FIGURE 2:**
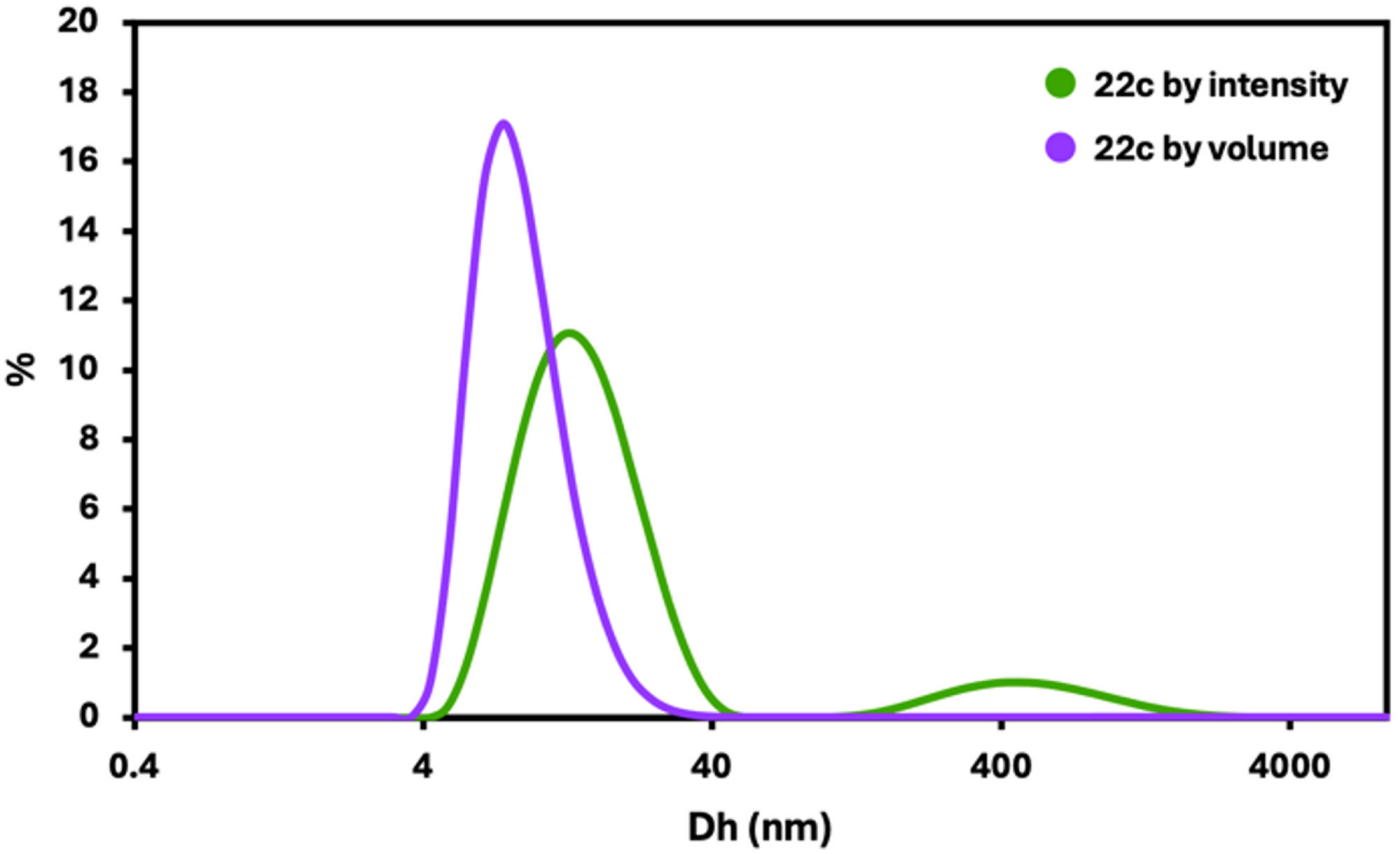
Representative diameter distribution of glycopeptoids aggregates. Overlay of intensity distribution and volume distribution of **22c**, 26.56 mM in water at 25°C.

Notably, as the author depicts, GNG‐5 showed a weak ability to self‐assemble, probably due to the low degree of hydrophobicity of the apolar tail, bearing two secondary amide functionalities and thus hydrogen bond donors. The obtained **22a**–**22c** glycopeptoids also bear two amide functionalities, but being tertiary, they should confer a higher degree of lipophilicity to the amphiphilic structure. On this basis, stock solutions in Milli‐Q water were prepared at concentrations reasonably higher than the Critical Micellar Concentration (CMC) (18 mM) reported for GNG‐5. Specifically, the stock solutions had concentrations of 57.66, 51.07, and 26.56 mM, for **22a**–**22c**, respectively. Successive dilutions were performed starting from the chosen concentrations, and the diameter distribution was analyzed at 25°C (Table 1) through DLS measurements. All samples appeared transparent, without turbidity or opalescence. All analyzed samples revealed the presence of aggregates. The analyses were performed until the scattering was no more sufficient to detect macromolecular assembly in solution. In the case of **22c**, very low concentrations were still sufficient to provide a good quality analysis, anticipating a low CAC value. In all cases, irrespective of the glycopeptoid type, a bimodal intensity‐based size distribution was observed. However, when considering the volume‐based size distribution, the presence of larger aggregates (*Dh = 100–300 nm*) was negligible compared to the main peak, which accounts for at least 98% of the total volume (Figure 2). The secondary population therefore represents only a minor fraction of the detected species. As typically observed for amphiphilic nanoassemblies, intensity‐based DLS distributions tend to overrepresent rare larger particles because of the *r* ^6^ dependence of light scattering, whereas, in this case, volume‐based distributions more accurately reflect the dominant population.

**TABLE 1:** Glycopeptoid aggregates in water: hydrodynamic diameters (Dh) of the principal aggregate population of **22a**–**22c** in aqueous solutions, at different concentrations, in H_2_O at 25°C and Polydispersity Index (PDI).

	Concentration	D_h_	
Glycopeptoid	(mM)	(nm ± SD)	PDI
**22a**	57.66	4.1 ± 0.1	0.08
	51.90	4.1 ± 0.1	0.09
	46.12	3.7 ± 0.1	0.08
	39.21	3.3 ± 0.2	0.08
	33.33	3.2 ± 0.2	0.06
**22b**	51.07	4.2 ± 0.1	0.07
	43.73	4.3 ± 0.2	0.06
	29.67	4.0 ± 0.1	0.08
	14.40	3.1 ± 0.3	0.13
**22c**	26.56	9.1 ± 0.2	0.16
	13.28	8.2 ± 0.3	0.14
	3.64	6.7 ± 0.2	0.09
	1.63	5.7 ± 0.1	0.07
	0.92	5.7 ± 0.2	0.06
	0.65	5.6 ± 0.1	0.06
	0.53	5.3 ± 0.1	0.05
	0.40	5.4 ± 0.1	0.04
	0.23	5.1 ± 0.3	0.08

The main peak for all glycopeptoids is in the range 3–9 nm, as reported in Table 1.

For each glycopeptoid, a trend indicating that the average diameter of the aggregates increases with compound concentration is observed. All hydrodynamic diameters are reported with one decimal place, in accordance with instrument resolution and established DLS best practices.

However, the difference in diameter between the lowest and highest concentrations analyzed is not statistically significant within each glycopeptoid series, except for **22c**, where the D_h_ of the aggregates at the highest concentration is approximately twice that at the lowest detectable concentration.

When looking at the size of the aggregates obtained at the lowest detectable concentration, an increase in average aggregate diameter is observed with increasing aliphatic glycopeptoid chain length (**22c** > **22b** ≥ **22a**).

As representative sample, **22c** at 26.56 mM underwent to zeta potential value determination. In water solution, the zeta potential resulted −5.03 ± 3.50 mV. Due to the absence of ionic functional moieties, the zeta potential value in water is close to neutrality. As expected, the value has a negative charge thanks to the contribution of the hydrophobic nature and the neutral glucopyranose rings, polarizing the interaction with water giving a typical slightly negative zeta potential [78, 79].

#### 3.3.2 Determination of the CAC

**FIGURE 3:**
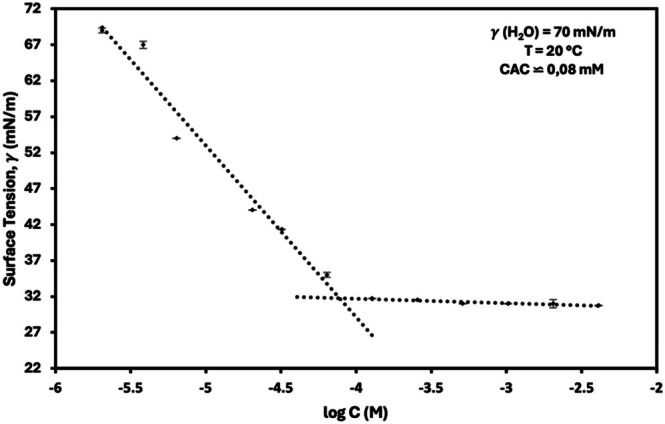
Determination of the critical aggregation concentration (CAC) of the glycopeptoid **22c** via surface tension.

As previously evinced, glycopeptoid **22c** showed a stronger ability to self‐assemble than **22a** and **22b**, providing aggregates up to concentrations in the order of 0.23 mM because, with equal polar head, the relative apolar tail is longer. For this reason, it was selected for the subsequent characterizations. CAC was evaluated by monitoring the surface tension values of the **22c**'s aqueous solutions at different concentrations. Decreasing the **22c** concentration caused a near‐constant trend in the surface tension value for a given range of concentrations ([**22c**] = 0.13 mM—4.09 mM). After a threshold, corresponding to CAC, a gradual increase in the surface tension value was observed as the concentration decreased until the typical surface tension value of water at *T* = 20°C was reached (70 mN/m). The collected data were plotted against the concentrations of the prepared samples (Figure 3).

The CAC of the **22c** derivative, which corresponds to 80 μM, is significantly lower than the CMCs of GNGs [77], indicating a stronger self‐assembly ability. This may result from its higher hydrophobic–hydrophilic ratio, due to longer alkyl chains and two tertiary amides, which, unlike the secondary amides in GNG‐5, aren't hydrogen bond donors. The low CAC value determined for **22c** suggests for a micellar‐like aggregation [75].

#### 3.3.3 DOSY Experiments

For a DLS technique‐independent valuation of the aggregates size formed by glycopeptoid **22c** in aqueous solution, a diffusion coefficient measurement was performed in D_2_O above the CAC using the NMR DOSY sequence. The sample was prepared considering the CAC estimated through surface tension measurements. The ^1^H NMR spectrum is characterized by a significant line broadening for the entire molecule's signal set, indicating a system that reorients more slowly due to the sizes of the aggregates. In this specific case, considering the sizes of these aggregates, the optimal set of parameters for the DOSY experiment (*δ* and *Δ*) did not allow for the measurement of a valid diffusion coefficient for D_2_O, given the substantial difference in the diffusion properties of the two systems.

**FIGURE 4:**
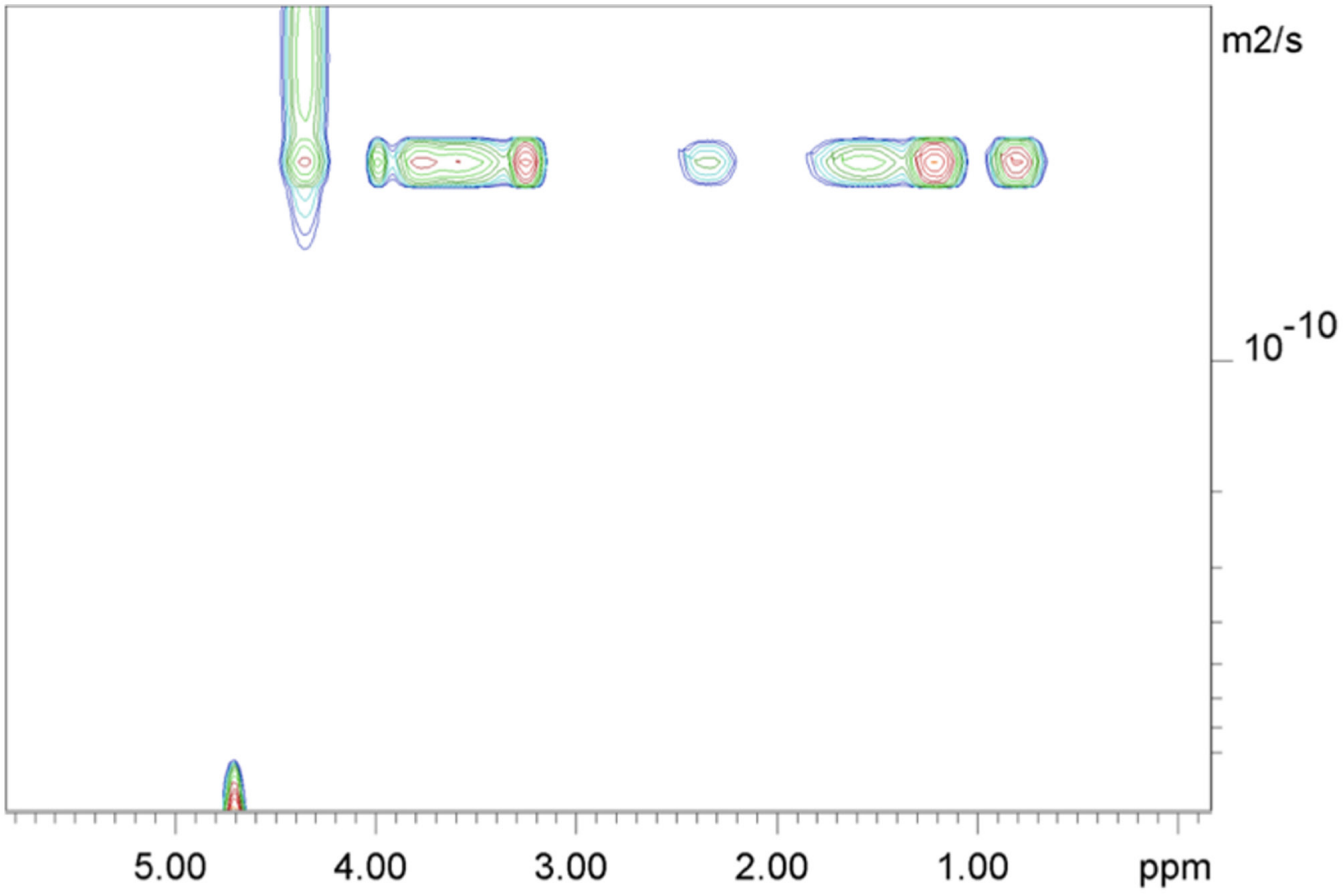
Two‐dimensional DOSY map of glycopeptoid **22c** in D_2_O *([**22c**] = 27 mM*).

Nevertheless, the two‐dimensional DOSY map showed a consistent pattern for all signals in the ^1^H NMR spectrum of glycopeptoid **22c**, indicating that above the CAC, the system shows up in the form of a single type of aggregates. A single diffusion coefficient was obtained, confirming that the system is highly organized (Figure 4).

Therefore, the measured diffusion coefficient for the derivative **22c** (*D = 3.5 × 10–11 m* ^*2*^ */s*) was used for a direct calculation of R_h_ using the Stokes–Einstein equation [80]. This correlation assumes a perfectly spherical aggregate and the solution viscosity equal to that of D_2_O, considering a not excessively high sample concentration. The hydrodynamic radius value obtained through the NMR DOSY sequence for the **22c** sample in D_2_O with a concentration of about 27 mM is approximately 5 nm, in agreement with DLS measurements.

#### 3.3.4 Preliminary Biological Screening

**FIGURE 5:**
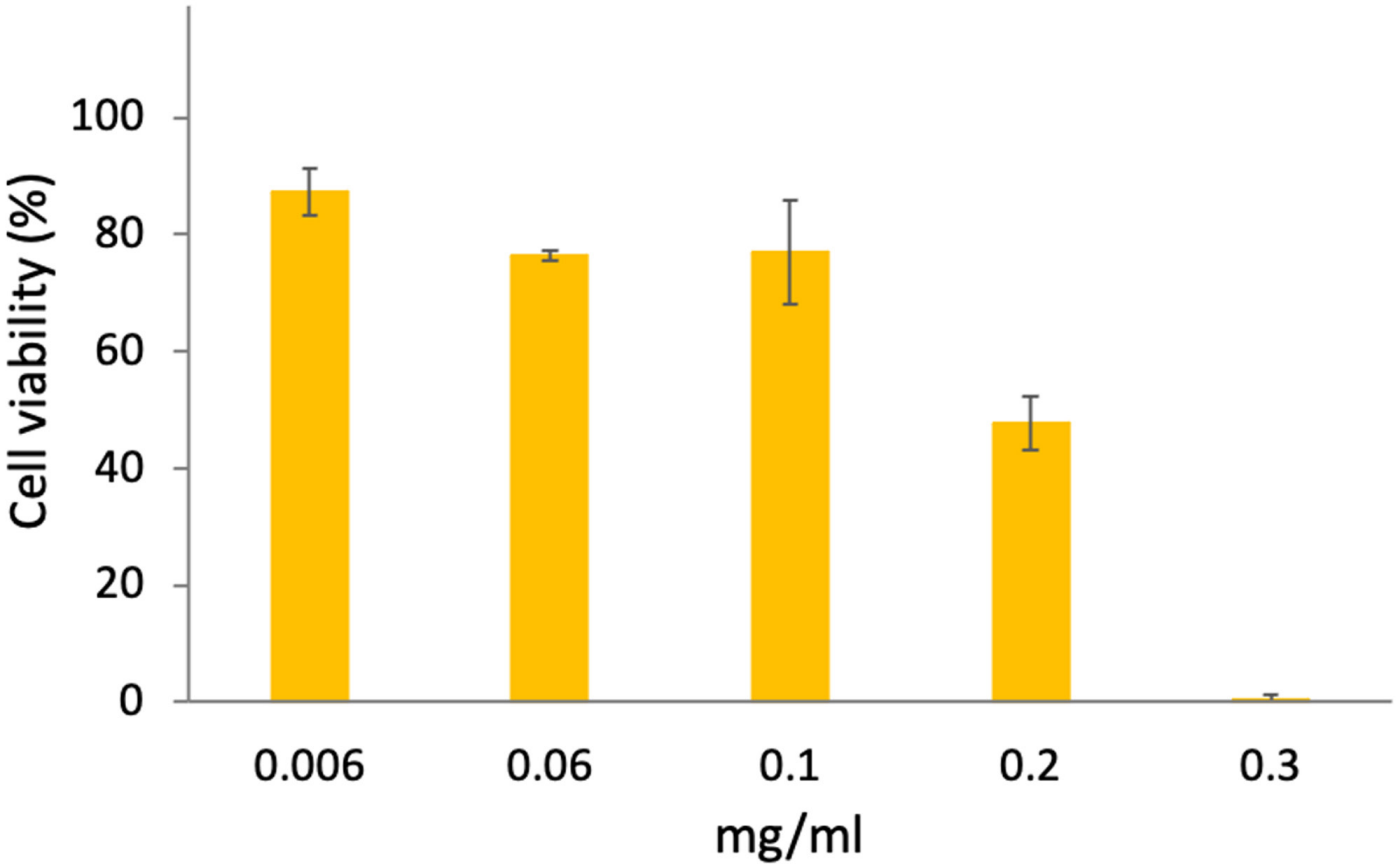
Cell viability on the Balb 3 T3 A31 cell line. The data represent the percentage of cell viability after 4 h of incubation with **22c** different concentrations. Means ± SD, *n* = 6.

The cytotoxic effects of **22c** on Balb/3T3 A31 fibroblast cells were evaluated by measuring cell viability after a 4‐h incubation with different sample concentrations (0.079–0.79 mM). The results, shown in Figure 5, illustrated a dose‐dependent effect of **22c** on cell viability. At lower concentrations (0.0079–0.13 mM), cell viability remained consistently high, around 85%, suggesting no cytotoxicity. Then, a lower cell viability was observed, reaching 50% at a concentration of 0.24 mM, which was identified as the IC_50_ value. This concentration is approximately three times higher than the CAC, suggesting that the compound maintains acceptable biocompatibility even in its aggregated state. Narrow functional concentration windows are commonly reported for amphiphilic and self‐assembling systems [9, 69], in which stable aggregation and biocompatibility typically occur only within a limited range close to the CAC. The present assay followed ISO guidelines for preliminary biocompatibility screening and, as such, does not provide mechanistic insight into the origin of cytotoxicity. Dedicated studies on membrane interactions, uptake pathways, and application‐specific cell lines will be required to elucidate these aspects in future work.

## 4 Conclusion

Herein, we optimized a convergent synthetic strategy to access a diverse library of glycopeptoids by comparing two different synthetic pathways. We characterized the self‐assembly properties of selected glycopeptoids and obtained preliminary biocompatibility data on murine fibroblasts. Although the biocompatible concentration window is relatively narrow, the results support further investigation of their structure–property relationships for potential biomedical and pharmaceutical relevance. Additionally, future studies will help the comprehension of the relation between the molecular structure of this family of glycopeptoids and their self‐assembly, possible solubilization effects, and interaction with biological systems, opening the way for the synthesis *on‐demand* of structured soft materials.

## Funding

This was supported by the Marie Skłodowska‐Curie Action (Grant Agreement number: 656157‐Pro‐Membrane‐H2020‐MSCA‐IF‐2014) and PNRR project “Tuscany Health Ecosystem (THE)” (project code: ECS00000017, CUP I53C22000780001).

## Supporting information


**Figure S1:** chir70121‐sup‐0001‐Supporting_Information.docx. ^1^H NMR spectrum of **3** in CDCl_3_ at 400 MHz.
**Figure S2:** chir70121‐sup‐0001‐Supporting_Information.docx. ^1^H NMR spectrum of **4** in CDCl_3_ at 400 MHz.
**Figure S3:** chir70121‐sup‐0001‐Supporting_Information.docx. ^13^C NMR spectrum of **4** in CDCl_3_ at 100 MHz.
**Figure S4:** chir70121‐sup‐0001‐Supporting_Information.docx. ^1^H NMR spectrum of **5** in CDCl_3_ at 400 MHz.
**Figure S5:** chir70121‐sup‐0001‐Supporting_Information.docx. ^13^C NMR spectrum of **5** in CDCl_3_ at 100 MHz.
**Figure S6:** COSY NMR bidimensional map of **5** in CDCl_3_ at 400 MHz.
**Figure S7:** chir70121‐sup‐0001‐Supporting_Information.docx. ^1^H NMR spectrum of **8** in CDCl_3_ at 400 MHz.
**Figure S8:** chir70121‐sup‐0001‐Supporting_Information.docx. ^13^C NMR spectrum of **8** in CDCl_3_ at 100 MHz.
**Figure S9:** chir70121‐sup‐0001‐Supporting_Information.docx. ^1^H NMR spectrum of **11a** in CDCl_3_ at 400 MHz.
**Figure S10:** ^13^C NMR spectrum of **11a** in CDCl_3_ at 400 MHz.
**Figure S11:** chir70121‐sup‐0001‐Supporting_Information.docx. ^1^H NMR spectrum of **11b** in CDCl_3_ at 500 MHz.
**Figure S12:** chir70121‐sup‐0001‐Supporting_Information.docx. ^13^C NMR spectrum of **11b** in CDCl_3_ at 500 MHz.
**Figure S13:** chir70121‐sup‐0001‐Supporting_Information.docx. ^1^H NMR spectrum of **11c** in CDCl_3_ at 500 MHz.
**Figure S14:** chir70121‐sup‐0001‐Supporting_Information.docx. ^13^C NMR spectrum of **11c** in CDCl_3_ at 500 MHz.
**Figure S15:** chir70121‐sup‐0001‐Supporting_Information.docx. ^1^H NMR spectrum of **12a** in CDCl_3_ at 400 MHz.
**Figure S16:** chir70121‐sup‐0001‐Supporting_Information.docx. ^1^H NMR spectrum of **12b** in CDCl_3_ at 400 MHz.
**Figure S17:** chir70121‐sup‐0001‐Supporting_Information.docx. ^1^H NMR spectrum of **12c** in CDCl_3_ at 400 MHz.
**Figure S18:** chir70121‐sup‐0001‐Supporting_Information.docx. ^1^H NMR spectrum of **13** in CDCl_3_ at 400 MHz.
**Figure S19:** chir70121‐sup‐0001‐Supporting_Information.docx. ^13^C NMR spectrum of **13** in CDCl_3_ at 100 MHz.
**Figure S20:** chir70121‐sup‐0001‐Supporting_Information.docx. ^1^H NMR spectrum of **16** in CDCl_3_ at 500 MHz.
**Figure S21:** chir70121‐sup‐0001‐Supporting_Information.docx. ^1^H NMR spectrum of **14** in CDCl_3_ at 500 MHz.
**Figure S22:** chir70121‐sup‐0001‐Supporting_Information.docx. ^13^C NMR spectrum of **14** in CDCl_3_ at 100 MHz.
**Figure S23:** chir70121‐sup‐0001‐Supporting_Information.docx. ^13^C NMR spectrum of **16** in CDCl_3_ at 125 MHz.
**Figure S24:** chir70121‐sup‐0001‐Supporting_Information.docx. ^1^H NMR spectrum of **17** in CDCl_3_ at 500 MHz.
**Figure S25:** chir70121‐sup‐0001‐Supporting_Information.docx. ^13^C NMR spectrum of **17** in CDCl_3_ at 125 MHz.
**Figure S26:** chir70121‐sup‐0001‐Supporting_Information.docx. ^1^H NMR spectrum of **19** in CDCl_3_ at 500 MHz.
**Figure S27:** Enlarged view of a selected region of the ^1^H NMR spectrum of **19** in CDCl_3_ at 500 MHz.
**Figure S28:** chir70121‐sup‐0001‐Supporting_Information.docx. ^13^C NMR spectrum of **19** in CDCl_3_ at 125 MHz.
**Figure S29:** chir70121‐sup‐0001‐Supporting_Information.docx. ^1^H NMR spectrum of **20** in CDCl_3_ at 400 MHz.
**Figure S30:** Enlarged view of a selected region of the ^1^H NMR spectrum of **20** in CDCl_3_ at 400 MHz.
**Figure S31:** chir70121‐sup‐0001‐Supporting_Information.docx. ^1^H NMR spectrum of **9** in CDCl_3_ at 400 MHz.
**Figure S32:** Enlarged view of a selected region of the ^1^H NMR spectrum of **9** in CDCl_3_ at 400 MHz.
**Figure S33:** chir70121‐sup‐0001‐Supporting_Information.docx. ^13^C NMR spectrum of **9** in CDCl_3_ at 100 MHz.
**Figure S34:** chir70121‐sup‐0001‐Supporting_Information.docx. ^1^H NMR spectrum of **21a** in CDCl_3_ at 500 MHz.
**Figure S35:** Enlarged view of a selected region of the ^1^H NMR spectrum of **21a** in CDCl_3_ at 500 MHz.
**Figure S36:** chir70121‐sup‐0001‐Supporting_Information.docx. ^13^C NMR spectrum of **21a** in CDCl_3_ at 125 MHz.
**Figure S37:** chir70121‐sup‐0001‐Supporting_Information.docx. ^1^H NMR spectrum of **21b** in CDCl_3_ at 500 MHz.
**Figure S38:** Enlarged view of a selected region of the ^1^H NMR spectrum of **21b** in CDCl_3_ at 500 MHz.
**Figure S39:** chir70121‐sup‐0001‐Supporting_Information.docx. ^13^C NMR spectrum of **21b** in CDCl_3_ at 125 MHz.
**Figure S40:** chir70121‐sup‐0001‐Supporting_Information.docx. ^1^H NMR spectrum of **22a** in CD_3_OD at 400 MHz.
**Figure S41:** chir70121‐sup‐0001‐Supporting_Information.docx. ^13^C NMR spectrum of **22a** in CD_3_OD at 125 MHz.
**Figure S42:** chir70121‐sup‐0001‐Supporting_Information.docx. ^1^H NMR spectrum of **22b** in CD_3_OD at 400 MHz.
**Figure S43:** chir70121‐sup‐0001‐Supporting_Information.docx. ^13^C NMR spectrum of **22b** in CD_3_OD at 125 MHz.
**Figure S44:** chir70121‐sup‐0001‐Supporting_Information.docx. ^1^H NMR spectrum of **22c** in CD_3_OD at 400 MHz.
**Figure S45:** chir70121‐sup‐0001‐Supporting_Information.docx. ^13^C NMR spectrum of **22c** in CD_3_OD at 125 MHz.
**Figure S46:** chir70121‐sup‐0001‐Supporting_Information.docx. ^13^C NMR spectrum of **22c** in D_2_O at 100 MHz.

## Data Availability

The data that support the findings of this study are openly available in figshare at https://figshare.com/account/articles/32390730%3Ffile%3D64905369, reference number 10.6084/m9.figshare.32390730.
