# Supplementary material for: A Convenient Strategy to Access Diverse Libraries of Amphiphilic Compounds Based on Glycopeptoids
Source: Chirality. 2026 Jul 9;38(7):e70121. doi: 10.1002/chir.70121 (PMC13347205; doi:10.1002/chir.70121)
Supplement: Supplementary file 1 — Figure S1: chir70121‐sup‐0001‐Supporting_Information.docx. 1H NMR spectrum of 3 in CDCl3 at 400 MHz. Figure S2: chir70121‐sup‐0001‐Supporting_Information.docx. 1H NMR spectrum of 4 in CDCl3 at 400 MHz. Figure S3: chir70121‐sup‐0001‐Supporting_Information.docx. 13C NMR spectrum of 4 in CDCl3 at 100 MHz. Figure S4: chir70121‐sup‐0001‐Supporting_Information.docx. 1H NMR spectrum of 5 in CDCl3 at 400 MHz. Figure S5: chir70121‐sup‐0001‐Supporting_Information.docx. 13C NMR spectrum of 5 in CDCl3 at 100 MHz. Figure S6: COSY NMR bidimensional map of 5 in CDCl3 at 400 MHz. Figure S7: chir70121‐sup‐0001‐Supporting_Information.docx. 1H NMR spectrum of 8 in CDCl3 at 400 MHz. Figure S8: chir70121‐sup‐0001‐Supporting_Information.docx. 13C NMR spectrum of 8 in CDCl3 at 100 MHz. Figure S9: chir70121‐sup‐0001‐Supporting_Information.docx. 1H NMR spectrum of 11a in CDCl3 at 400 MHz. Figure S10: 13C NMR spectrum of 11a in CDCl3 at 400 MHz. Figure S11: chir70121‐sup‐0001‐Supporting_Information.docx. 1H NMR spectrum of 11b in CDCl3 at 500 MHz. Figure S12: chir70121‐sup‐0001‐Supporting_Information.docx. 13C NMR spectrum of 11b in CDCl3 at 500 MHz. Figure S13: chir70121‐sup‐0001‐Supporting_Information.docx. 1H NMR spectrum of 11c in CDCl3 at 500 MHz. Figure S14: chir70121‐sup‐0001‐Supporting_Information.docx. 13C NMR spectrum of 11c in CDCl3 at 500 MHz. Figure S15: chir70121‐sup‐0001‐Supporting_Information.docx. 1H NMR spectrum of 12a in CDCl3 at 400 MHz. Figure S16: chir70121‐sup‐0001‐Supporting_Information.docx. 1H NMR spectrum of 12b in CDCl3 at 400 MHz. Figure S17: chir70121‐sup‐0001‐Supporting_Information.docx. 1H NMR spectrum of 12c in CDCl3 at 400 MHz. Figure S18: chir70121‐sup‐0001‐Supporting_Information.docx. 1H NMR spectrum of 13 in CDCl3 at 400 MHz. Figure S19: chir70121‐sup‐0001‐Supporting_Information.docx. 13C NMR spectrum of 13 in CDCl3 at 100 MHz. Figure S20: chir70121‐sup‐0001‐Supporting_Information.docx. 1H NMR spectrum of 16 in CDCl3 at 500 MHz. Figure S21: chir70121‐sup‐ [file CHIR-38-e70121-s001.docx]

**Supporting Information**

**Detailed experimental procedures, synthesis and characterization of compounds 1–22c.**

# A convenient strategy to access diverse libraries of amphiphilic compounds based on glyco-peptoids

Ludovica Dei^1^, Stefano Cadeddu^1^, Claude Taillefumier^2^, Chiara Migone^3^, Angela Fabiano^3^, Anna Maria Piras^3^, Maria Chiara Santangelo^3^, Sebastiano Di Pietro^3^*, Valeria Di Bussolo^3^* and Gaetano Angelici*^1^

Address: ^1^ Dipartimento di Chimica e Chimica Industriale, Università di Pisa, Via G. Moruzzi 13, 56124 Pisa, Italy. ^2^ Université Clermont Auvergne, Clermont Auvergne INP, CNRS, ICCF, 63000 Clermont-Ferrand, France. ^3^ Dipartimento di Farmacia, Università di Pisa, via Bonanno 33, 56126, Pisa, Italy

* Gaetano Angelici - [gaetano.angelici@unipi.it](mailto:gaetano.angelici@unipi.it)

* Valeria Di Bussolo - [valeria.dibussolo@unipi.it](mailto:valeria.dibussolo@unipi.it)

* Sebastiano Di Pietro - [sebastiano.dipietro@unipi.it](mailto:sebastiano.dipietro@unipi.it)

Summary

A convenient strategy to access diverse libraries of amphiphilic compounds based on glyco-peptoids S1

Synthetic procedures S4

Synthetic pathway A to obtain *N*,*N*-bis(2-hydroxyethyl)-2-(*N*-nonylacetamido)acetamide (5) S4

Synthesis of compound **1** S4

Synthesis of compound **2** S4

Synthesis of compound **3** S5

Synthesis of compound **4** S5

Synthesis of compound **5** S6

Synthetic pathway A for the double-glycosylation reaction S7

Synthesis of compound 6 and compound **7** S7

Synthesis of *N*,*N*-bis(2-hydroxyethyl)acetamide S7

Synthesis of compound **8** S8

Synthesis of 9 through Pathway A S9

Synthetic pathway B to synthesize the apolar tail S9

Synthesis of compound **10** S9

Synthesis of compound **11a** S9

Synthesis of compound **11b** S10

Synthesis of compound **11c** S11

Synthesis of compound **12a** S12

Synthesis of compound **12b** S12

Synthesis of compound **12c** S12

Synthesis of compound **13** S13

Synthesis of compound **14** S14

Synthesis of compound **15** S14

Synthesis of compound **16** S14

Synthesis of compound **17** S15

Synthetic Pathway B to synthetize the two-headed polar group S16

Synthesis of compound **19** S16

Synthesis of compound **20** S17

Convergent coupling reaction following pathway B S17

Synthesis of compound **9** S17

Synthesis of compound **21a** S18

Synthesis of compound **21b** S19

Final deprotection from benzoyl protecting groups S20

Synthesis of compound **22a** S20

Synthesis of compound **22b** S21

Synthesis of compound **22c** S21

NMR spectra S23

References S70

# Synthetic procedures

## Synthetic pathway A to obtain *N*,*N*-bis(2-hydroxyethyl)-2-(*N*-nonylacetamido)acetamide (5)

### Synthesis of compound 1


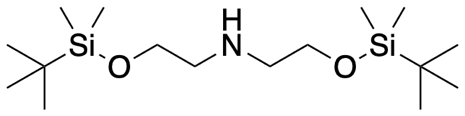
In a two-necked round-bottom flask, previously dried under nitrogen, diethanolamine (2.11 g, 20.1 mmol, 1.0 equiv.) was added followed by DMAP (490 mg, 4.01 mmol, 0.2 equiv.) and anhydrous CH_2_Cl_2_ (40 mL). Afterwards, a solution of *t*-butyldimethylsilyl chloride (6.52 g, 43.3 mmol, 2.1 equiv.) in CH_2_Cl_2_ (40 mL) was added dropwise at 0 °C. Upon completion of the addition, the reaction mixture was allowed to warm to room temperature and stirred overnight. Subsequently, the resulting mixture was filtered, and the filtrate was washed with saturated NaHCO_3_ (aq.) solution (x3). The combined organic layers were dried over Na_2_SO_4_ and concentrated under reduced pressure. The resulting crude residue was purified by flash chromatography on silica gel using 30% EtOAc in hexane to give the desired product 1 as yellow oil (5.96 g, 17.9 mmol, 89%). The product was used directly in the next reaction without further purification. Spectroscopic data in agreement with literature.[1]

### Synthesis of compound 2

**
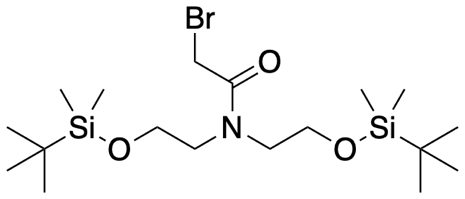
**In a two-necked round-bottom flask, previously dried under nitrogen, **1** (6.70 g, 20.1 mmol, 1.0 equiv.) was dissolved in anhydrous CH_2_Cl_2_ (75 mL) and Et_3_N (2.4 mL, 17 mmol, 0.8 equiv.) was added. Afterwards, bromoacetyl bromide (2.1 mL, 24 mmol, 1.2 equiv.) was added dropwise at 0 °C in 15 min. Upon completion of the addition, the reaction mixture was stirred for 1 h at -5 °C. Afterwards, the resulting mixture was filtered, and the solvent evaporated under reduced pressure. The resulting crude residue was purified by gradient flash chromatography on silica gel using 10-20% EtOAc in hexane to give the desired product **2** as yellow oil (6.13 g, 13.4 mmol, 67%). TLC R_f_ = 0.37 (Hex/EtOAc = 8:2 1H NMR (400 MHz, CDCl3) δ = 4.01 (s, 2H), 3.76 (dt, *J* = 6.9, 5.2 Hz, 4H), 3.63 (t, *J* = 5.1 Hz, 2H), 3.46 (t, *J* = 5.4 Hz, 2H), 0.87 (d, *J* = 3.9 Hz, 18H), 0.04 (d, *J* = 4.2 Hz, 12H). The product was directly use for the next step without further characterization.

### Synthesis of compound 3


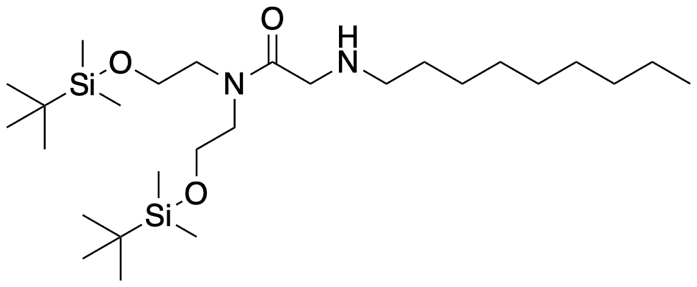
In a two-necked round-bottom flask, previously dried under nitrogen, **2** (4.70 g, 10.3 mmol, 1.0 equiv.) was dissolved in anhydrous THF (55 mL) and Et_3_N (2.9 mL, 21 mmol, 2.0 equiv.) was added. Subsequently, *n*-nonylamine (7.6 mL, 41.3 mmol, 4.0 equiv.) was added dropwise at 0 °C and the reaction mixture was stirred overnight at room temperature. Afterwards, the resulting mixture was filtered, and the solvent was evaporated under reduced pressure. The resulting crude residue was purified by gradient flash chromatography on silica gel using 30-50% EtOAc in hexane to give the desired product **3** as yellow oil (3.52 g, 6.82 mmol, 66%). ^1^H NMR (400 MHz, CDCl_3_) δ = 3.75 (t, *J* = 5.6 Hz, 2H), 3.70 (t, *J* = 5.6 Hz, 2H), 3.51 – 3.47 (m, 6H), 2.60 – 2.56 (m, 2H), 1.52 – 1.49 (m, 2H), 1.27 – 1.23 (m, 12H), 0.87 (d, *J* = 1.7 Hz, 21H), 0.04 (s, 6H), 0.03 (s, 6H). The product was directly use for the next step without further characterization.

### Synthesis of compound 4


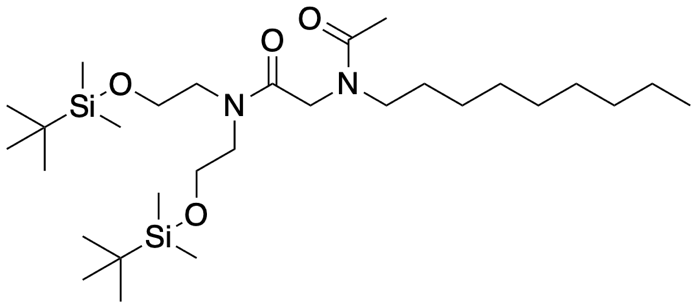
In a two-necked round-bottom flask, previously dried under nitrogen, **3** (3.52 g, 6.82 mmol, 1.0 equiv.) was dissolved in anhydrous EtOAc (50 mL) and Et_3_N (3.8 mL, 27 mmol, 4.0 equiv.) was added. Afterwards, acetic anhydride (5.2 mL, 54 mmol, 8.0 equiv.) was added dropwise in 15 min at room temperature. After stirring for 5 h at room temperature the solvent was evaporated under reduced pressure. The resulting crude residue was dissolved in CH_2_Cl_2_ and washed subsequently with HCl 1 N, saturated NaHCO_3_ (aq.) solution and water. The combined organic layers were dried over Na_2_SO_4_ and concentrated under reduced pressure. The crude residue was purified by gradient flash chromatography on silica gel using 40−50% EtOAc in hexane to give the desired product **4** as yellow oil (3.63 g, 6.65 mmol, 97%). TLC R_f_ = 0.60 (Hex/EtOAc = 1:1) ^1^H NMR (400 MHz, CDCl3) δ = 4.29 – 4.14 (m, 2H), 3.77 – 3.72 (m, 4H), 3.59 – 3.44 (m, 4H), 3.40 – 3.28 (m, 2H), 2.14 (s, 3H), 1.59 – 1.42 (bs, 2H), 1.25 (bs, 12H), 0.98 – 0.70 (m, 21H), 0.12 – 0.01 (m, 12H). ^13^C NMR (100 MHz, CDCl_3_) δ 174.40, 171.73, 171.52, 171.45, 171.22, 170.22, 170.01, 168.99, 168.81, 168.19, 61.63, 61.45, 61.27, 61.03, 60.94, 60.67, 60.56, 51.99, 51.86, 51.19, 50.81, 50.65, 50.47, 50.41, 50.33, 49.98, 49.79, 49.08, 48.74, 47.74, 47.65, 47.33, 47.18, 46.65, 31.91, 31.87, 29.62, 29.56, 29.43, 29.39, 29.32, 29.26, 28.64, 28.57, 28.52, 27.71, 27.03, 26.87, 26.01, 25.95, 25.90, 25.74, 25.58, 25.34, 22.90, 22.69, 21.58, 21.01, 20.92, 18.50, 18.35, 18.23, 18.06, 17.58, 14.15, -1.51, -3.56, -4.80, -5.34, -5.39, -5.43. HPLC-DAD purity 80%. ESI-HRMS [M+H]^+^ m/z = 559.43209 calculated for C29H62N2O4Si2 m/z = 558.42426

### Synthesis of compound 5


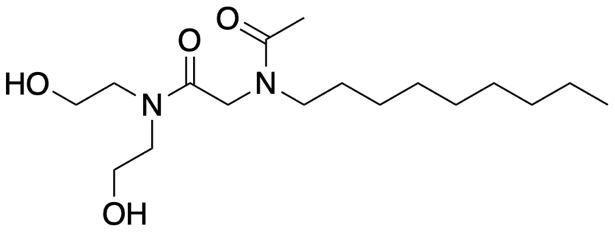
In a two-necked round-bottom flask, previously dried under nitrogen, **4** (2.76 g, 5.05 mmol, 1.0 equiv.) was dissolved in anhydrous THF (100 mL). Afterwards, *t*-butyl ammonium fluoride (15.2 mL, 15.2 mmol, 3.0 equiv.) was added at 0 °C and the reaction mixture was stirred overnight at room temperature. Then, the solvent was evaporated under reduced pressure, the resulting crude residue redissolved in CH_2_Cl_2_ and washed with saturated NaHCO_3_ (aq.) solution (x2) and brine. The combined organic layers were dried over Na_2_SO_4_ and concentrated under reduced pressure. The crude residue was purified by flash chromatography on silica gel using 20% MeOH in ethyl acetate to give the desired product 5 as yellow oil (1.45 g, 4.39 mmol, 87%). TLC R_f_ = 0.24 (EtOAc/MeOH = 8:2) 1H NMR (400 MHz, CDCl3) δ = 4.17 (s, 2H), 3.83 (t, *J* = 4.8 Hz, 4H), 3.56 – 3.49 (m, 4H), 3.42 – 3.36 (m, 2H), 2.15 (s, 3H), 1.60 – 1.53 (m, 2H), 1.32 – 1.20 (m, 12H), 0.87 (t, 3H). 13C NMR (100 MHz, CDCl3) δ = 171.73, 169.88, 60.73, 60.66, 60.62, 60.54, 59.65, 51.93, 51.86, 50.91, 50.84, 50.70, 50.64, 50.53, 50.25, 47.68, 47.63, 47.48, 38.69, 31.95, 31.90, 29.66, 29.59, 29.44, 29.35, 29.29, 28.60, 28.38, 27.68, 27.09, 26.91, 22.73, 22.71, 21.67, 21.07, 14.17, 14.15. The product was directly use for the next step without further characterization.

## Synthetic pathway A for the double-glycosylation reaction

### Synthesis of compound 6 and compound 7

In a two-necked round-bottom flask, previously dried under nitrogen, benzyl bis(2-hydroxyethyl)carbamate (189 mg, 0.79 mmol, 1.0 equiv.) and 2,3,4,6-tetra-*O*-acetyl-α-D-glucopyranosiltrichloroacetimidate (848 mg, 1.73 mmol, 2.2 equiv.) [2,3] were dissolved in dry CH_2_Cl_2_ (2.5 mL). Then, boron trifluoride etherate (174 μL, 1.58 mmol, 2.0 equiv.) was added and the reaction mixture was stirred overnight at room temperature. The resulting mixture was washed with saturated NaHCO_3_ (aq.), the combined organic layers were dried over Na_2_SO_4_ and concentrated under reduced pressure. The crude residue was purified by gradient flash chromatography on silica gel using 50-60% EtOAc in cyclohexane to afford **6** as colorless oil (158 mg, 0.18 mmol, 22%). Rf = 0.5 (30:70 Cyclohexane:EtOAc) ^1^H NMR (400 MHz, CDCl_3_) δ 7.42 – 7.30 (m, 5H), 5.26 – 4.91 (m, 8H), 4.48 (d, *J* = 7.9 Hz, 1H), 4.40 (d, *J* = 8.1 Hz, 1H), 4.35 – 4.18 (m, 3H), 4.00 – 3.83 (m, 3H), 3.79 – 3.64 (m, 3H), 3.64 – 3.46 (m, 3H), 3.45 – 3.31 (m, 2H), 2.07 – 1.95 (m, 24H). ESI-HRMS [M+H]^+^ *m/z* = 900.3164, [M+Na]^+^ *m/z* = 922.2853, calculated for C_40_H_54_NO_22_ *m/z* = 900.3137.

As subproduct was isolated from the same chromatography column **7** as colorless oil (131 mg, 0.21 mmol, 27%). Rf = 0.7 (30:70 Cyclohexane:EtOAc) ^1^H NMR (400 MHz, CDCl_3_) δ 7.41 – 7.29 (m, 5H), 5.25 – 4.92 (m, 5H), 4.49 (d, *J* = 7.8 Hz, 1H), 4.43 – 4.36 (m, 1H), 4.32 – 4.07 (m, 5H), 4.03 – 3.28 (m, 5H), 2.13 – 1.94 (m, 15H). ESI-HRMS [M+H]^+^ *m/z* = 612.2267, [M+Na]^+^ *m/z* = 634.2076, calculated for C_28_H_38_NO_14_ *m/z* = 612.2292 [M+H]^+^

### Synthesis of *N*,*N*-bis(2-hydroxyethyl)acetamide


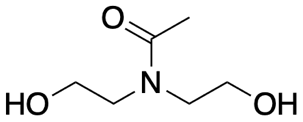
In a one-necked round-bottom flask diethanolamine (2.51 g, 23.8 mmol, 1.0 equiv.) was dissolved in THF (25 mL). Then, acetic anhydride (2.3 mL, 24 mmol, 1.0 equiv.) was added dropwise at 0 °C and, after completion of the addition, the reaction mixture was stirred under reflux for 1 h. The resulting reaction mixture was concentrated at 60 °C under reduced pressure for 1 h to remove acetic acid and *N*,*N*-bis(2-hydroxyethyl)acetamide was obtained as colorless oil (3.52 g, 23.9 mmol, quant.). The product was used directly in the next reaction without further purification. Spectroscopic data in agreement with literature.[4]

### Synthesis of compound 8


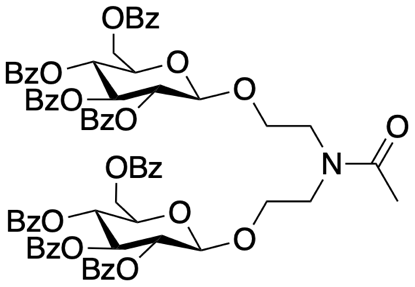


In a two-necked round-bottom flask, previously dried under nitrogen, *N*,*N*-bis(2-hydroxyethyl)acetamide (17.1 g, 0.115 mmol, 1.0 equiv.) and 2,3,4,6-tetra-*O*-benzoyl-a-D-glucopyranosiltrichloroacetimidate (209 mg, 0.282 mmol, 2.4 equiv.) were dissolved in dry CH_2_Cl_2_ (8 mL). Afterwards, boron trifluoride etherate (36.2 μL, 0.294 mmol, 2.5 equiv.) was added and the reaction mixture was stirred overnight at room temperature. The resulting mixture was washed subsequently with HCl 1 N, saturated NaHCO_3_ (aq.) solution and brine. The combined organic layers were dried over Na_2_SO_4_ and concentrated under reduced pressure. The crude residue was purified by gradient flash chromatography on silica gel using 20-100% EtOAc in hexane to give the desired product **8** as colorless oil (75 mg, 0.057 mmol, 50%). 1H NMR (400 MHz, CDCl3) δ = 8.05 – 7.99 (m, 4H), 7.98 – 7.93 (m, 2H), 7.92 – 7.87 (m, 6H), 7.83 – 7.78 (m, 4H), 7.57 – 7.45 (m, 6H), 7.43 – 7.29 (m, 14H), 7.28 – 7.20 (m, 5H), 5.91 (dt, *J* = 11.1, 9.6 Hz, 2H), 5.71 (dt, *J* = 9.7, 3.8 Hz, 2H), 5.61 – 5.46 (m, 2H), 4.76 (dd, *J* = 28.7, 7.9 Hz, 2H), 4.71 – 4.60 (m, 2H), 4.55 – 4.41 (m, 2H), 4.26 – 4.07 (m, 2H), 3.99 – 3.77 (m, 2H), 3.71 – 3.35 (m, 4H), 3.31 – 3.08 (m, 2H), 1.71 (s, 3H).^13^C NMR (125 MHz, CDCl3) δ 166.21, 166.17, 165.80, 165.76, 165.26, 165.20, 165.01, 133.56, 133.41, 133.36, 129.97, 129.95, 129.94, 129.83, 129.81, 128.62, 128.58, 128.54, 128.51, 128.38, 72.67, 72.44, 72.43, 71.85, 71.84, 71.50, 69.61, 69.45, 62.97, 62.96, 29.79. HPLC-DAD purity 82%. ESI-HRMS [M+H]^+^ m/z = 1304.41218 calculated for C74H65NO21 m/z = 1303.40436

## Synthesis of 9 through Pathway A


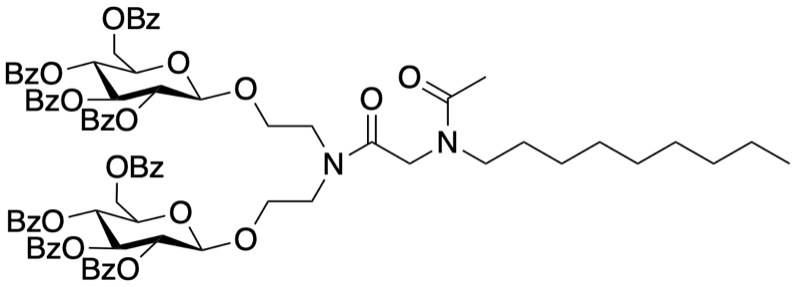
In a two-necked round-bottom flask, previously dried under vacuum, **5** (205 mg, 0.62 mmol, 1.0 equiv.) and 2,3,4,6-tetra-*O*-benzoyl-a-D-glucopyranosiltrichloroacetimidate (1.84 g, 2.48 mmol, 4.0 equiv.) were dissolved in anhydrous CH_2_Cl_2_ (40 mL). After stirring the mixture at room temperature for 10 min, BF_3_O⋅(CH_2_CH_3_)_2_ (200 μL, 1.62 mmol, 2.6 equiv.) was added dropwise and the reaction mixture was stirred overnight at room temperature. The solvent was removed under reduced pressure and the crude residue was purified by gradient flash chromatography on silica gel using 89-90% EtOAc in hexane to afford **9** as a white solid (520 mg, 0.35 mmol, 56%). See below for **9** characterization.

## Synthetic pathway B to synthesize the apolar tail

### Synthesis of compound 10


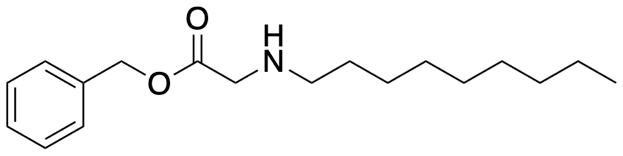
According to the procedure A, benzyl bromoacetate (**9**) (1.88 mL, 12 mmol, 1.2 equiv.) was dissolved in anhydrous THF (45 mL) in a two-necked round-bottom flask previously dried under nitrogen. Et_3_N (2.80 mL, 20 mmol, 2.0 equiv.) was added at 0 °C, followed by dropwise addition of *n*-nonylamine (1.83 mL, 10 mmol, 1.0 equiv.) at 0 °C. The reaction mixture was stirred overnight at room temperature. The resulting mixture was filtered through a plug of cotton, and the solvent was removed under reduced pressure. The crude residue was used directly in the next step without further purification or characterization (3.56 g, yellow oil). TLC R_f_ = 0.44 (Hex/EtOAc = 5:5)

### Synthesis of compound 11a


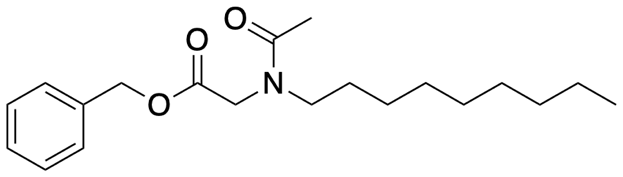
In a two-necked round-bottom flask, previously dried under nitrogen, the crude intermediate **10** (639 mg, 1.0 equiv.) was dissolved in anhydrous EtOAc (16 mL). Et_3_N (458 mL, 3.29 mmol, 1.5 equiv.) was added, followed by dropwise addition of acetic anhydride (310 mL, 3.29 mmol, 1.5 equiv.). The reaction mixture was stirred overnight at room temperature. The resulting mixture was concentrated under reduced pressure and redissolved in CH_2_Cl_2_ before being washed successively with HCl 1 N, saturated NaHCO_3_ (aq.) solution and brine. The combined organic layers were dried over Na_2_SO_4_ and concentrated under reduced pressure. The resulting crude residue was purified by gradient flash chromatography on silica gel using 30−60% EtOAc in hexane to give **11a** as yellow oil (351 mg, 1.05 mmol, 75% overall yield over two steps). TLC R_f_ = 0,33 (Hex/EtOAc = 6:4) ^1^H NMR (400 MHz, CDCl_3_) δ = 7.41 – 7.28 (m, 5H), 5.22 – 5.13 (2H (5.20, s + 5.16, s)), 4.17 – 3.96 (2H (4.09, s + 4.03, s)), 3.39 – 3.26 (2H (3.39 – 3.33, m + 3.33 – 3.26, m)), 2.18 – 1.98 (3H (2.15, s + 2.01, s)), 1.62 – 1.38 (3H (1.62 – 1.49, m + 1.49 – 1.38, m)), 1.36 – 1.14 (m, 12H), 0.94 – 0.82 (m, 3H). ^13^C NMR (100 MHz, CDCl_3_) δ = 171.10, 170.91, 169.48, 169.40, 135.62, 135.16, 128.84, 128.80, 128.70, 128.57, 128.46, 128.40, 67.44, 66.97, 50.74, 50.16, 47.62, 47.45, 31.96, 31.92, 29.62, 29.58, 29.51, 29.43, 29.35, 29.31, 28.76, 27.64, 26.98, 26.87, 22.77, 22.75, 21.73, 21.14, 14.21. UHPLC-ESI-MS (*m/z*) = 334 [M+H] ^+^ UHPLC-DAD purity = 100%

### Synthesis of compound 11b

According to the procedure B, DCC (895 mg, 4.34 mmol, 1.0 equiv.) was dissolved in anhydrous CH_2_Cl_2_ (30 mL) in a two-necked round-bottom flask previously dried under nitrogen. Butanoic acid (0.78 mL, 8.52 mmol, 2.0 equiv.) was then added, and the reaction mixture was stirred at room temperature for 1 h. The resulting mixture was filtered through a pad of Celite, and the filtrate, containing the butanoic anhydride, was used directly in the next step without further purification or characterization
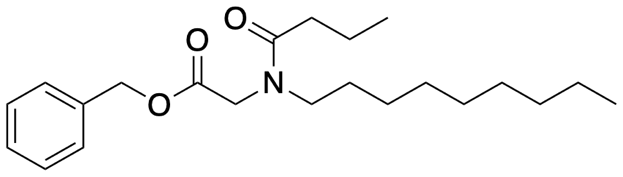
. According to the procedure C, the crude intermediate **10** (827 mg, 1.0 equiv.) was dissolved in anhydrous EtOAc (10 mL) in a two-necked round-bottom flask previously dried under nitrogen. Et_3_N (600 mL, 4.26 mmol, 1.5 equiv.) was added, followed by the dropwise addition of a solution of butanoic anhydride in CH_2_Cl_2_ (1.5 equiv.). The reaction mixture was stirred overnight at room temperature. The resulting mixture was washed successively with HCl 1 N, saturated NaHCO_3_ (aq.) solution and brine. The combined organic layers were over Na_2_SO_4_ and concentrated under reduced pressure. The resulting crude residue was purified by gradient flash chromatography on silica gel using 20−30% EtOAc in hexane to give **11b** as yellow oil (554 mg, 1.53 mmol, 66% overall yield over two steps)**.** TLC R_f_ = 0,26 (Hex/EtOAc = 8:2) ^1^H NMR (500 MHz, CDCl_3_) δ = 7.44 – 7.27 (m, 5H), 5.24 – 5.11 (2H (5.19, s + 5.15, s)), 4.14 – 4.00 (2H (4.09, s + 4.05, s)), 3.40 – 3.26 (2H (3.40 – 3.34, m + 3.34 – 3.26, m)), 2.37 – 2.11 (2H (2.37 – 2.32, m + 2.17 – 2.11, m)), 1.75 – 1.58 (m, 2H), 1.58 – 1.41 (2H (1.58 – 1.49 + 1.49 – 1.41, m)), 1.37 – 1.14 (m, 12H), 1.04 - 0.81 (6H (1.04 – 0.93, m + 0.93 – 0.81, m)). ^13^C NMR (125 MHz, CDCl_3_) δ = 173.60, 169.61, 135.65, 135.21, 128.83, 128.69, 128.59, 128.45, 128.43, 127.10, 67.39, 66.95, 49.94, 49.32, 47.82, 47.57, 35.17, 34.70, 31.97, 31.94, 29.63, 29.59, 29.51, 29.44, 29.36, 29.32, 28.94, 27.67, 27.01, 26.89, 26.52, 22.76, 18.77, 18.54, 14.22, 14.04, 13.96. UHPLC-ESI-MS (*m/z*) = 362 [M+H^+^]

### Synthesis of compound 11c

According to the procedure B, DCC (893 mg, 4.33 mmol, 1.0 equiv.) was dissolved in anhydrous CH_2_Cl_2_ (30 mL) in a two-necked round-bottom flask previously dried under nitrogen. Nonanoic acid (1.5 mL, 8.52 mmol, 2.0 equiv.) was then added, and the reaction mixture was stirred at room temperature for 1 h. The resulting mixture was filtered through a pad of Celite, and the filtrate, containing nonanoic anhydride, was used directly in the next step without further purification or characterization
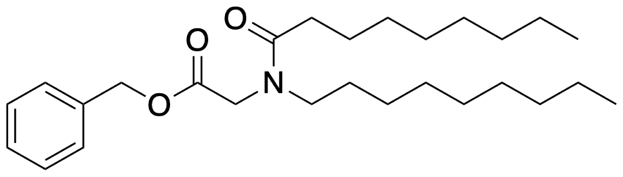
. According to the procedure C, the crude intermediate **10** (827 mg, 1.0 equiv.) was dissolved in anhydrous EtOAc (10 mL) in a two-necked round-bottom flask previously dried under nitrogen. Et_3_N (600 mL, 4.26 mmol, 1.5 equiv.) was then added, followed by the dropwise addition of a solution of nonanoic anhydride in CH_2_Cl_2_ (1.5 equiv). The reaction mixture was stirred overnight at room temperature. The resulting mixture was washed successively with HCl 1 N, saturated NaHCO_3_ (aq.) solution and brine. The combined organic layers were dried over Na_2_SO_4_ and concentrated under reduced pressure. The resulting crude residue was purified by flash chromatography on silica gel using 15% EtOAc in hexane to give **11c** as yellow oil (360 mg, 0.84 mmol, 30% overall yield over two steps). TLC R_f_ = 0.39 (Hex/EtOAc = 85:15) ^1^H NMR (500 MHz, CDCl_3_) δ = 7.43 – 7.28 (m, 5H), 5.21 – 5.12 (2H (5.19, s + 5.15, s)), 4.15 – 3.98 (2H (4.08, s + 4.04, s)), 3.42 – 3.24 (2H (3.42 – 3.33, m + 3.33 – 3.24, m)), 2.38 – 2.12 (2H (2.38 – 2.32, m + 2.18 – 2.12, m)), 1.72 – 1.41 (m, 4H), 1.40 – 1.14 (m, 22H), 0.94 – 0.80 (m, 6H). ^13^C NMR (125 MHz, CDCl_3_) δ = 173.74, 173.32, 169.62, 169.58, 135.66, 135.21, 128.81, 128.77, 128.67, 128.56, 128.42, 128.39, 67.35, 66.91, 49.96, 49.31, 47.82, 47.55, 33.30, 32.86, 31.97, 31.94, 31.01, 29.63, 29.58, 29.54, 29.50, 29.46, 29.43, 29.39, 29.35, 29.32, 29.29, 29.24, 28.93, 27.66, 27.00, 26.87, 26.51, 25.56, 25.34, 25.13, 24.84, 22.77, 22.76, 14.22. UHPLC-ESI-MS (*m/z*) = 432 [M+H^+^] UHPLC-DAD purity = 100%

### Synthesis of compound 12a


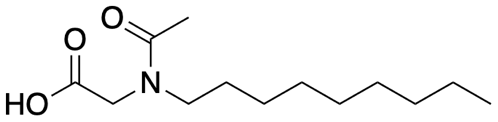
According to the procedure D, **11a** (280 mg, 0.84 mmol, 1.0 equiv.) was dissolved in MeOH (22 mL) in a three-necked round-bottom flask previously dried under nitrogen. Pd/C (29 mg, 10% w/w) was then added, and three vacuum/H_2_ cycles were performed. The reaction mixture was stirred overnight at room temperature under a H_2_-saturated atmosphere. The resulting mixture was filtered through a pad of Celite, and the solvent was removed under reduced pressure to afford **12a** as a yellow oil (176 mg, 0.7 mmol, 86%). ^1^H NMR (400 MHz, CDCl_3_) δ = 4.10 – 3.95 (2H (4.04, s + 4.00, s)), 3.40 – 3.27 (m, 2H), 2.21 – 2.02 (3H (2.17, s + 2.05, s)), 1.65 – 1.40 (2H (1.65 – 1.52, m + 1.52 – 1.40, m)), 1.37 – 1.16 (m, 12H), 0.94 – 0.78 (m, 3H). The product was directly use for the next step without further purification or characterization.

### Synthesis of compound 12b


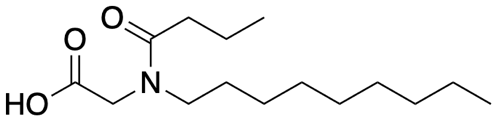
According to the procedure D, **11b** (339 mg, 0.94 mmol, 1.0 equiv.) was dissolved in MeOH (25 mL) in a three-necked round-bottom flask previously dried under nitrogen. Pd/C (35 mg, 10% w/w) was then added, and three vacuum/H_2_ cycles were performed. The reaction mixture was stirred overnight at room temperature under a H_2_-saturated atmosphere. The resulting mixture was filtered through a pad of Celite, and the solvent was removed under reduced pressure to afford **12b** as a yellow oil (239 mg, 0.88 mmol, 94%). ^1^H NMR (400 MHz, CDCl_3_) δ = 6.55 (bs, 1H), 4.13 – 3.94 (m, 2H), 3.44 – 3.23 (m, 2H), 2.47 – 2.15 (2H (2.47 – 2.29, m + 2.29 – 2.15, m)), 1.78 – 1.52 (3H (1.78 – 1.62, m + 1.62 – 1.52, m)), 1.37 – 1.17 (m, 13H), 1.03 – 0.83 (m, 6H). The product was directly use for the next step without further purification or characterization.

### Synthesis of compound 12c


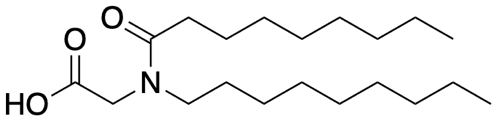
According to the procedure D, **11c** (324 mg, 0.75 mmol, 1.0 equiv.) was dissolved in MeOH (20 mL) in a three-necked round-bottom flask previously dried under nitrogen. Pd/C (32 mg, 10% w/w) was then added, and three vacuum/H_2_ cycles were performed. The reaction mixture was stirred overnight at room temperature under a H_2_-saturated atmosphere. The resulting mixture was filtered through a pad of Celite, and the solvent was removed under reduced pressure to afford **12c** as a yellow oil (244 mg, 0.71 mmol, 95%). ^1^H NMR (400 MHz, CDCl_3_) δ = 4.97 – 3.95 (m, 2H), 3.39 – 3.23 (m, 2H), 2.42 – 2.12 (2H (2.42 – 2.26, m + 2.26 – 2.12, m)), 1.70 – 1.46 (m, 4H), 1.31 – 1.17 (m, 22H), 0.93 – 0.79 (m, 6H). The product was directly use for the next step without further purification or characterization.

### Synthesis of compound 13

According to the procedure B, DCC (3.11 g, 15.00 mmol, 1.0 equiv.) was dissolved in anhydrous CH_2_Cl_2_ (100 mL) in a two-necked round-bottom flask previously dried under nitrogen. Bromoacetic acid (4.204 g, 30.00 mmol, 2.0 equiv.) was then added, and the reaction mixture was stirred at room temperature for 1 h. The resulting mixture was filtered through a pad of Celite, and the filtrate, containing 2-bromoacetic anhydride, was used directly in the next step without further purification or characterization
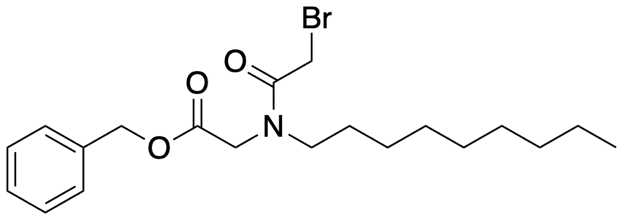
. According to the procedure C, the crude intermediate **10** (3.56 g, 1.0 equiv.) was dissolved in anhydrous EtOAc (45 mL) in a two-necked round-bottom flask previously dried under nitrogen. Et_3_N (2.10 mL, 15 mmol, 1.5 equiv) was then added, followed by the dropwise addition of a solution of bromoacetic anhydride in CH_2_Cl_2_ (1.5 equiv.). The reaction mixture was stirred overnight at room temperature. The resulting mixture was washed successively with HCl 1 N, saturated NaHCO_3_ (aq.) solution and brine. The combined organic layers were dried over Na_2_SO_4_ and concentrated under reduced pressure. The resulting crude residue was purified by gradient flash chromatography on silica gel using 15-25% EtOAc in hexane to give **13** as yellow oil (2.47 g, 6 mmol, 60% overall yield over two steps). TLC R_f_ = 0,24 (Hex/EtOAc = 85:15) ^1^H NMR (400 MHz, CDCl_3_) δ = 7.43 – 7.28 (m, 5H), 5.28 – 5.09 (2H (5.21, s + 5.17, s)), 4.24 – 4.03 (2H (4.16, s + 4.10, s)), 3.96 – 3.71 (2H (3.91, s + 3.76, s)), 3.48 – 3.25 (m, 2H), 1.75 – 1.38 (2H (1.75 – 1.54, m + 1.54 – 1.38, m)), 1.38 – 1.15 (m, 12H), 0.96 – 0.82 (m, 3H). ^13^C NMR (100 MHz, CDCl_3_) δ = 171.71, 169.02, 168.77, 167.90, 167.39, 167.24, 135.42, 134.94, 128.95, 128.91, 128.74, 128.69, 128.58, 128.52, 127.82, 127.15, 67.80, 67.24, 65.53, 50.64, 50.40, 49.84, 48.25, 48.17, 48.06, 31.97, 31.94, 29.57, 29.46, 29.39, 29.35, 29.31, 28.77, 28.71, 27.19, 26.87, 26.79, 26.40, 25.60, 25.52, 22.80, 22.78, 14.23. UHPLC-ESI-MS (m/z) = 412 [M+H^+^]

### Synthesis of compound 14

According to the procedure A, **13** (2.45 mg, 5.94 mmol, 1.2 equiv.) was dissolved in anhydrous THF (20 mL) in a two-necked round-bottom flask previously dried under nitrogen. Et_3_N (1.70 mL, 12.20 mmol, 2.4 equiv.) was then added at 0 °C, followed by the dropwise addition of *n*-nonylamine (910 μL, 5.00 mmol, 1.0 equiv.) at 0 °C. The reaction mixture was stirred at room temperature for 7 h. The resulting mixture was filtered through a plug of cotton, and the solvent was removed under reduced pressure. The crude residue was purified by gradient flash chromatography on silica gel using 30-100% EtOAc in petroleum ether to give **16** as a white solid (1.51 g, 4.13 mmol, 70%). ^1^H NMR (500 MHz, CDCl_3_) δ = 3.95 (s, 4H), 3.42 – 3.35 (m, 4H), 1.36 – 1.20 (m, 25H), 0.91 – 0.84 (m, 6H). ESI-MS (*m/z*) = 367 [M+H^+^] ^13^C NMR (100 MHz, CDCl_3_) δ 163.49, 49.95, 46.11, 31.90, 29.51, 29.34, 29.28, 26.78, 26.70, 22.72, 14.18. HPLC-DAD purity 92%. ESI-HRMS [M+H]+ m/z = 367.33191 [M+Na]+ m/z = 389.31385 [M+K]+ m/z = 405.28779 calculated for C22H42N2O2 m/z = 366.32408

### Synthesis of compound 15


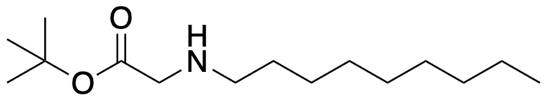
According to the procedure A, *t*-butyl bromoacetate (317 mg, 1.63 mmol, 1.2 equiv.) was dissolved in anhydrous THF (8 mL) in a two-necked round-bottom flask previously dried under nitrogen. Et_3_N (0.46 mL, 3.25 mmol, 2.0 equiv.) was added at 0 °C, followed by dropwise addition of *n*-nonylamine (0.25 mL, 1.35 mmol, 1.0 equiv.) at 0 °C. The reaction mixture was stirred overnight at room temperature. The resulting mixture was filtered through a plug of cotton, and the solvent was removed under reduced pressure. The crude residue was used directly in the next step without further purification or characterization (340 mg, colorless oil). TLC R_f_ = 0.32 (Hex/EtOAc = 8:2)

### Synthesis of compound 16

According to the procedure B, DCC (460 mg, 2.23 mmol, 1.0 equiv.) was dissolved in anhydrous CH_2_Cl_2_ (13 mL) in a two-necked round-bottom flask previously dried under nitrogen. Bromoacetic acid (560 mg, 4.00 mmol, 1.8 equiv.) was then added, and the reaction mixture was stirred at room temperature for 1 h. The resulting mixture was filtered through a pad of Celite, and the filtrate, containing 2-bromoacetic anhydride, was used directly in the next step without further purification or characterization
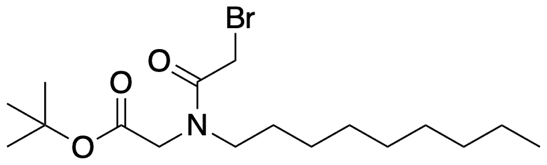
. According to the procedure C, the crude intermediate **15** (340 mg, 1.0 equiv.) was dissolved in anhydrous EtOAc (9 mL) in a two-necked round-bottom flask previously dried under nitrogen. Et_3_N (280 mL, 1.98 mmol, 1.5 equiv.) was then added, followed by the dropwise addition of a solution of 2-bromoacetic anhydride (1.5 equiv.). The reaction mixture was stirred overnight at room temperature. The resulting mixture was washed successively with HCl 1 N, saturated NaHCO_3_ (aq.) solution and brine. The combined organic layers were dried over Na_2_SO_4_ and concentrated under reduced pressure. The resulting crude residue was purified by flash chromatography on silica gel using 18% EtOAc in hexane to give **16** as colorless oil (309 mg, 0.82 mmol, 62% overall yield over two steps). TLC R_f_ = 0.34 (Hex/EtOAc = 82:18) ^1^H NMR (500 MHz, CDCl_3_): δ = 4.06 – 3.92 (2H (4.00, s + 3.95, s)), 3.92 – 3.74 (4H (3.90, s + 3.79, s)), 3.44 – 3.30 (m, 2H), 1.71 – 1.55 (m, 2H), 1.54 – 1.42 (9H (1.48, s + 1.46, s)), 1.38 – 1.12 (m, 12H), 0.94 – 0.82 (m, 3H). ^13^C NMR (125 MHz, CDCl_3_) δ = 168.23, 167.97, 167.14, 167.00, 83.08, 82.14, 51.29, 50.28, 48.73, 48.19, 31.98, 31.95, 29.59, 29.51, 29.43, 29.36, 29.32, 28.81, 28.18, 28.14, 27.33, 26.91, 26.86, 26.60, 25.82, 22.78, 14.24.

### Synthesis of compound 17


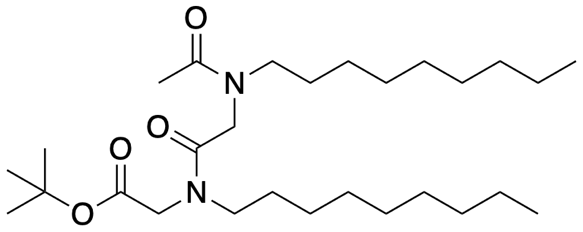
According to the procedure A, **16** (300 mg, 0.79 mmol, 1.2 equiv.) was dissolved in anhydrous THF (4 mL) in a two-necked round-bottom flask previously dried under nitrogen. Et_3_N (200 mL, 1.32 mmol, 2.0 equiv.) was then added at 0 °C, followed by the dropwise addition of *n*-nonylamine (0.12 mL, 0.66 mmol, 1.0 equiv.) at 0 °C. The reaction mixture was stirred at room temperature for 2.30 h. The resulting mixture was filtered through a plug of cotton, and the solvent was removed under reduced pressure. The crude residue of the intermediate *t*-butyl *N*-nonyl-*N*-(nonylglycyl)glycinate was used directly in the next step without further purification or characterization (315 mg, colorless oil). TLC R_f_ = 0.57 (Hex/EtOAc = 6:4)

In a two-necked round-bottom flask, previously dried under nitrogen, the crude intermediate *t*-butyl *N*-nonyl-*N*-(nonylglycyl)glycinate (315 mg, 1 equiv.) was dissolved in anhydrous EtOAc (5 mL). Et_3_N (400 μL, 2.86 mmol, 1.5 equiv.) was then added, followed by the dropwise addition of acetic anhydride (310 μL, 3.29 mmol, 1.5 equiv.). The reaction mixture was stirred overnight at room temperature. The resulting mixture was washed successively with HCl 1 N, saturated NaHCO_3_ (aq.) solution and brine. The combined organic layers were dried over Na_2_SO_4_ and concentrated under reduced pressure. The resulting crude residue was purified by gradient flash chromatography on silica gel using 30−60% EtOAc in hexane to give **17** as yellow oil (351 mg, 1.052 mmol, 35% overall yield over two steps). TLC R_f_ = 0.46 (Hex/EtOAc = 5:5) ^1^H NMR (500 MHz, CDCl_3_): δ = 4.40 – 3.79 (4H (4.22, s + 4.07, s + 4.00, s + 3.94, s)), 3.56 – 3.11 (m, 4H), 2.26 – 2.06 (3H (2.20, s + 2.19, s + 2.17, s + 2.08, s)), 1.66 – 1.52 (m, 3H), 1.52 – 1.41 (m, 8H), 1.40 – 1.14 (m, 25H), 0.99 – 0.72 (m, 6H). ^13^C NMR (125 MHz, CDCl_3_) δ = 171.63, 168.53, 82.76, 81.88, 50.00, 48.82, 48.76, 48.45, 46.53, 31.96, 29.63, 29.49, 29.35, 28.74, 28.46, 28.35, 28.18, 28.13, 27.64, 27.00, 26.95, 22.79, 20.84, 14.24. UHPLC-ESI-MS (*m/z*) = 483 [M+H^+^], 505 [M+Na^+^] UHPLC-DAD purity = 100%

## Synthetic Pathway B to synthetize the two-headed polar group

### Synthesis of compound 19


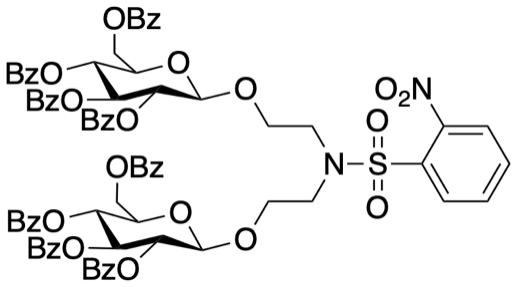
In a two-necked round-bottom flask, previously dried under nitrogen, 2,3,4,6-tetra-*O*-benzoyl-a-D-glucopyranosil trichloroacetimidate (3.16 g, 4.26 mmol, 3.1 equiv.) and diethane-2-nitrobenzenesulfonamide (398 mg, 1.37 mmol, 1.0 equiv.) were dissolved in anhydrous CH_2_Cl_2_ (62 mL). After stirring the mixture at room temperature for 10 min, BF_3_O⋅(CH_2_CH_3_)_2_ (340 μL, 2.66 mmol, 1.9 equiv.) was added dropwise at 0 °C. The reaction mixture was stirred overnight at room temperature. The resulting mixture was neutralized with Et_3_N and the solvent was removed under reduced pressure. The crude residue was purified by gradient flash chromatography on silica gel using 40-70% EtOAc in hexane to afford **19** as a white solid (1.57 g, 1.1 mmol, 79%). TLC R_f_ = 0.33 (Hex/EtOAc = 6:4) ^1^H NMR (500 MHz, CDCl_3_) δ = 8.03 (dd, *J* = 8.3, 1.4 Hz, 4H), 7.93 (ddd, *J* = 9.6, 8.3, 1.3 Hz, 9H), 7.82 (dd, *J* = 8.4, 1.4 Hz, 5H), 7.63 – 7.47 (m, 10H), 7.44 – 7.33 (m, 15H), 7.30 – 7.23 (m, 5H), 5.87 (t, *J* = 9.7 Hz, 2H), 5.67 (t, *J* = 9.7 Hz, 2H), 5.46 (dd, *J* = 9.8, 7.9 Hz, 2H), 4.74 (d, *J* = 7.9 Hz, 2H), 4.65 (dd, *J* = 12.1, 3.1 Hz, 2H), 4.46 (dd, *J* = 12.2, 5.1 Hz, 2H), 4.17 – 4.09 (m, 3H), 3.88 (dt, *J* = 10.2, 4.9 Hz, 2H), 3.61 (dt, *J* = 10.9, 5.6 Hz, 2H), 3.48 – 3.41 (m, 4H). ^13^C NMR (125 MHz, CDCl_3_) δ = 166.21, 166.14, 165.85, 165.29, 165.21, 165.18, 148.32, 147.96, 133.69, 133.59, 133.54, 133.49, 133.42, 133.36, 133.31, 133.18, 131.97, 131.85, 130.68, 130.37, 130.26, 129.95, 129.92, 129.85, 129.82, 129.79, 129.74, 129.63, 129.58, 129.34, 129.15, 129.04, 128.86, 128.78, 128.60, 128.57, 128.54, 128.44, 128.41, 124.31, 124.25, 101.17, 101.03, 72.96, 72.83, 72.46, 72.32, 71.80, 69.64, 69.42, 68.82, 63.07, 61.69, 48.19, 47.33, 31.05. ESI-HRMS [M+Na]^+^ *m/z* = 1469.36184, [M+K]^+^ *m/z* = 1485.33578, [M+Cl]^-^ *m/z* = 1481.34202, [M+HCOO]^-^ *m/z* = 1491.37083 calculated for C_78_H_66_N_2_O_24_S *m/z* = 1446.37207. UHPLC-DAD purity = 92%

### Synthesis of compound 20


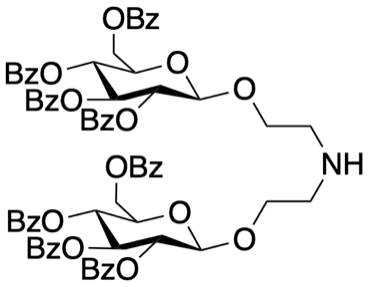
In a two-necked round-bottom flask, previously dried under vacuum, **19** (201 mg, 0.14 mmol, 1.0 equiv.) was added, and three vacuum/N_2_ cycles were performed. Anhydrous CH_3_CN (3.5 mL) was then added, followed by a freshly prepared 0.2 M solution of PhSH (2 mL, 0.41 mmol, 3.0 equiv.) in CH_3_CN. Finally, K_2_CO_3_ (85 mg, 0.61 mmol, 4.0 equiv.) was added, and the reaction mixture was stirred overnight at room temperature. The resulting mixture was diluted with EtOAc and filtered under reduced pressure. The solvent was removed under reduced pressure, and the crude residue was purified by gradient flash chromatography on silica gel: initially with 50% EtOAc in hexane to remove excess PhSH and byproducts, followed by 0-10 % MeOH in EtOAc to afford **20** as a white solid (172 mg, 0.14 mmol, 98%).^1^H NMR (400 MHz, CDCl_3_) δ = 8.02 (ddt, *J* = 6.7, 5.2, 1.3 Hz, 4H), 7.93 (ddt, *J* = 15.6, 8.6, 1.5 Hz, 8H), 7.82 (ddd, *J* = 8.5, 6.9, 1.4 Hz, 4H), 7.58 – 7.20 (m, 25H), 5.89 (td, *J* = 9.7, 7.3 Hz, 2H), 5.74 – 5.61 (m, 2H), 5.48 (dd, *J* = 9.8, 7.9 Hz, 2H), 5.00 – 4.57 (m, 3H), 4.55 – 4.38 (m, 2H), 4.33 – 4.08 (m, 3H), 3.95 (s, 1H), 3.86 – 3.69 (m, 1H), 3.55 (s, 1H), 3.04 – 2.93 (m, 1H), 2.92 – 2.70 (m, 2H). The product was directly use for the next step without further characterization.

## Convergent coupling reaction following pathway B

### Synthesis of compound 9


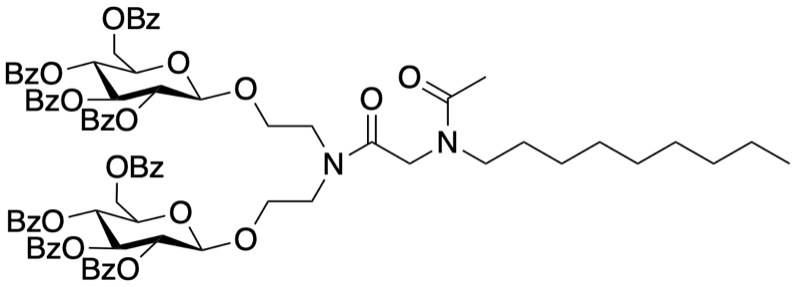
According to the procedure E, **12a** (94 mg, 0.38 mmol, 1.0 equiv.) was dissolved in anhydrous CH_2_Cl_2_ (28 mL) in a two-necked round-bottom flask previously dried under nitrogen. HATU (167 mg, 0.44 mmol, 1.1 equiv.) was then added, and the mixture was stirred at room temperature for 20 min. Subsequently, **20** (450 mg, 0.35 mmol, 1.0 equiv.) was added, followed by the dropwise addition of Et_3_N (170 mL, 1.21 mmol, 3.0 equiv.). The reaction mixture was stirred overnight at room temperature. The resulting mixture was diluted with CH_2_Cl_2_ and washed successively with HCl 1 N, saturated NaHCO_3_ (aq.) solution and brine. The combined organic layers were dried over Na_2_SO_4_ and concentrated under reduced pressure. The resulting crude residue was purified by gradient flash chromatography on silica gel using 20-0% hexane in EtOAc to afford **9** as a white solid (516 mg, 0.35 mmol, 97%). TLC R_f_ = 0.45 (Hex/EtOAc = 8:2) ^1^H NMR (400 MHz, CDCl_3_) δ = 8.18 – 7.09 (m, 40H), 5.95 – 5.84 (m, 2H), 5.74 – 5.57 (m, 2H), 5.57 – 5.36 (m, 2H), 4.81 – 4.57 (m, 4H), 4.56 – 4.35 (m, 2H), 4.43 – 3.10 (m, 10H), 2.10 – 1.83 (3H (2.08, s + 1.83,s)), 1.62 – 1.45 (m, 2H), 1.45 – 1.13 (m, 14H), 0.95 – 0.77 (m, 3H). ^13^C NMR (101 MHz, CDCl_3_) δ = 170.94, 168.96, 166.22, 165.85, 165.29, 165.19, 133.57, 133.45, 133.34, 129.97, 129.90, 129.87, 129.85, 129.50, 129.33, 129.22, 128.93, 128.71, 128.64, 128.59, 128.55, 128.46, 128.41, 128.20, 126.89, 101.64, 101.11, 72.92, 72.31, 72.05, 71.72, 69.70, 69.33, 67.60, 63.07, 50.36, 48.45, 47.35, 46.83, 38.82, 37.04, 31.99, 29.77, 29.61, 29.42, 28.74, 27.03, 22.79, 21.13, 14.24, 1.15.

ESI-HRMS [M+H]^+^ *m/z* = 1487.57450, [M+Na]^+^ *m/z* = 1509.55644, calculated for C_85_H_86_N_2_O_12_ m/z = 1486.56667. UHPLC-DAD purity 92%.

### Synthesis of compound 21a


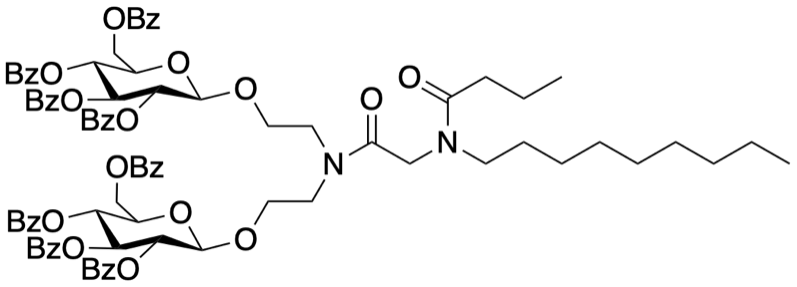
According to procedure E, **12b** (100 mg, 0.37 mmol, 1.0 equiv.) was dissolved in anhydrous CH₂Cl₂ (40 mL) in a two-necked round-bottom flask previously dried under nitrogen. HATU (156 mg, 0.41 mmol, 1.1 equiv.) was then added, and the mixture was stirred at room temperature for 20 min. Subsequently, **20** (455 mg, 0.36 mmol, 1.0 equiv.) was added, followed by the dropwise addition of Et_3_N (160 mL, 1.1 mmol, 3.0 equiv.). The reaction mixture was stirred overnight at room temperature. The resulting mixture was diluted with CH_2_Cl_2_ and washed successively with HCl 1 N, saturated NaHCO_3_ (aq.) solution and brine. The combined organic layers were dried over Na_2_SO_4_ and concentrated under reduced pressure. The resulting crude residue was purified by flash chromatography on silica gel using 50-0 % hexane in EtOAc to afford **21a** as a white solid (460 mg, 0.3 mmol, 83%). TLC R_f_ = 0.19 (Hex/EtOAc = 5:5) R_f_ = 0.7 (Hex/EtOAc = 3:7) ^1^H NMR (500 MHz, CDCl_3_) δ = 8.12 – 7.08 (m, 40H), 6.03 – 5.78 (m, 2H), 5.76 – 5.57 (m, 2H), 5.56 – 5.34 (m, 2H), 4.81 – 4.54 (m, 4H), 4.53 – 4.39 (m, 2H), 4.32 – 3.37 (m, 9H), 3.37 – 3.03 (m, 3H), 2.38 – 2.22 (m, 2H), 2.02 – 1.77 (m, 1H), 1.76 – 1.58 (m, 2H), 1.58 – 1.45 (m, 2H), 1.45 – 1.07 (m, 13H), 1.00 – 0.91 (m, 3H), 0.91 – 0.76 (m, 3H). ^13^C NMR (126 MHz, CDCl_3_) δ = 173.27, 169.17, 166.20, 165.85, 165.82, 165.29, 165.25, 165.16, 165.07, 133.53, 133.42, 133.31, 133.27, 133.25, 129.98, 129.94, 129.87, 129.85, 129.82, 129.69, 129.66, 129.34, 129.22, 128.93, 128.67, 128.60, 128.56, 128.52, 128.43, 128.38, 101.59, 101.06, 72.94, 72.72, 72.27, 72.24, 72.07, 71.72, 69.76, 69.72, 69.57, 69.14, 67.63, 63.08, 49.48, 48.55, 47.51, 46.92, 34.68, 31.98, 29.74, 29.58, 29.47, 29.39, 28.91, 27.03, 22.76, 18.81, 18.49, 14.21, 14.12, 14.09. ESI-HRMS [M+H]^+^ *m/z* = 1515.60580, [M+Na]^+^ *m/z* = 1537.58774, calculated for C_87_H_90_N_2_O_22_ m/z = 1514.59797. UHPLC-DAD purity = 89%

### Synthesis of compound 21b


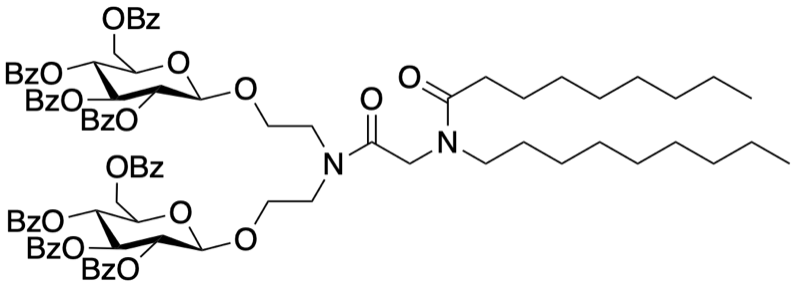
According to the procedure E, **12c** (108 mg, 0.32 mmol, 1.0 equiv.) was dissolved in anhydrous CH_2_Cl_2_ (23 mL) in a two-necked round-bottom flask previously dried under nitrogen. HATU (141 mg, 0.37 mmol, 1.1 equiv.) was then added, and the mixture was stirred at room temperature for 20 min. Subsequently, **20** (397 mg, 0.32 mmol, 1.0 equiv.) was added, followed by the dropwise addition of Et_3_N (140 mL, 1.00 mmol, 3.0 equiv.). The reaction mixture was stirred overnight at room temperature. The resulting mixture was diluted with CH_2_Cl_2_ and washed successively with HCl 1 N, saturated NaHCO_3_ (aq.) solution and brine. The combined organic layers were dried over Na_2_SO_4_ and concentrated under reduced pressure. The resulting crude residue was purified by flash chromatography on silica gel using 50 % EtOAc in hexane to afford **21b** as a white solid (438 mg, 0.28 mmol, 88%). TLC R_f_ = 0.45 (Hex/EtOAc = 5:5) R_f_ = 0.25 (Hex/EtOAc = 55:45) ^1^H NMR (500 MHz, CDCl_3_) δ 8.10 – 7.17 (m, 40H), 5.98 – 5.80 (m, 2H), 5.76 – 5.57 (m, 2H), 5.55 – 5.37 (m, 2H), 4.78 – 4.59 (m, 4H), 4.51 – 4.40 (m, 2H), 4.15 – 4.06 (m, 2H), 4.05 – 3.98 (m, 1H), 3.90 – 3.79 (m, 1H), 3.77 – 3.67 (m, 2H), 3.65 – 3.39 (m, 3H), 3.31 – 3.10 (m, 3H), 2.36 – 2.25 (m, 2H), 1.76 (bs, 1H), 1.68 – 1.46 (m, 3H), 1.45 – 1.14 (m, 24H), 0.98 – 0.70 (m, 6H). ^13^C NMR (125 MHz, CDCl_3_) δ = 173.48, 169.16, 166.21, 165.83, 165.30, 165.17, 165.09, 133.54, 133.42, 133.32, 130.00, 129.95, 129.89, 129.86, 129.84, 129.70, 129.34, 129.23, 128.94, 128.68, 128.57, 128.53, 128.39, 101.61, 101.08, 72.95, 72.27, 72.06, 71.73, 69.76, 69.71, 69.22, 67.66, 63.06, 49.48, 48.50, 47.49, 46.90, 32.87, 31.99, 29.76, 29.66, 29.59, 29.55, 29.42, 29.34, 28.91, 27.03, 25.40, 22.79, 14.24. ESI-HRMS [M+H]^+^ *m/z* = 1585.68405, [M+Na]^+^ *m/z* = 1607.66599, [M+K]^+^ *m/z* = 1623.63993, calculated for C_92_H_100_N_2_O_22_ *m/z* = 1584.67622. UHPLC-DAD purity 92%.

## Final deprotection from benzoyl protecting groups

### Synthesis of compound 22a


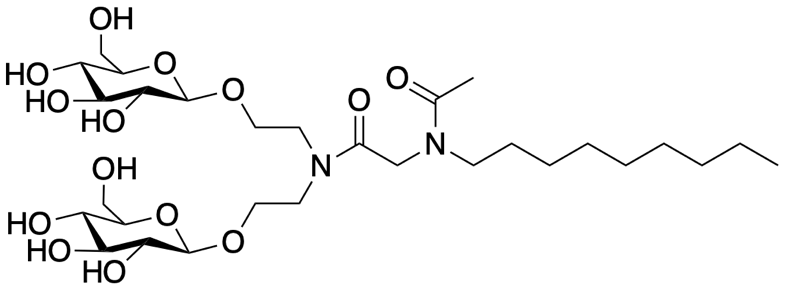
According to the procedure F, **21a** (494 mg, 0.33 mmol, 1.0 equiv.) was dissolved in MeOH (5 mL) in a one-necked round-bottom flask. A freshly prepared 0.39 M solution of sodium methoxide in MeOH (13 mL, 5.1 mmol, 15 equiv.) was then added dropwise, and the reaction mixture was stirred at room temperature for 3 h under air. Amberlite IR 120 resin (previously washed with MeOH) was then added until the pH reached neutrality and was subsequently removed by filtration. The solvent was removed under reduced pressure, and the crude residue was purified by trituration with isopropyl ether to remove methyl benzoate. **22a** was obtained as a white solid (206 mg, 0.31 mmol, 95%). 1H NMR (400 MHz, CD_3_OD) δ = 4.52 – 4.21 (m, 4H), 4.08 – 3.97 (m, 1H), 3.94 – 3.08 (m, 19H), 2.22 – 1.92 (3H (2.14, s + 1.98, s)), 1.69 -1.46 (m, 2H), 1.43 – 1.21 (m, 14H), 0.990 – 0.844 (m, 3H). 13C-NMR (125 MHz, CD_3_OD) δ = 174.67, 173.69, 171.54, 171.46, 130.48, 129.59, 105.11, 104.97, 104.77, 104.64, 78.16, 78.10, 78.04, 78.01, 77.99, 77.87, 75.08, 71.61, 71.55, 71.47, 68.96, 68.75, 68.63, 68.61, 62.74, 62.69, 62.63, 51.78, 51.48, 47.58, 47.13, 33.08, 33.06, 30.75, 30.68, 30.61, 30.46, 30.43, 29.50, 28.64, 28.08, 27.94, 23.74, 21.89, 21.07, 17.28, 14.46. ESI-HRMS [M+H]^+^ *m/z* = 655.36478, [M+Na]^+^ *m/z* = 677.34673, calculated for C_29_H_54_N_2_O_14_ *m/z* = 654.35696.

### Synthesis of compound 22b


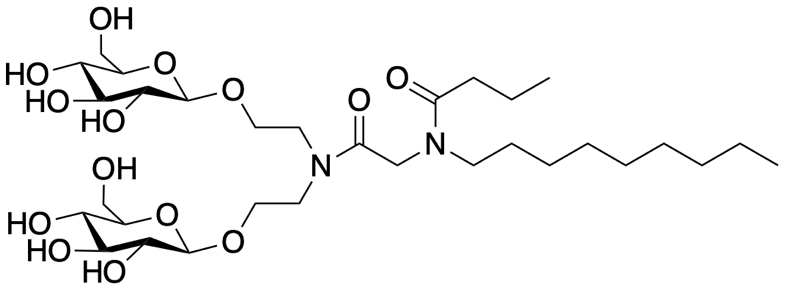
According to the procedure F, **21b** (295 mg, 0.19 mmol, 1.0 equiv.) was dissolved in MeOH (1 mL) in a one-necked round-bottom flask. A freshly prepared 0.39 M solution of sodium methoxide in MeOH (7 mL, 2.7 mmol, 14 equiv.) was then added dropwise, and the reaction mixture was stirred at room temperature for 3 h under air. Amberlite IR 120 resin (previously washed with MeOH) was then added until the pH reached neutrality and was subsequently removed by filtration. The solvent was removed under reduced pressure, and the crude residue was purified by trituration with isopropyl ether to remove methyl benzoate. **22b** was obtained as a white solid (127 mg, 0.18 mmol, 96%). ^1^H NMR (400 MHz, CD_3_OD) δ = 4.51 – 4.19 (m, 4H), 4.09 – 3.15 (m, 20H), 2.47 – 2.34 (m, 1H), 2.22 – 2.16 (m, 1H), 1.71 – 1.49 (m, 4H), 1.35 – 1.29 (m, 14H), 1.00 – 0.88f (m, 6H). ^13^C NMR (125 MHz, CD_3_OD) δ = 176.57, 175.87, 171.66, 171.50, 105.19, 104.96, 104.82, 104.68, 104.62, 78.11, 78.03, 77.97, 77.87, 75.07, 71.60, 71.52, 71.49, 69.10, 68.76, 68.68, 68.63, 68.43, 62.74, 62.67, 60.65, 60.52, 51.00, 50.64, 50.16, 48.02, 47.55, 47.19, 36.05, 35.88, 35.58, 33.07, 33.05, 30.75, 30.70, 30.68, 30.57, 30.52, 30.44, 30.41, 29.71, 29.61, 28.66, 28.51, 28.09, 28.04, 27.89, 23.73, 19.85, 19.81, 19.61, 14.46, 14.31, 14.25, 14.20. ESI-HRMS [M+H]^+^ *m/z* = 683.39608, [M+Na]^+^ *m/z* = 705.37803, calculated for C_31_H_58_N_2_O_14_ *m/z* = 682.38826.

### Synthesis of compound 22c


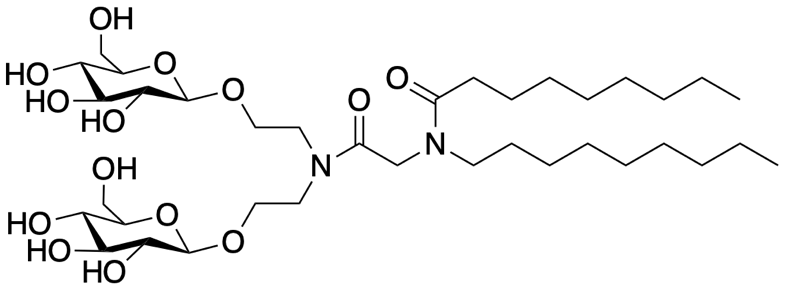
According to the procedure F, **21c** (499 mg, 0.31 mmol, 1.0 equiv.) was dissolved in MeOH (4 mL) in a one-necked round-bottom flask. A freshly prepared 0.39 M solution of sodium methoxide in MeOH (13 mL, 5.1 mmol, 16 equiv.) was then added dropwise, and the reaction mixture was stirred at room temperature for 3 h under air. Amberlite IR 120 resin (previously washed with MeOH) was then added until the pH reached neutrality and was subsequently removed by filtration. The solvent was removed under reduced pressure, and the crude residue was purified by trituration with isopropyl ether to remove methyl benzoate. **22c** was obtained as a white solid (217 mg, 0.29 mmol, 92%). ^1^H NMR (400 MHz, CD_3_OD) δ = 4.53 – 4.20 (m, 4H), 4.09 – 3.05 (m, 20H), 2.51 – 2.33 (m, 1H), 2.29 – 2.14 (m, 1H), 1.74 – 1.45 (m, 4H), 1.32 (m, 26H), 1.02 – 0.76 (m, 6H). ^13^C NMR (125 MHz, CD_3_OD) δ = 176.76, 176.07, 171.65, 171.50, 105.20, 104.97, 104.82, 104.64, 78.14, 78.12, 78.04, 78.02, 77.98, 77.88, 75.09, 75.06, 71.60, 71.55, 71.53, 69.11, 68.77, 68.73, 68.64, 62.74, 62.67, 57.46, 51.01, 50.66, 47.56, 47.24, 34.05, 33.70, 33.09, 33.07, 30.78, 30.76, 30.71, 30.55, 30.50, 30.46, 30.43, 30.39, 30.36, 29.69, 28.67, 28.09, 27.87, 26.52, 26.41, 23.87, 23.74, 14.47. ^13^C NMR (101 MHz, D_2_O) δ = 175.18, 170.15, 103.09, 102.62, 76.07, 75.89, 73.21, 70.02, 69.73, 67.80, 67.51, 61.21, 60.94, 49.39, 48.00, 46.45, 32.50, 32.02, 31.98, 29.67, 29.47, 29.28, 28.51, 26.63, 25.36, 22.72, 22.69, 13.92, 13.87. ESI-HRMS [M+H]^+^ *m/z* = 753.47433, [M+Na]^+^ *m/z* = 775.45628, calculated for C_36_H_68_N_2_O_14_ *m/z* = 752.46651. UHPLC-DAD purity 100%. CAC (20 °C) = 0.08 mM (0.06 mg/mL). UHPLC-DAD purity 100%.

## NMR spectra

**Figure S1.** ^1^H NMR spectrum of **3** in CDCl_3_ at 400 MHz

**
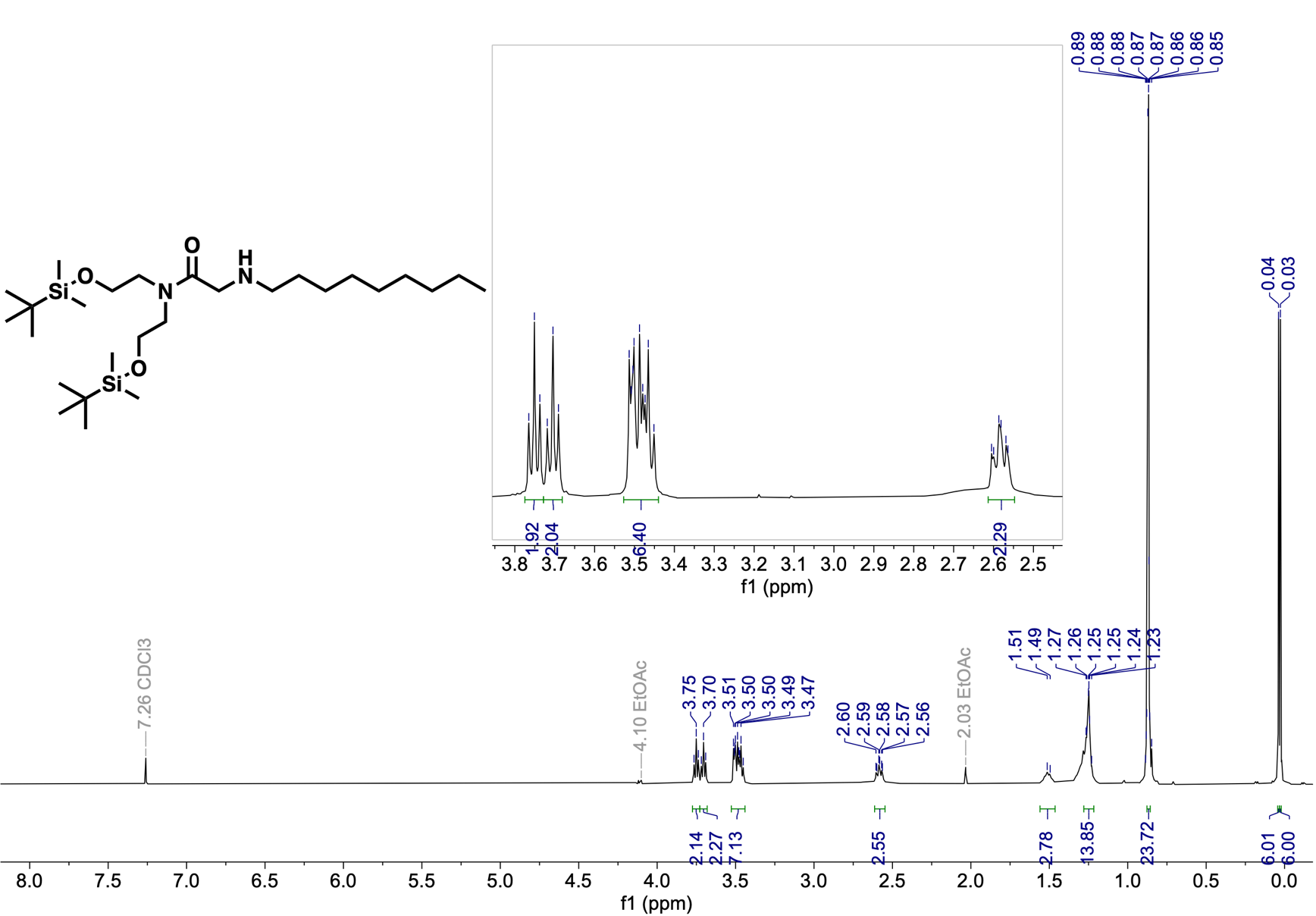
**

**Figure S2.** ^1^H NMR spectrum of **4** in CDCl_3_ at 400 MHz

**
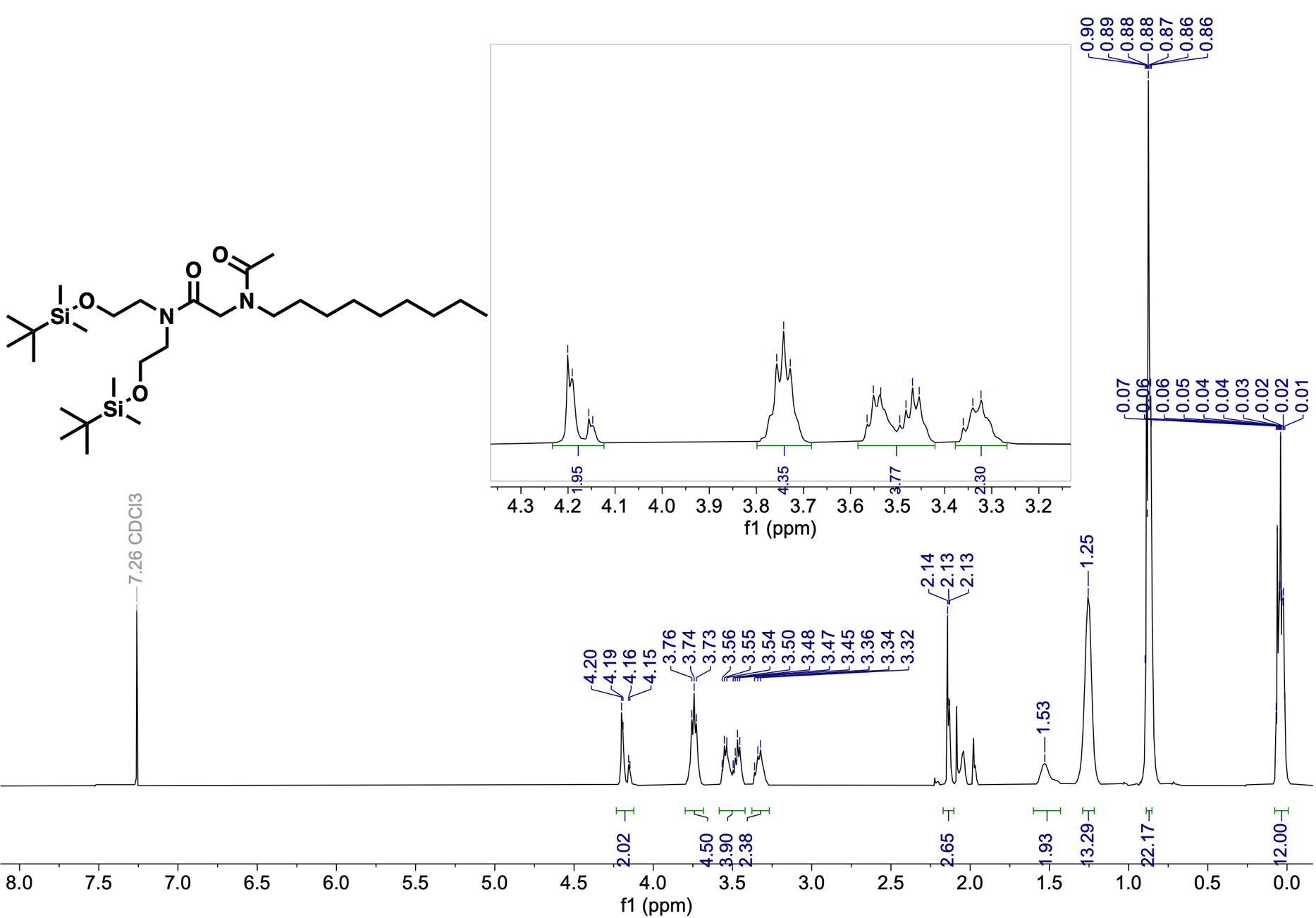
**

**Figure S3.** ^13^C NMR spectrum of **4** in CDCl_3_ at 100 MHz

**
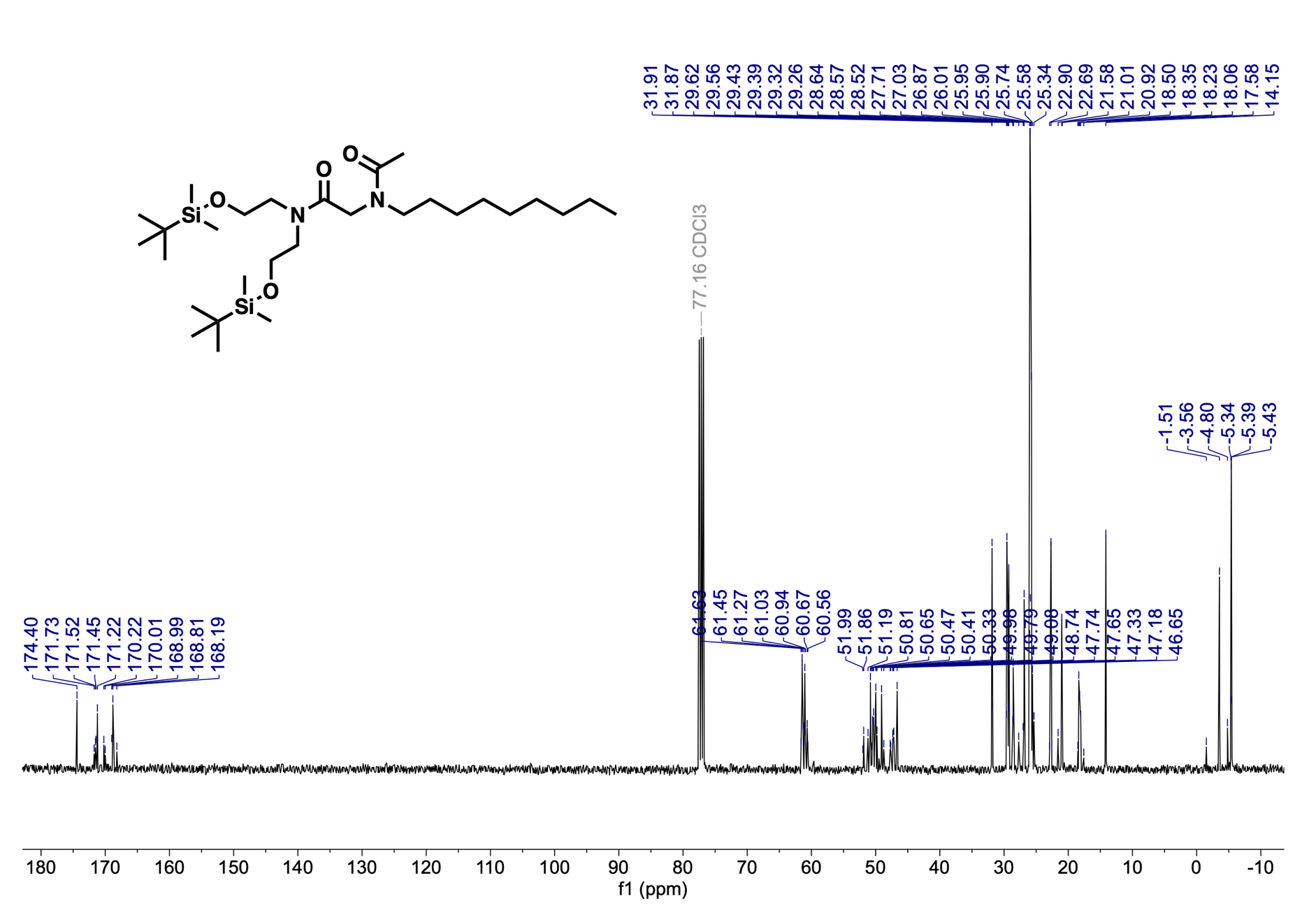
**

**Figure S4.** ^1^H NMR spectrum of **5** in CDCl_3_ at 400 MHz

**
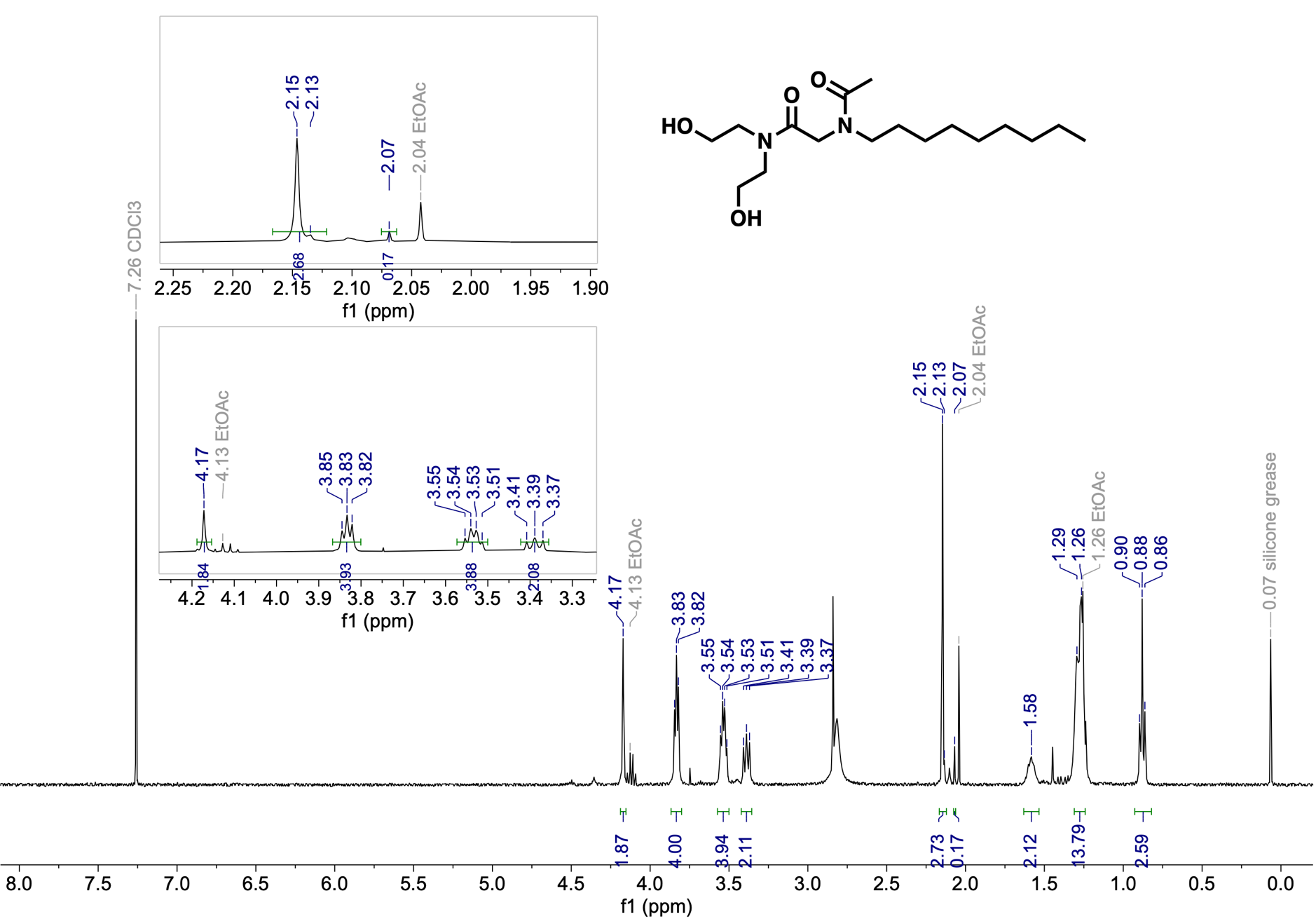
**

**Figure S5.** ^13^C NMR spectrum of **5** in CDCl_3_ at 100 MHz

**
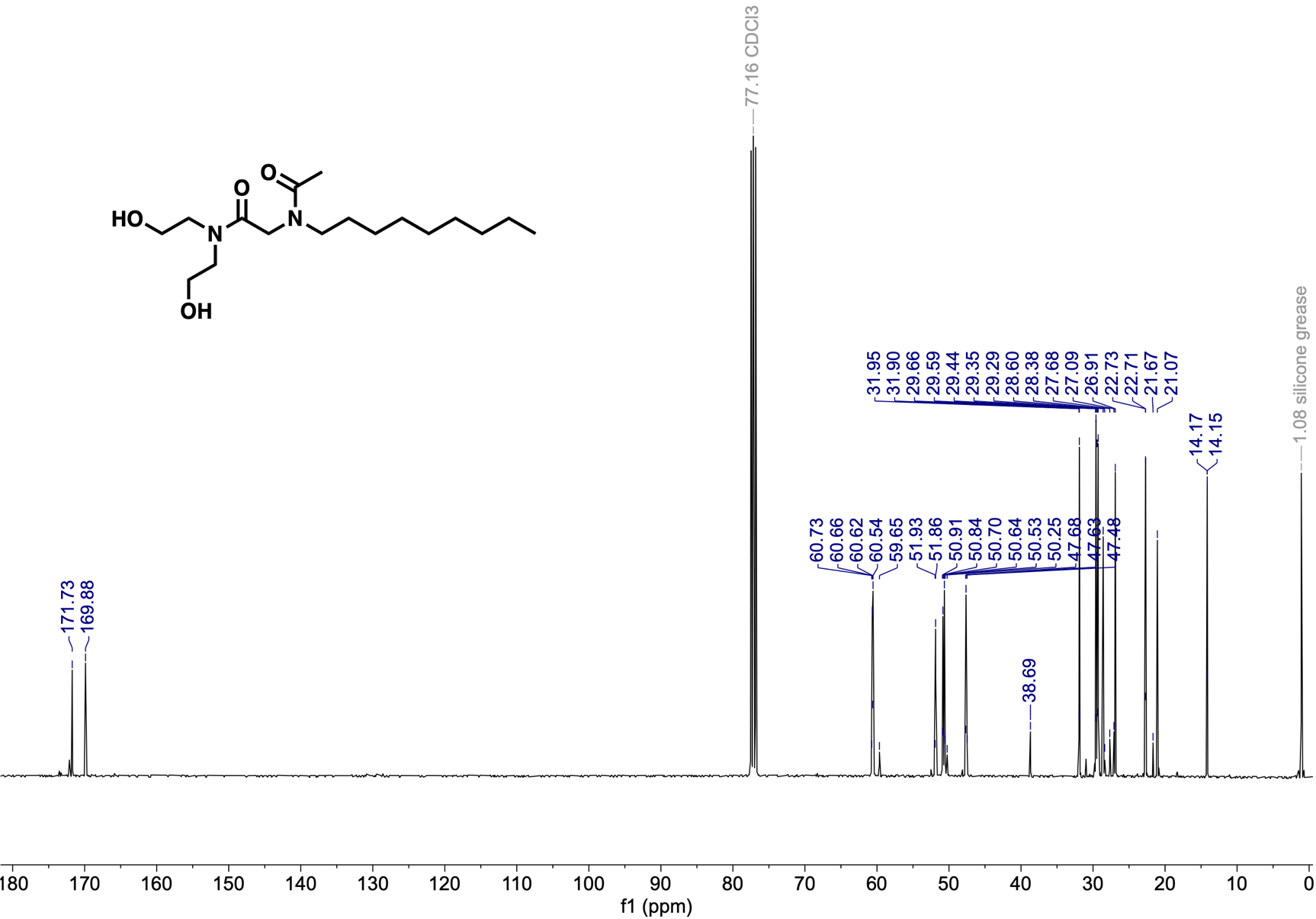
**

**Figure S6.** COSY NMR bidimensional map of **5** in CDCl_3_ at 400 MHz

**
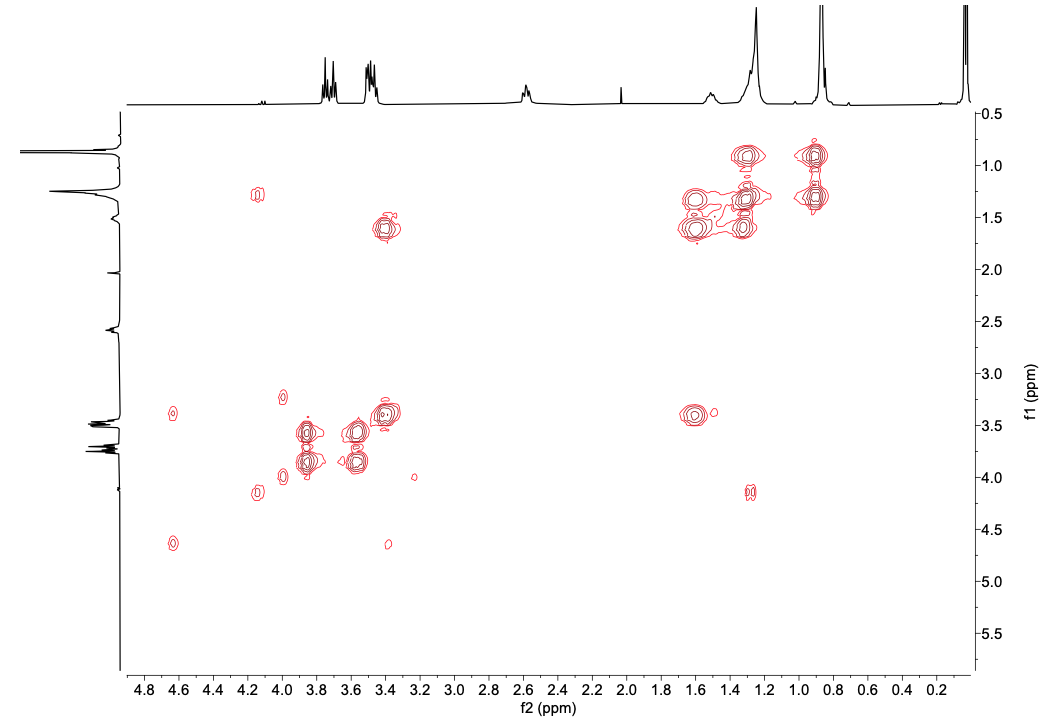
**

**Figure S7.** ^1^H NMR spectrum of **8** in CDCl_3_ at 400 MHz

**
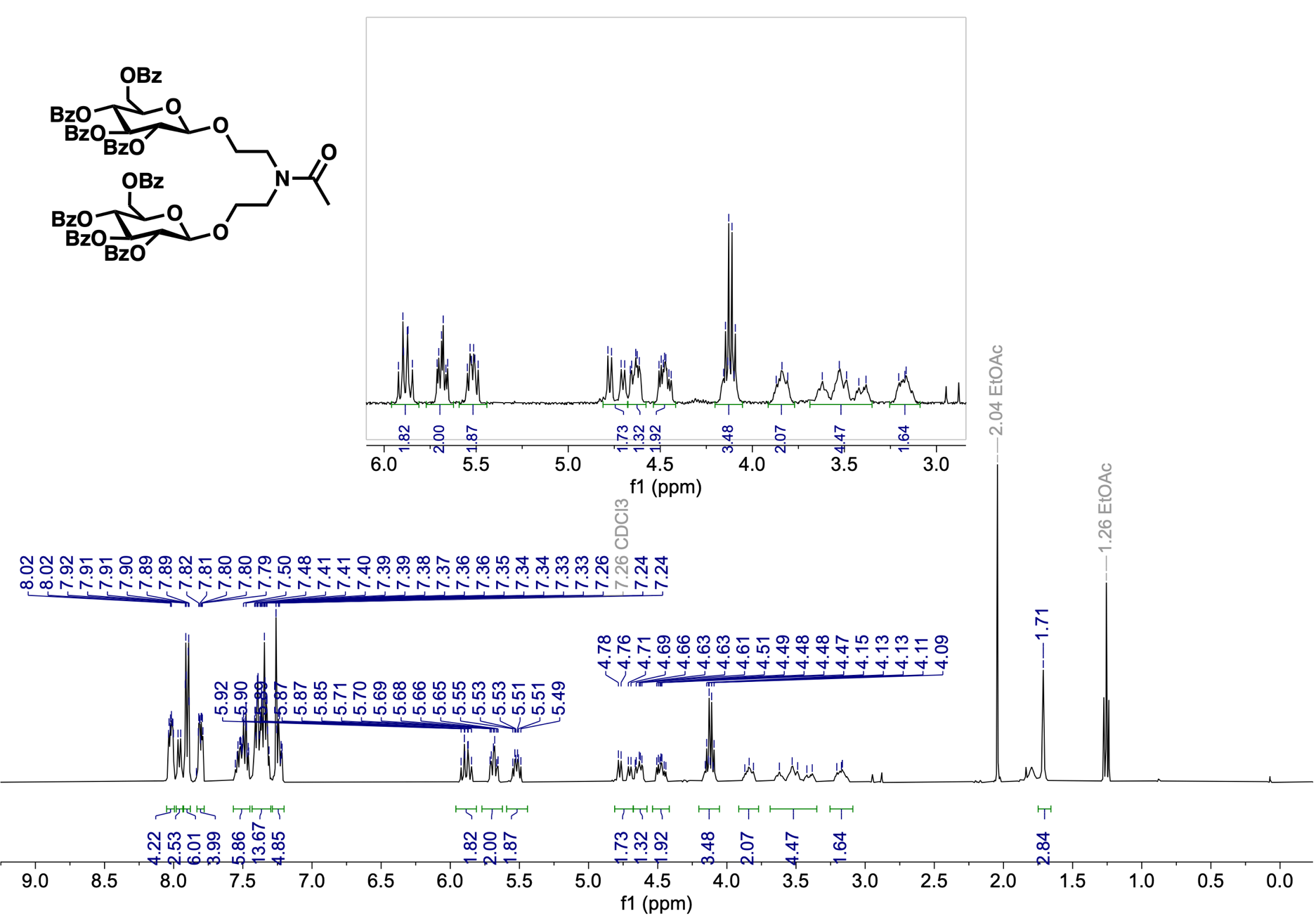
**

**Figure S8.** ^13^C NMR spectrum of **8** in CDCl_3_ at 100 MHz

**
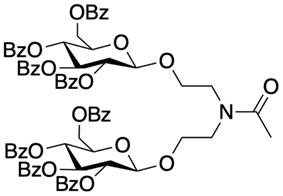
**
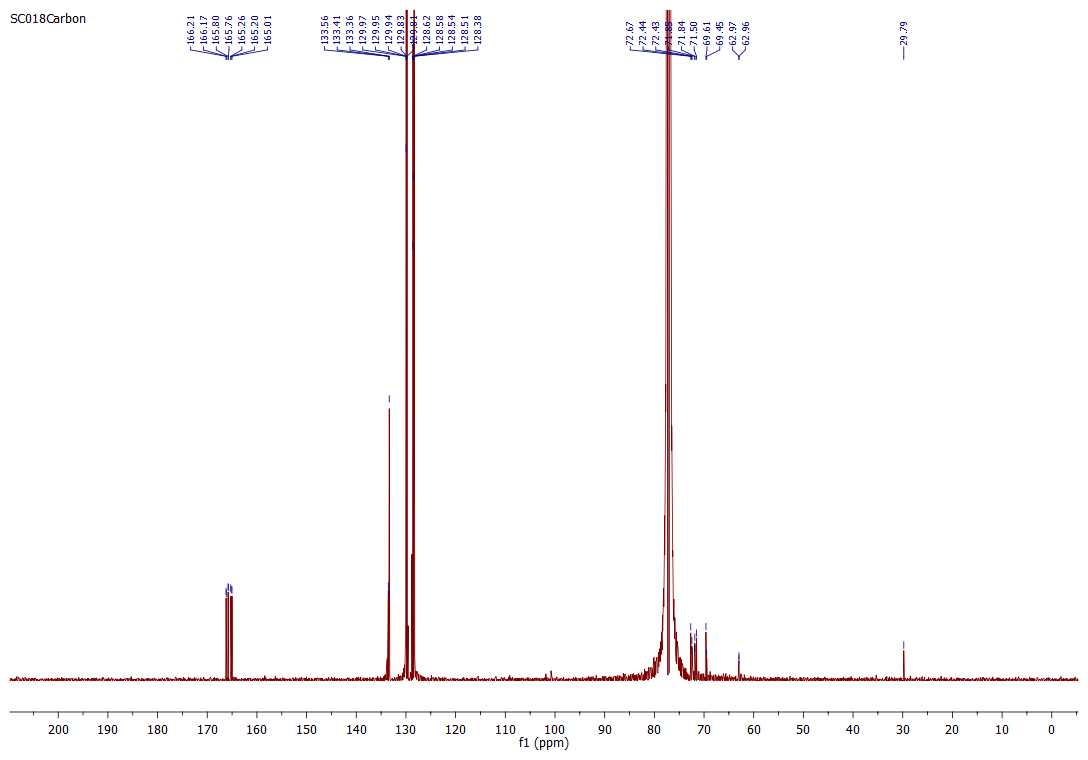


**Figure S9.** ^1^H NMR spectrum of **11a** in CDCl_3_ at 400 MHz

**
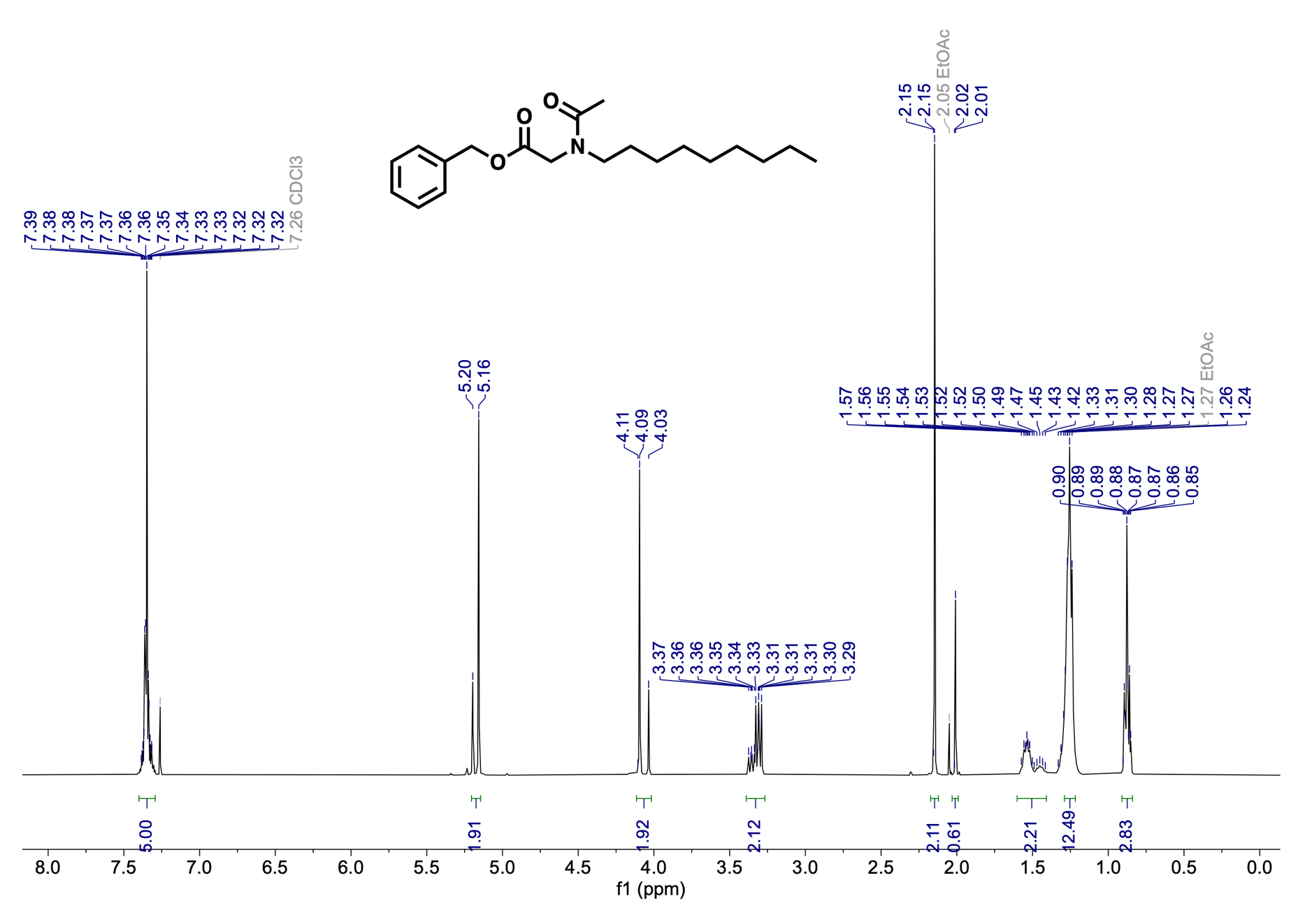
**

**Fig****ure. S10.** ^13^C NMR spectrum of **11a** in CDCl_3_ at 400 MHz

**
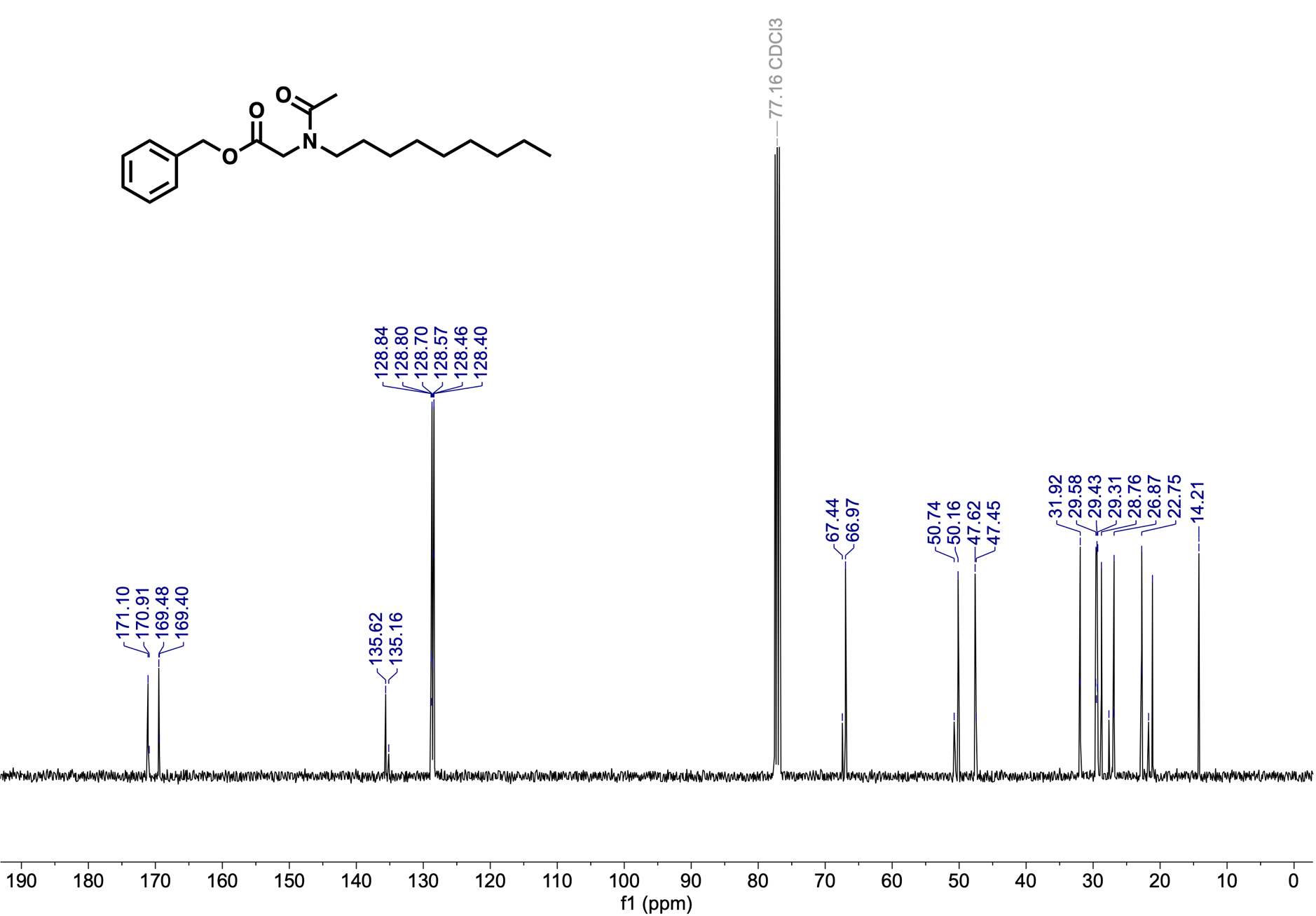
**

**Figure S11.** ^1^H NMR spectrum of **11b** in CDCl_3_ at 500 MHz

**
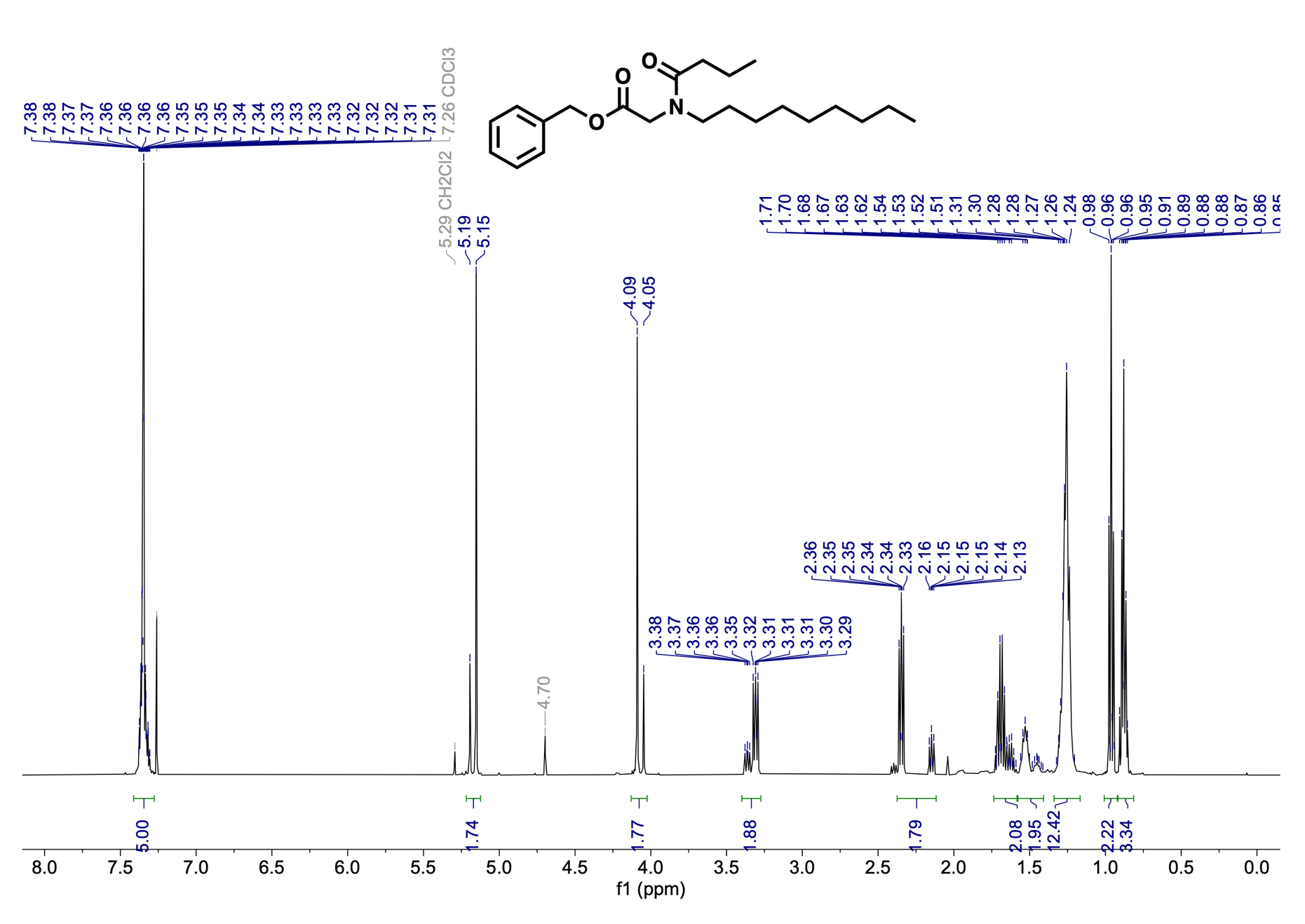
**

**Figure S12.** ^13^C NMR spectrum of **11b** in CDCl_3_ at 500 MHz

**
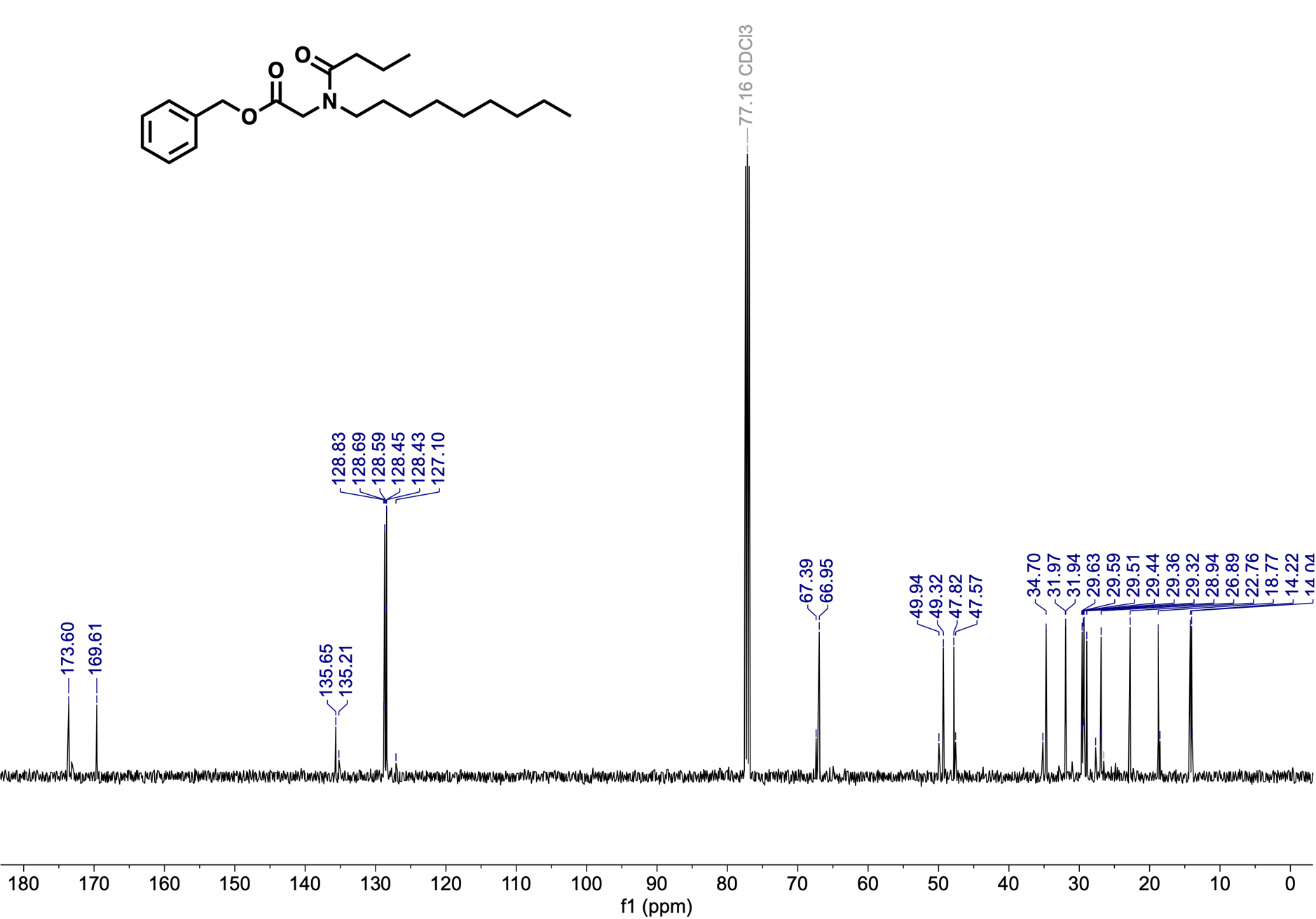
**

**Figure S13.** ^1^H NMR spectrum of **11c** in CDCl_3_ at 500 MHz

**
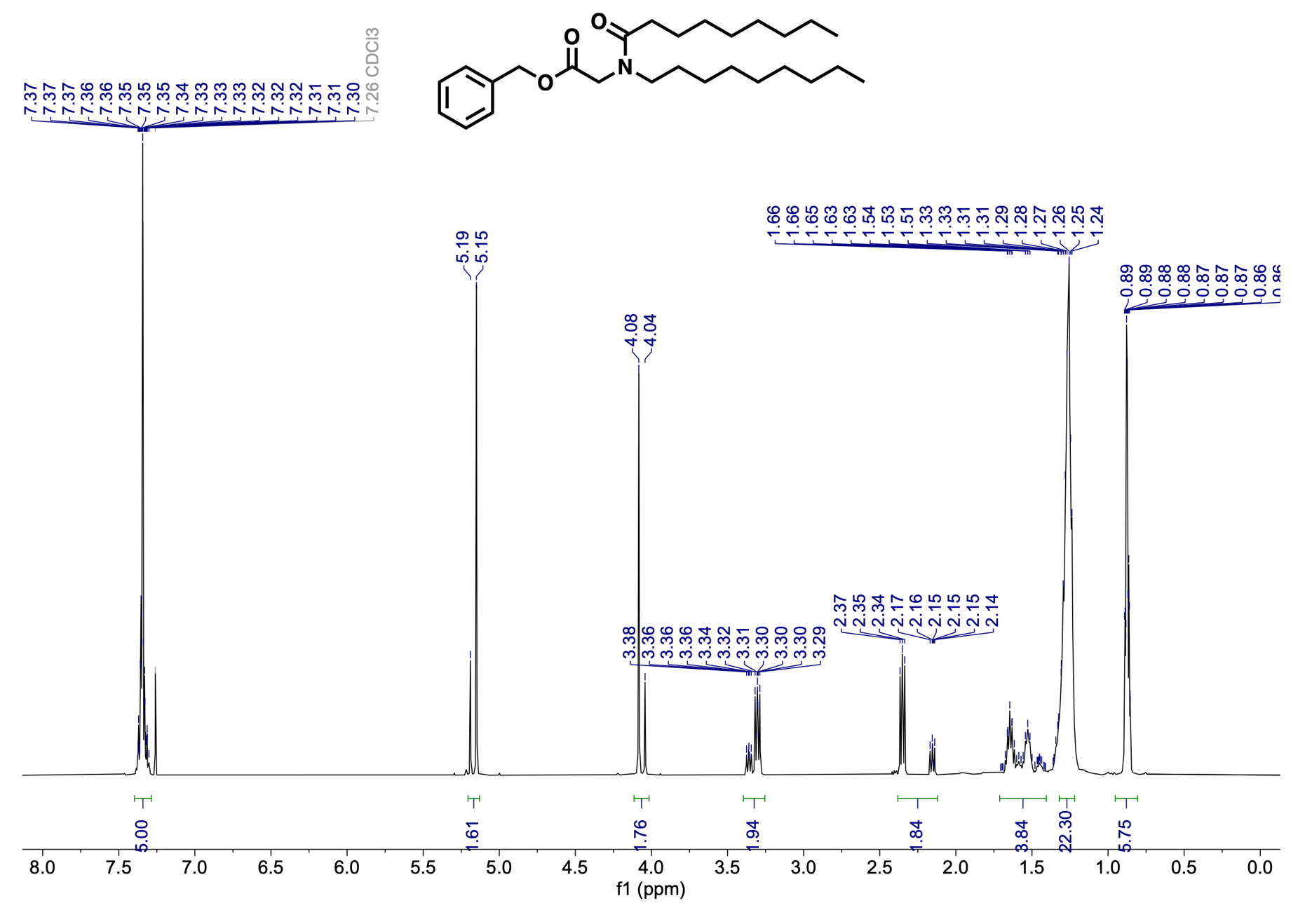
**

**FigureS14.** ^13^C NMR spectrum of **11c** in CDCl_3_ at 500 MHz

**
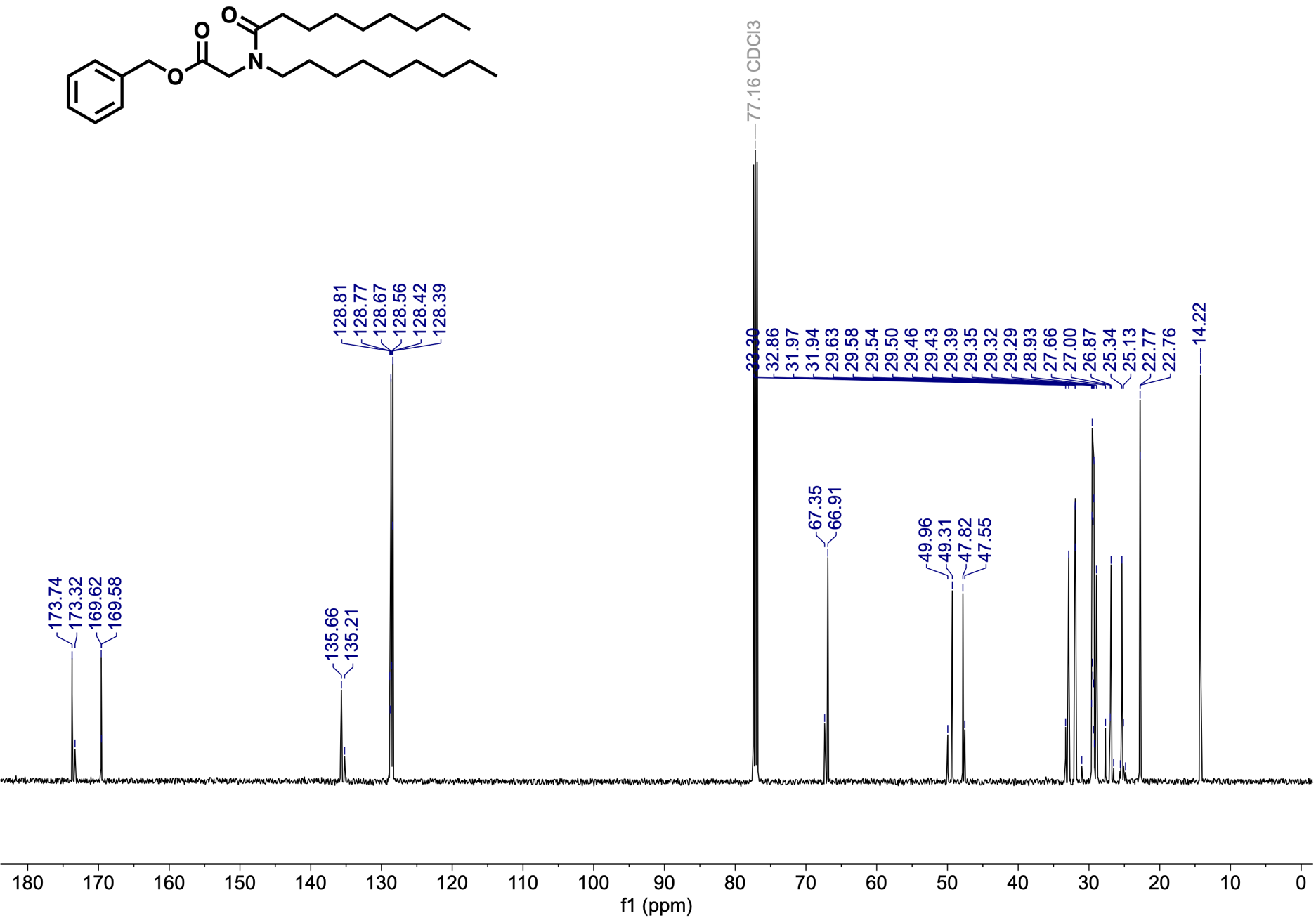
**

**Figure S15.** ^1^H NMR spectrum of **12a** in CDCl_3_ at 400 MHz

**
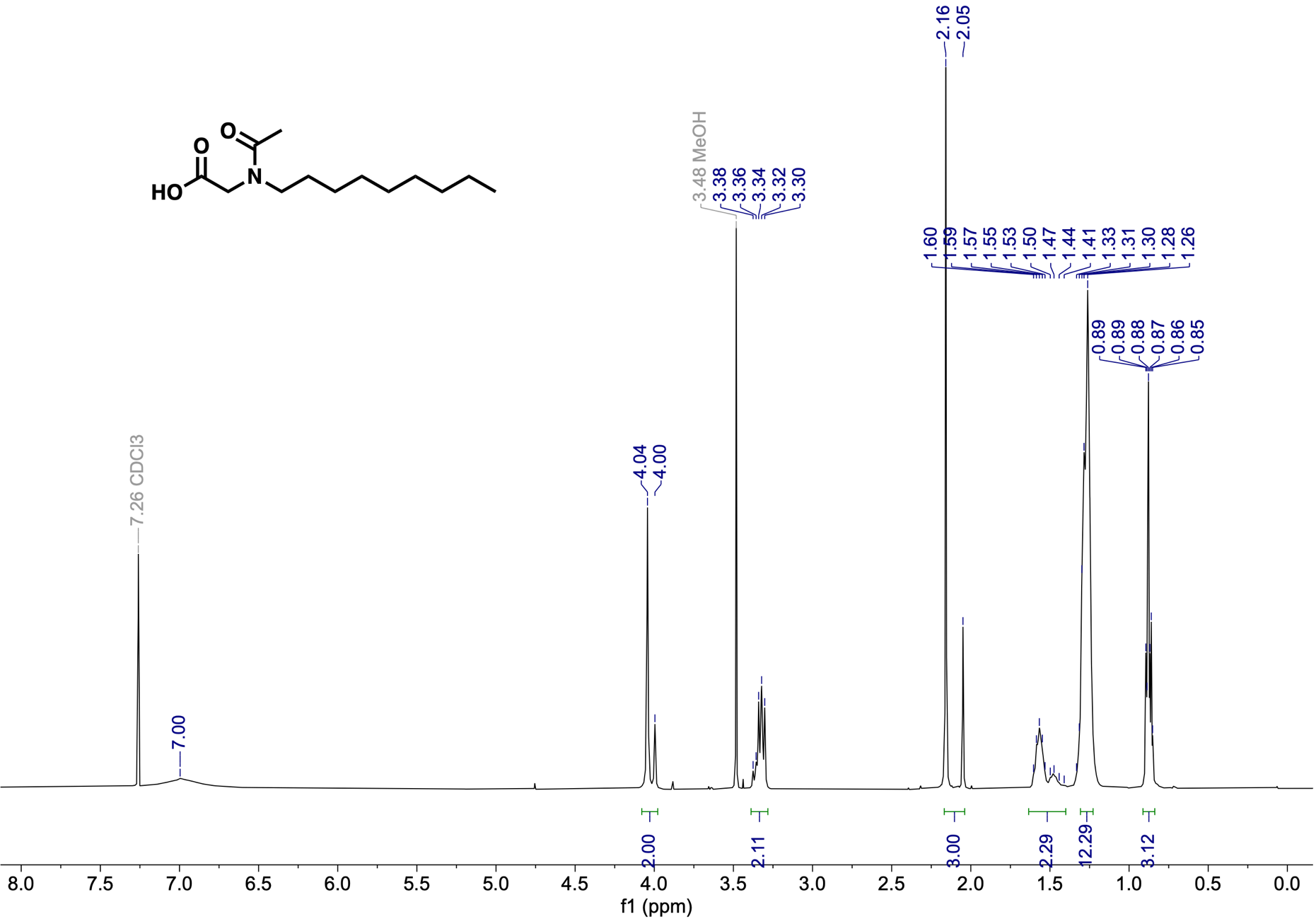
**

**Figure S16.** ^1^H NMR spectrum of **12b** in CDCl_3_ at 400 MHz

**
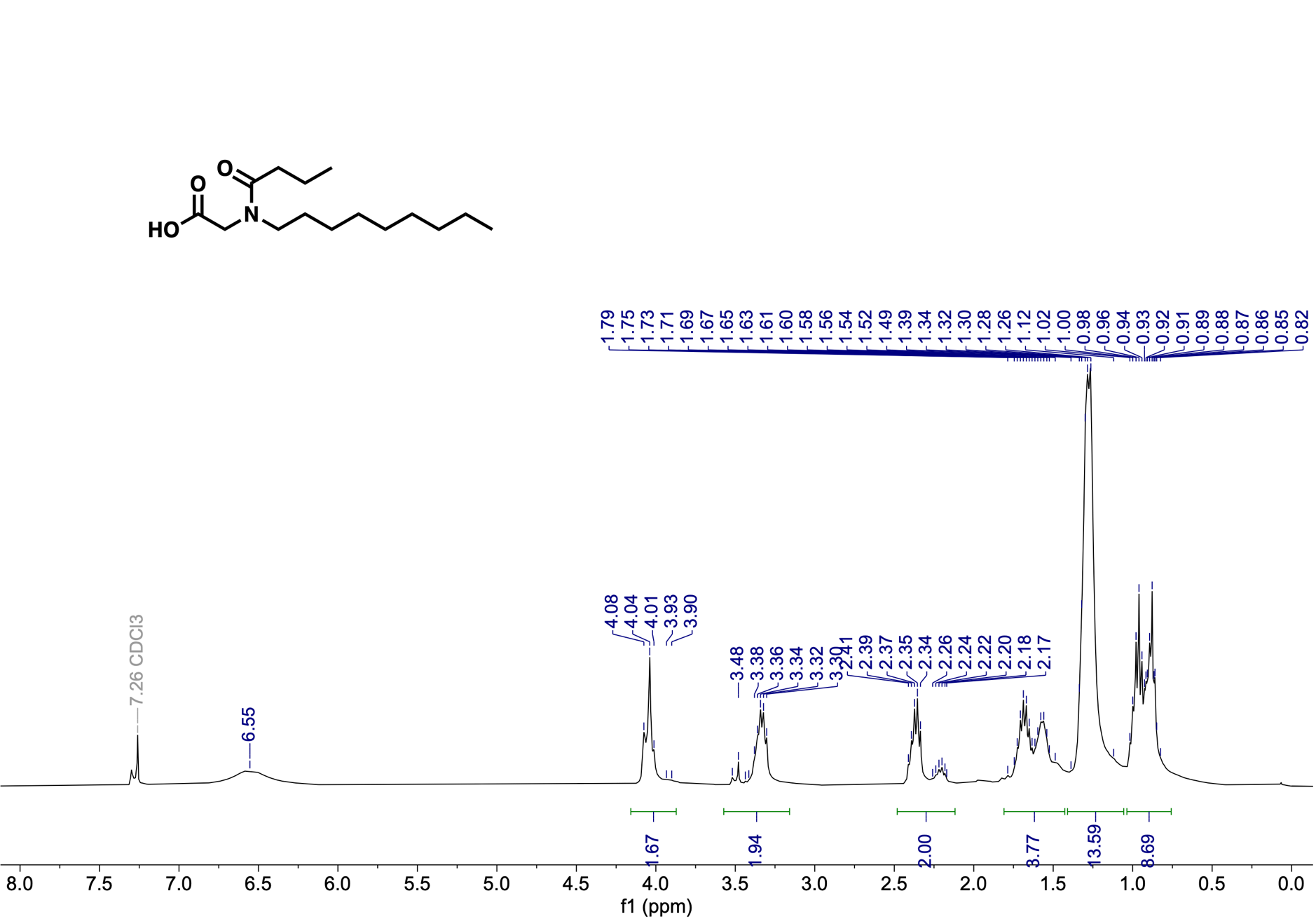
**

**Figure S17.** ^1^H NMR spectrum of **12c** in CDCl_3_ at 400 MHz

**
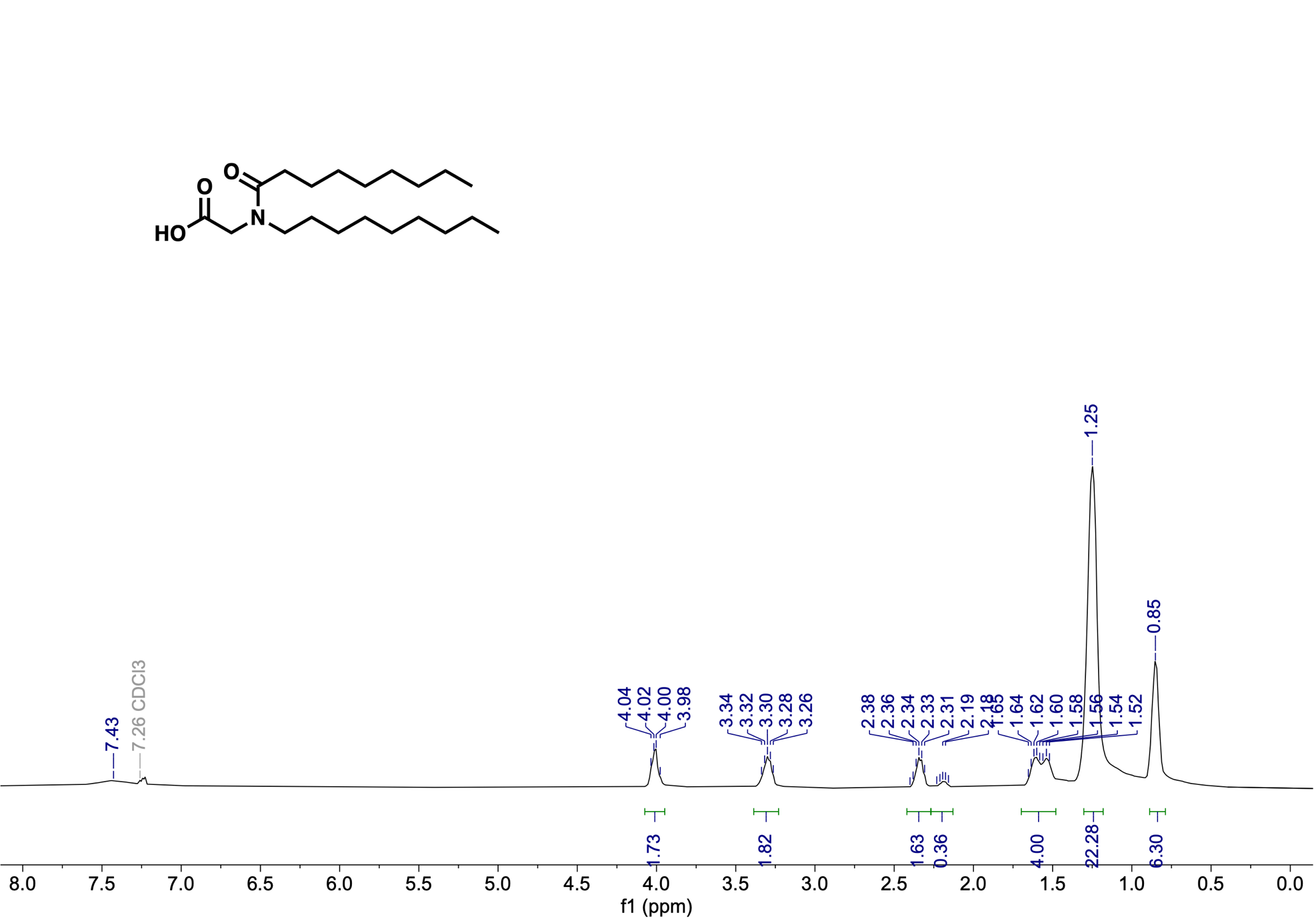
**

**Figure S18.** ^1^H NMR spectrum of **13** in CDCl_3_ at 400 MHz

**
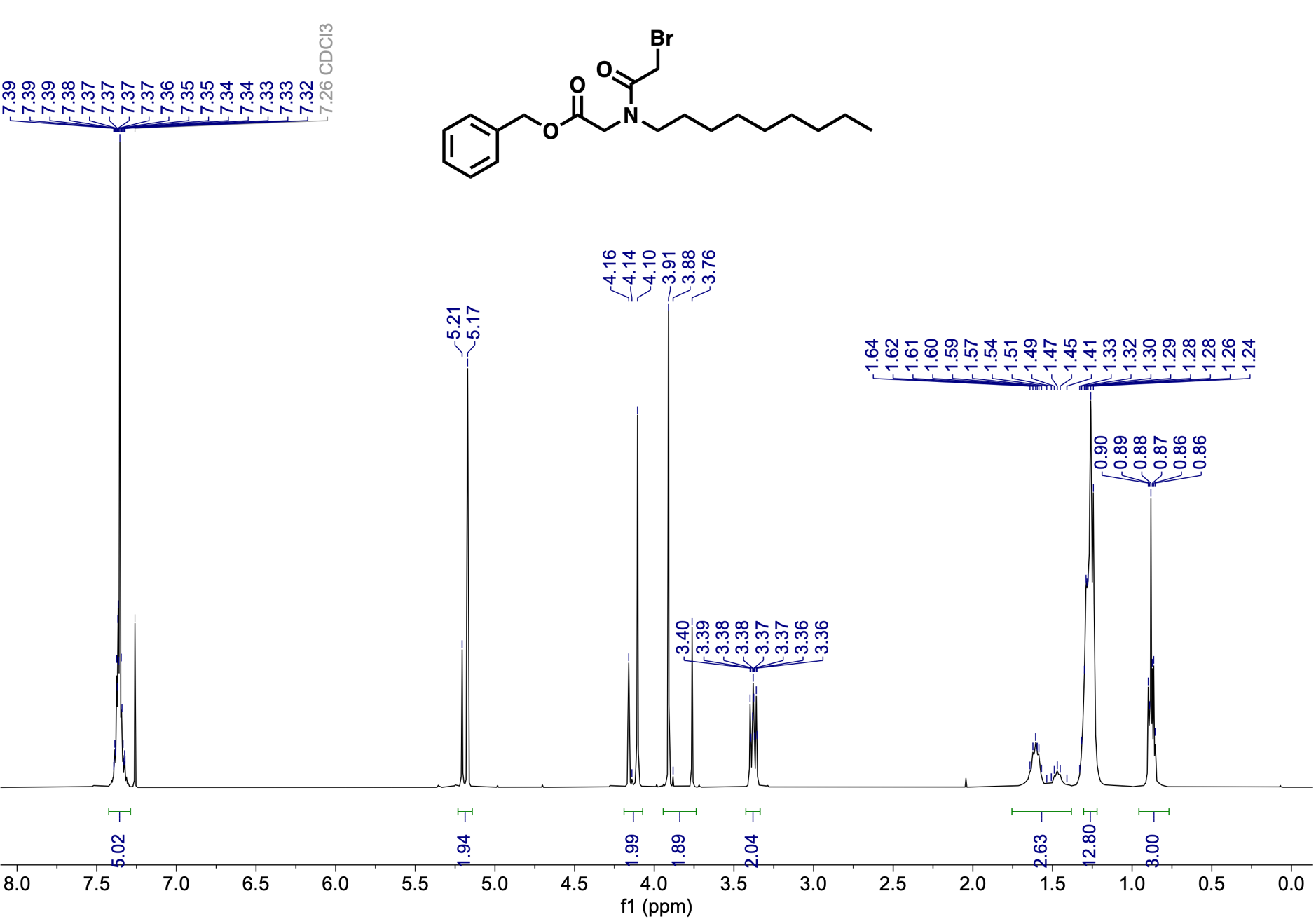
**

**Figure S19.** ^13^C NMR spectrum of **13** in CDCl_3_ at 100 MHz

**
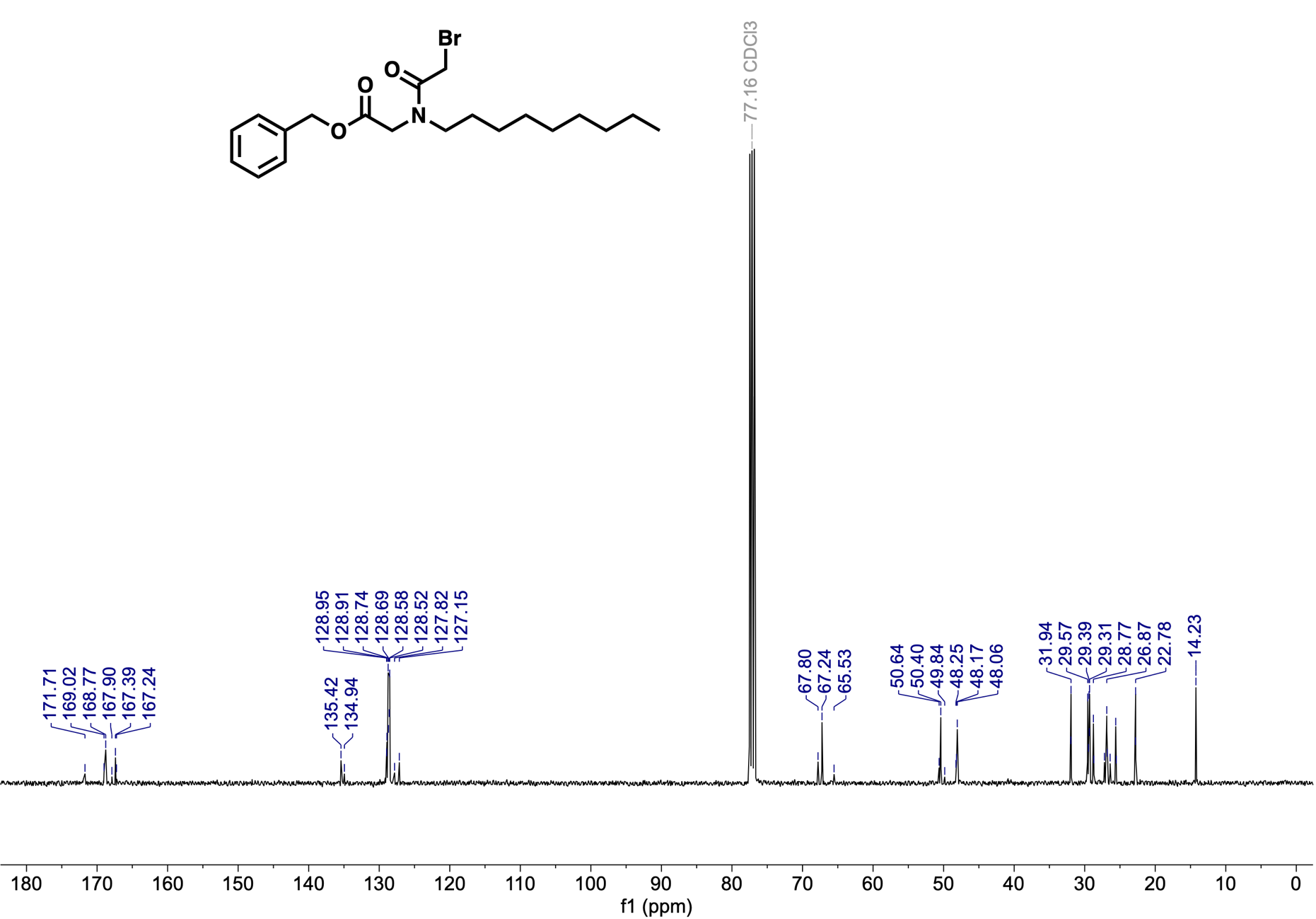
**

**Figure S20.** ^1^H NMR spectrum of **16** in CDCl_3_ at 500 MHz

**
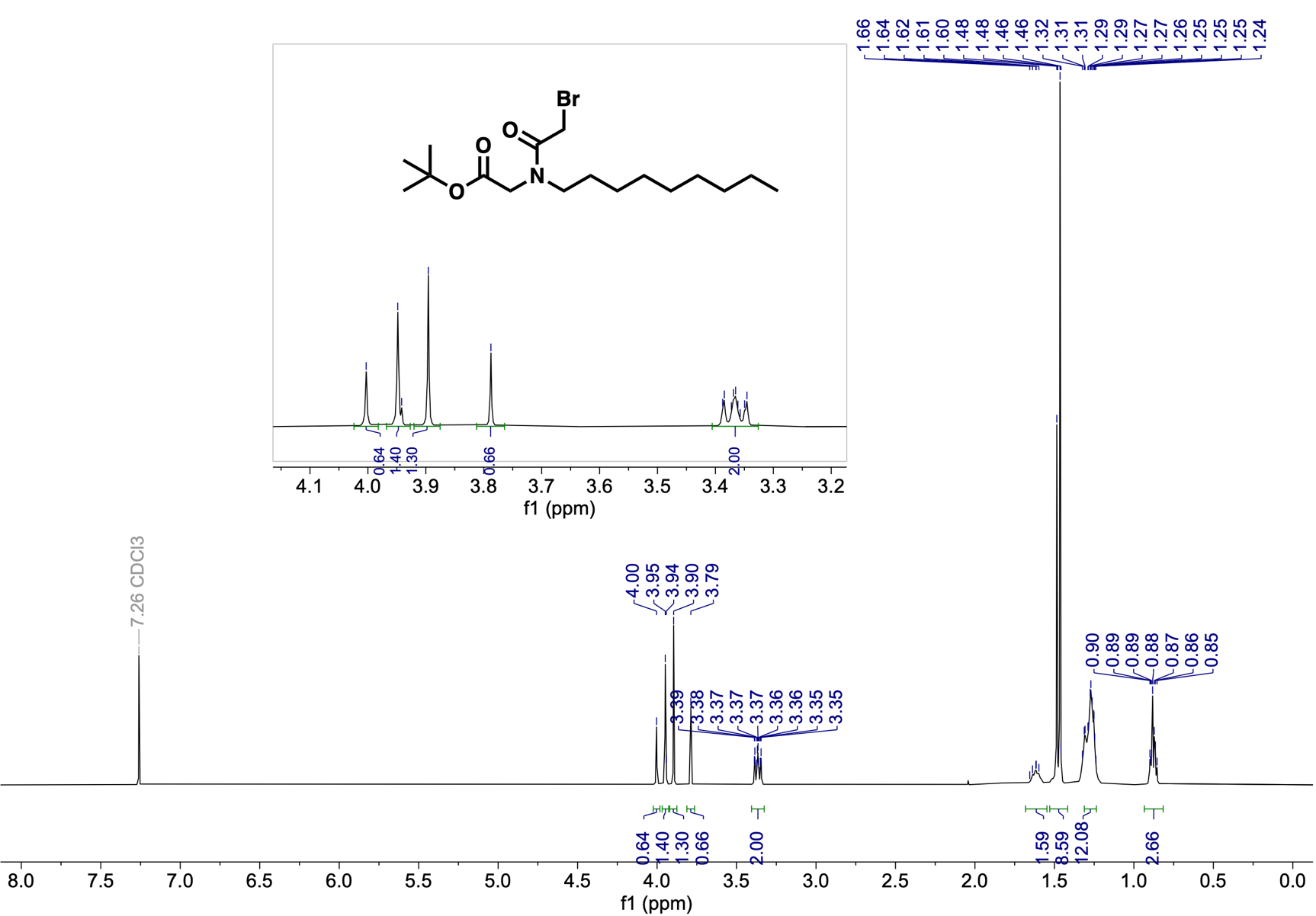
**

**Figure S21.** ^1^H NMR spectrum of **14** in CDCl_3_ at 500 MHz

**
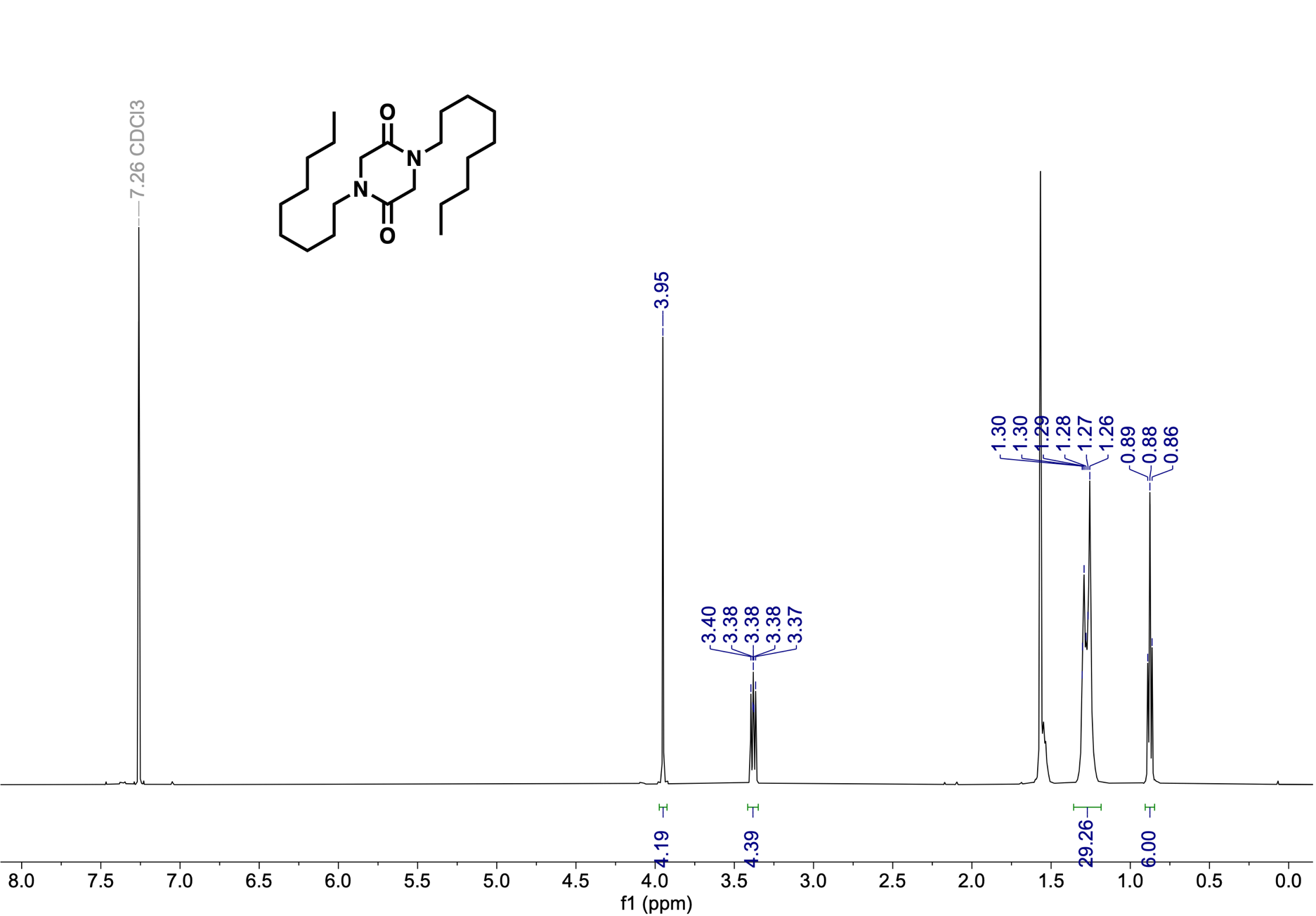
**

**Figure S22.** ^13^C NMR spectrum of **14** in CDCl_3_ at 100 MHz


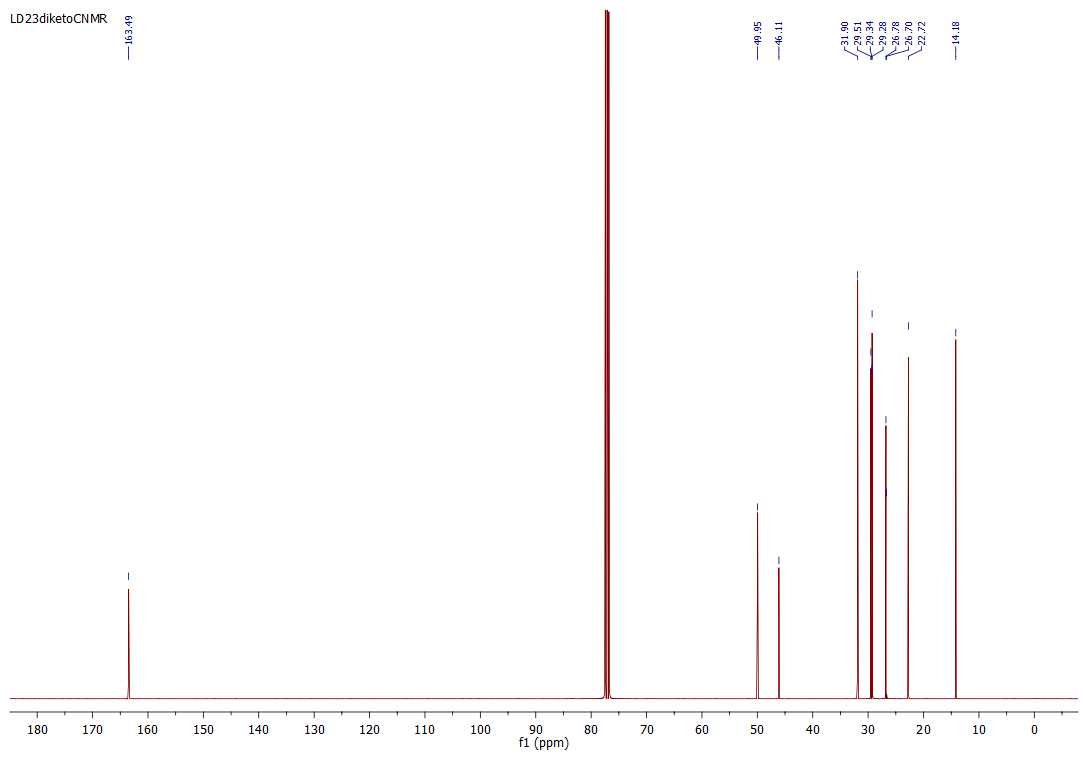


**Figure S23.** ^13^C NMR spectrum of **16** in CDCl_3_ at 125 MHz

**
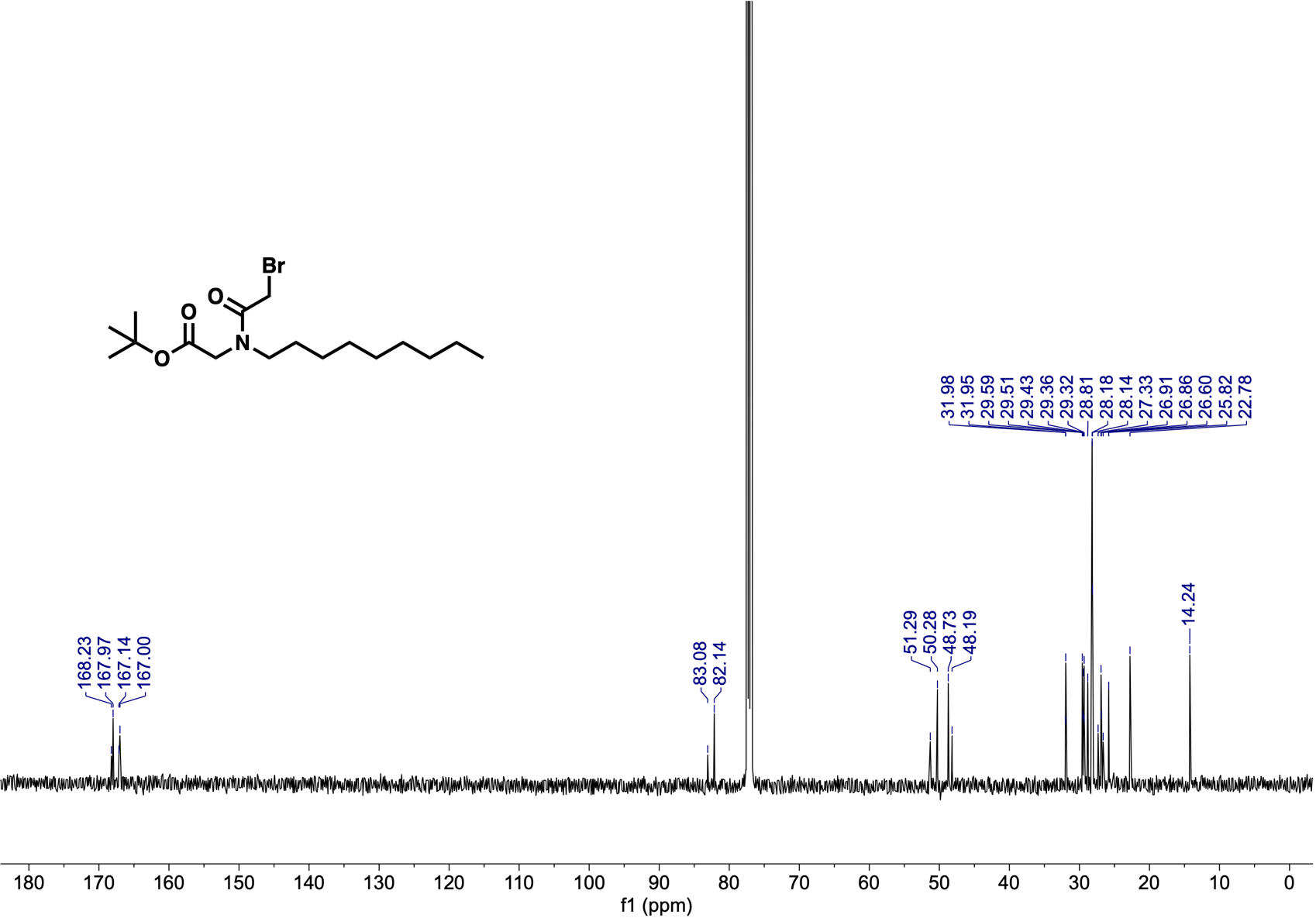
**

**Figure S24.** ^1^H NMR spectrum of **17** in CDCl_3_ at 500 MHz

**
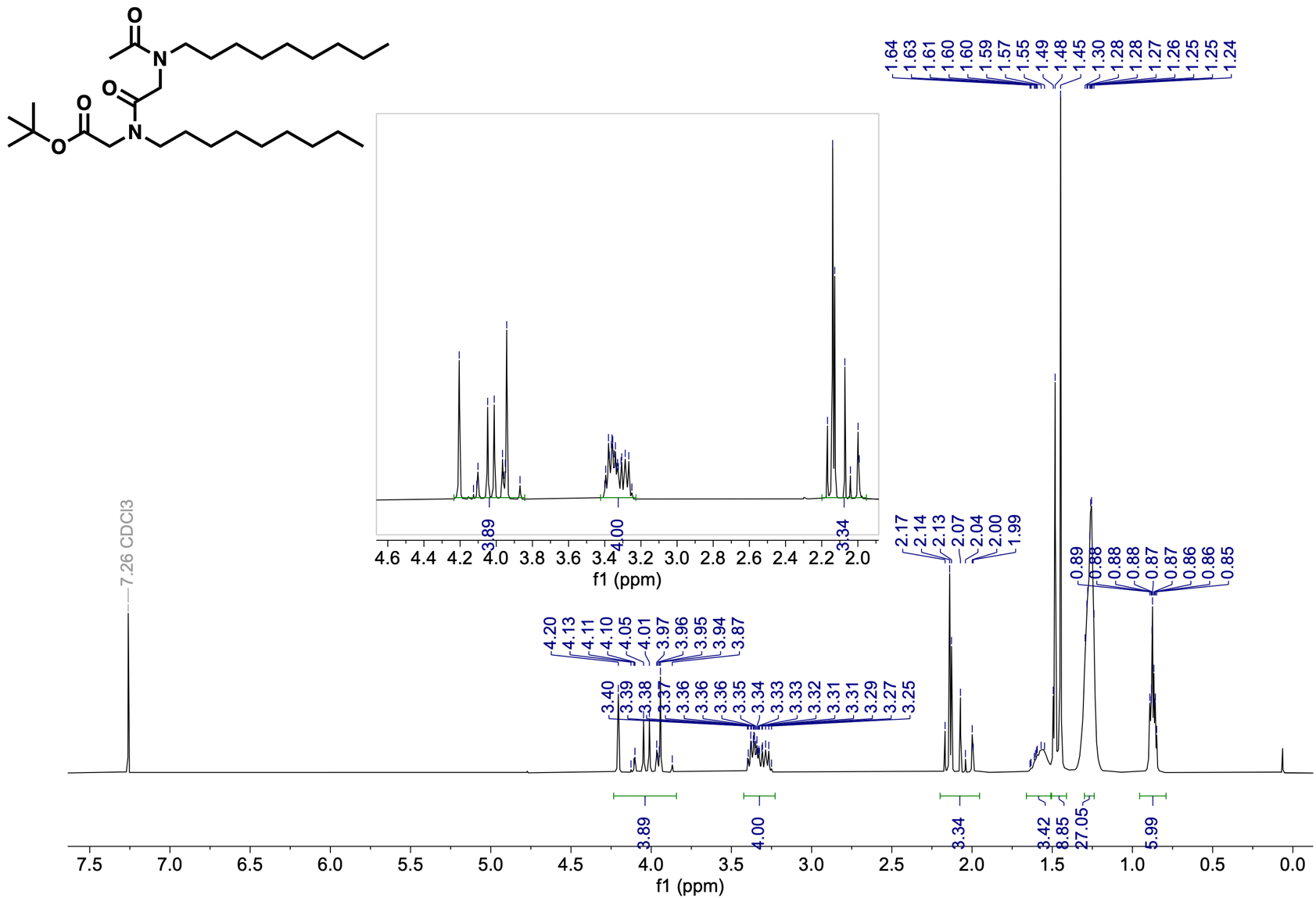
**

**Figure S25.
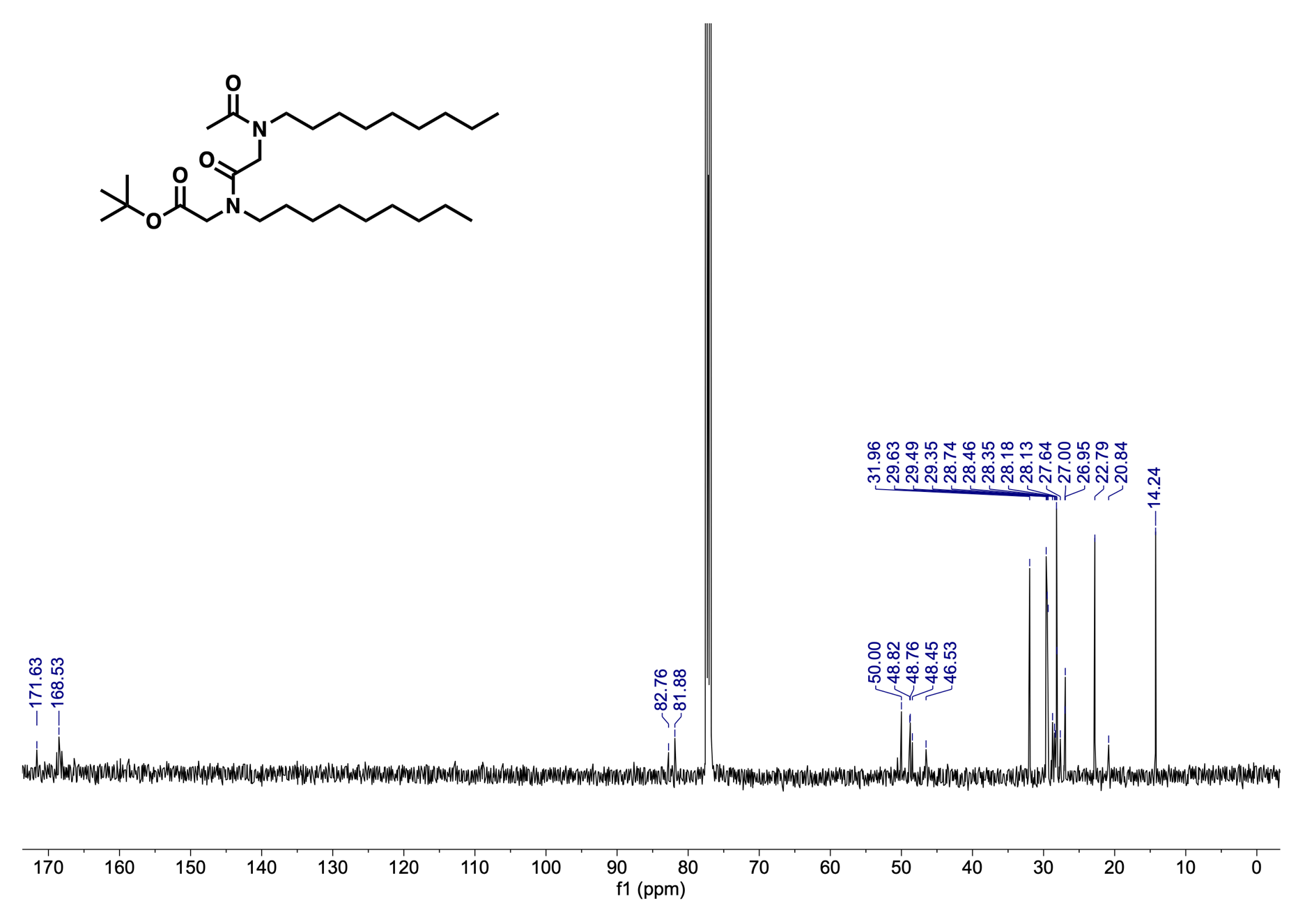
** ^13^C NMR spectrum of **17** in CDCl_3_ at 125 MHz

**Figure S26.** ^1^H NMR spectrum of **19** in CDCl_3_ at 500 MHz

**
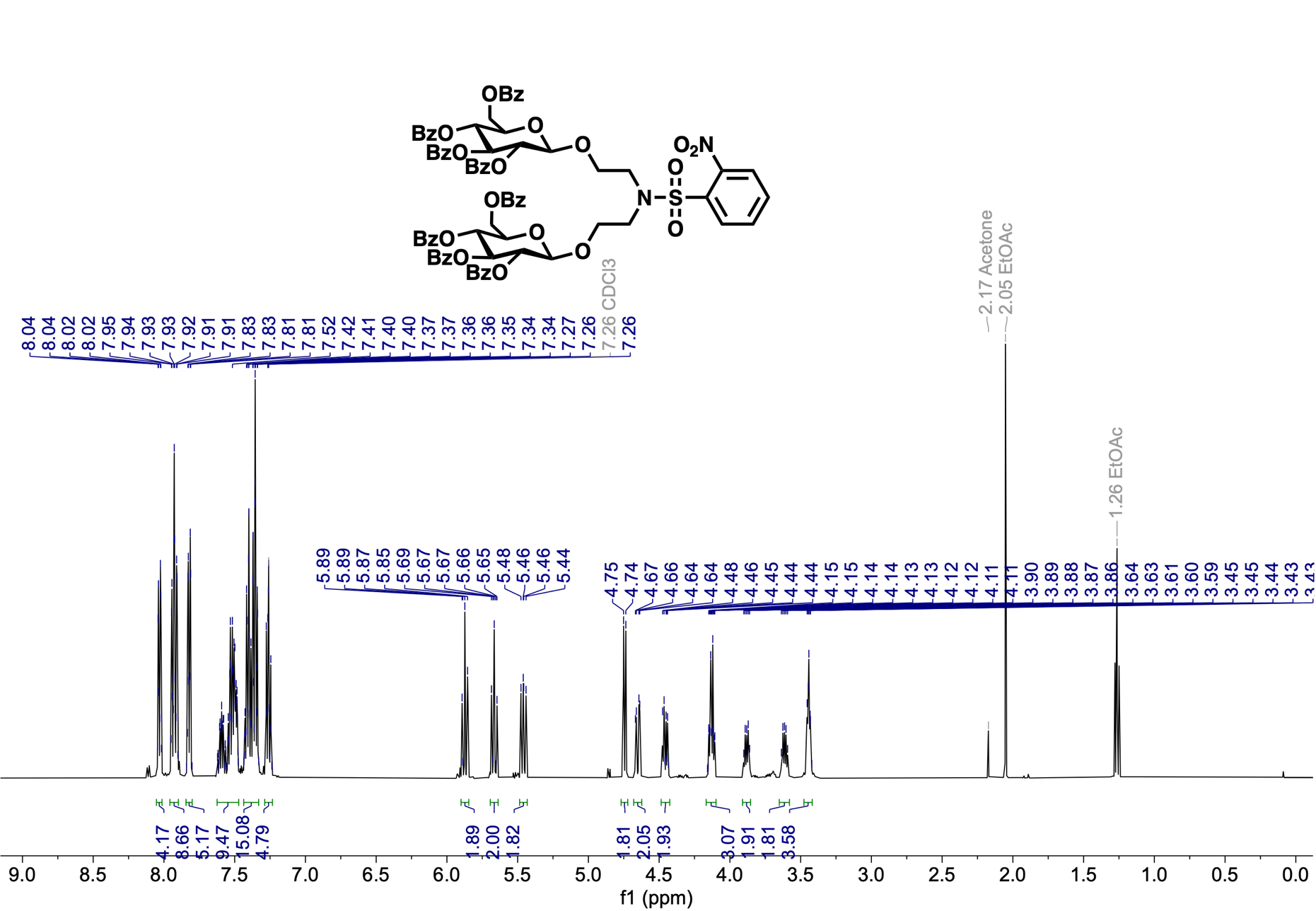
**

**Figure S27.** Enlarged view of a selected region of the ^1^H NMR spectrum of **19** in CDCl_3_ at 500 MHz

**
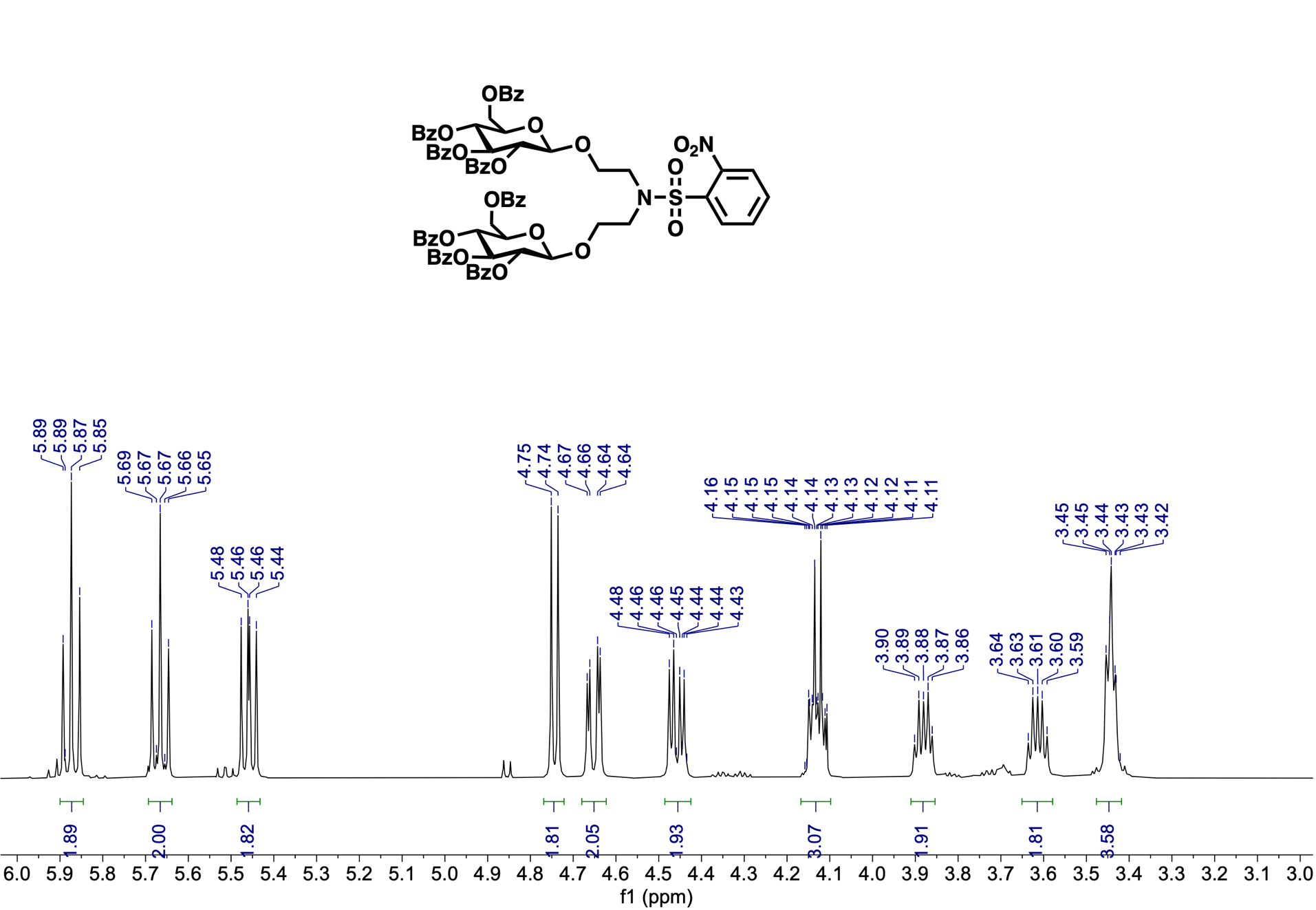
**

**Figure S28.** ^13^C NMR spectrum of **19** in CDCl_3_ at 125 MHz

**
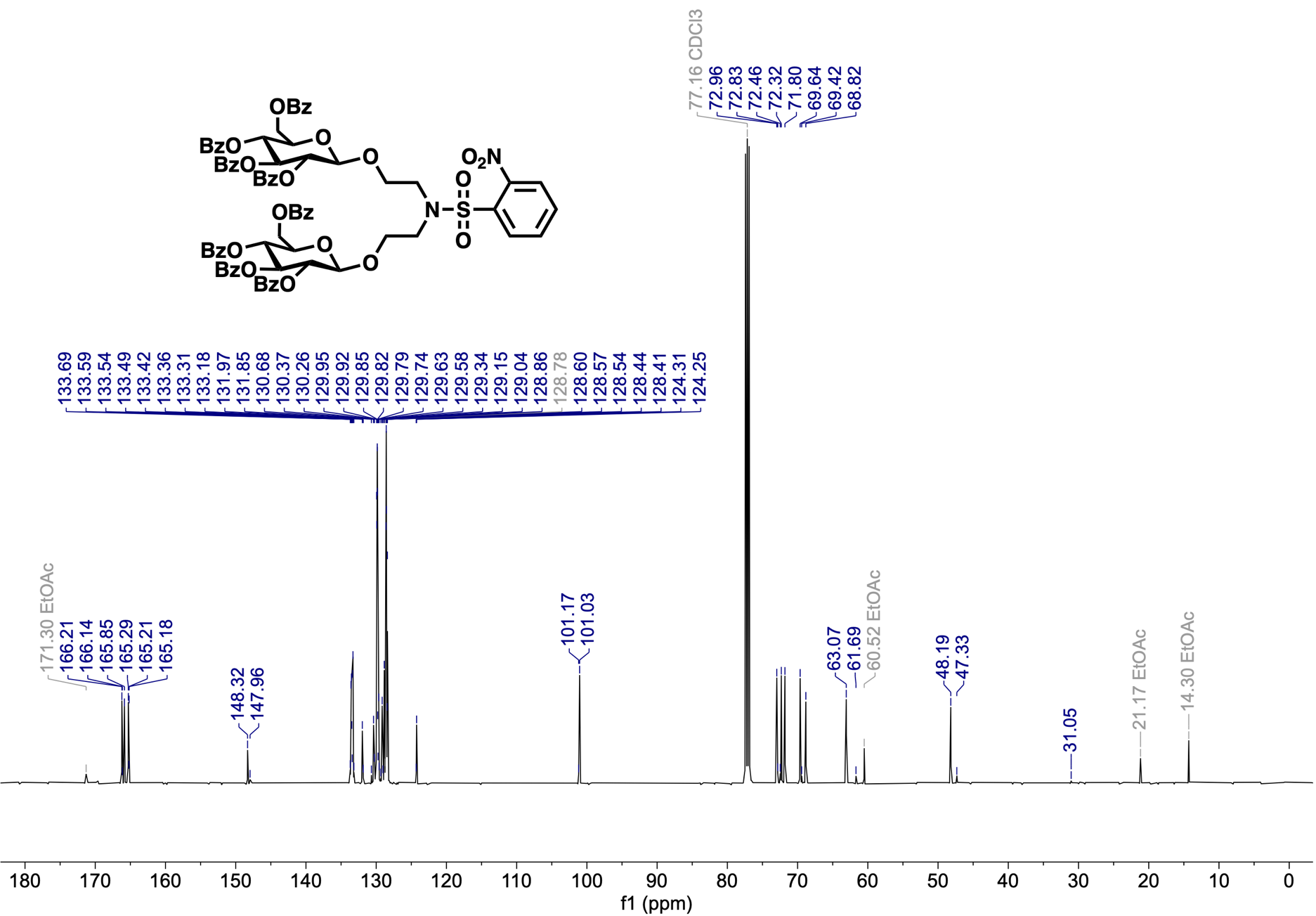
**

**Figure S29.** ^1^H NMR spectrum of **20** in CDCl_3_ at 400 MHz

**
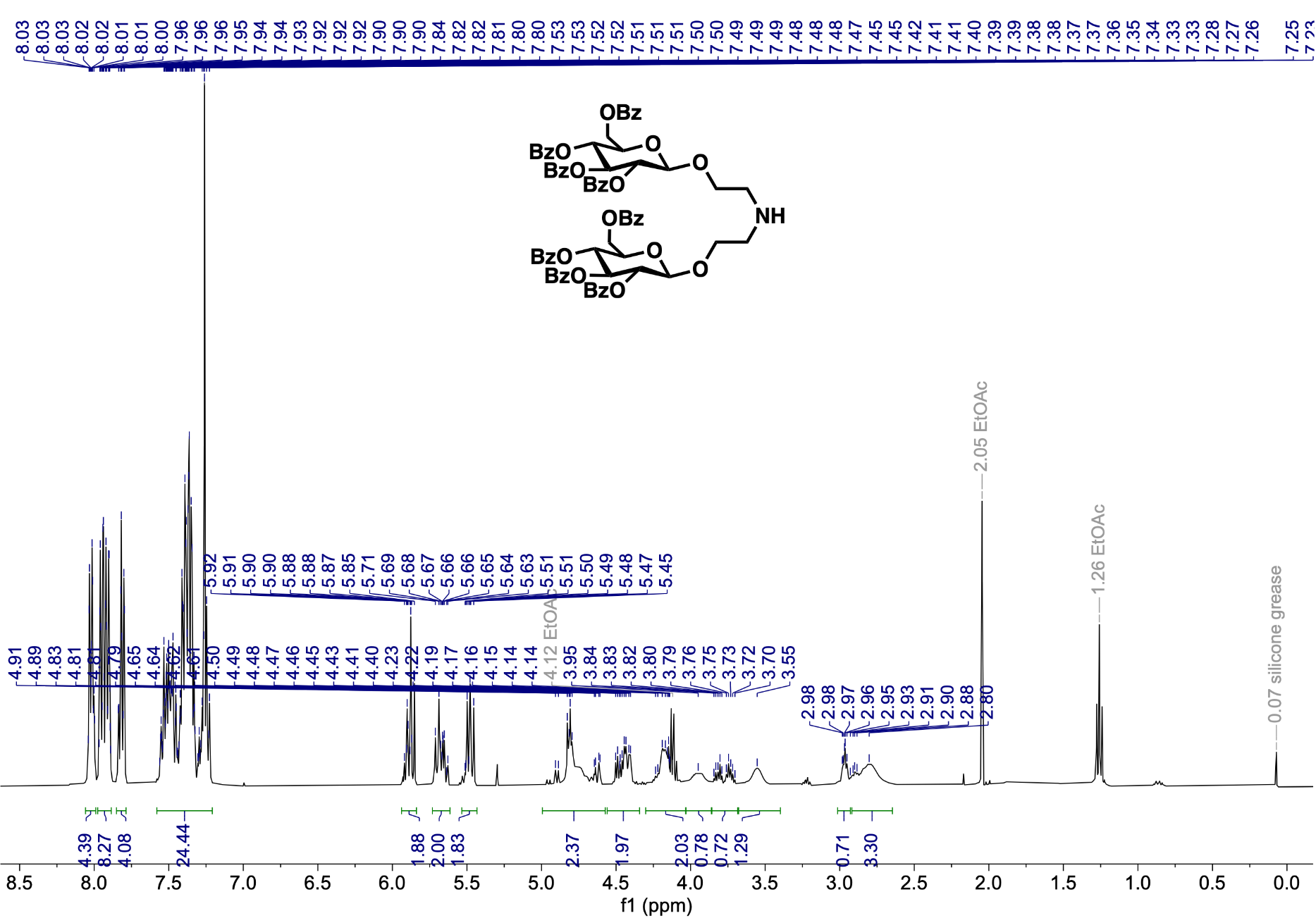
**

**Figure S30.** Enlarged view of a selected region of the ^1^H NMR spectrum of **20** in CDCl_3_ at 400 MHz

**
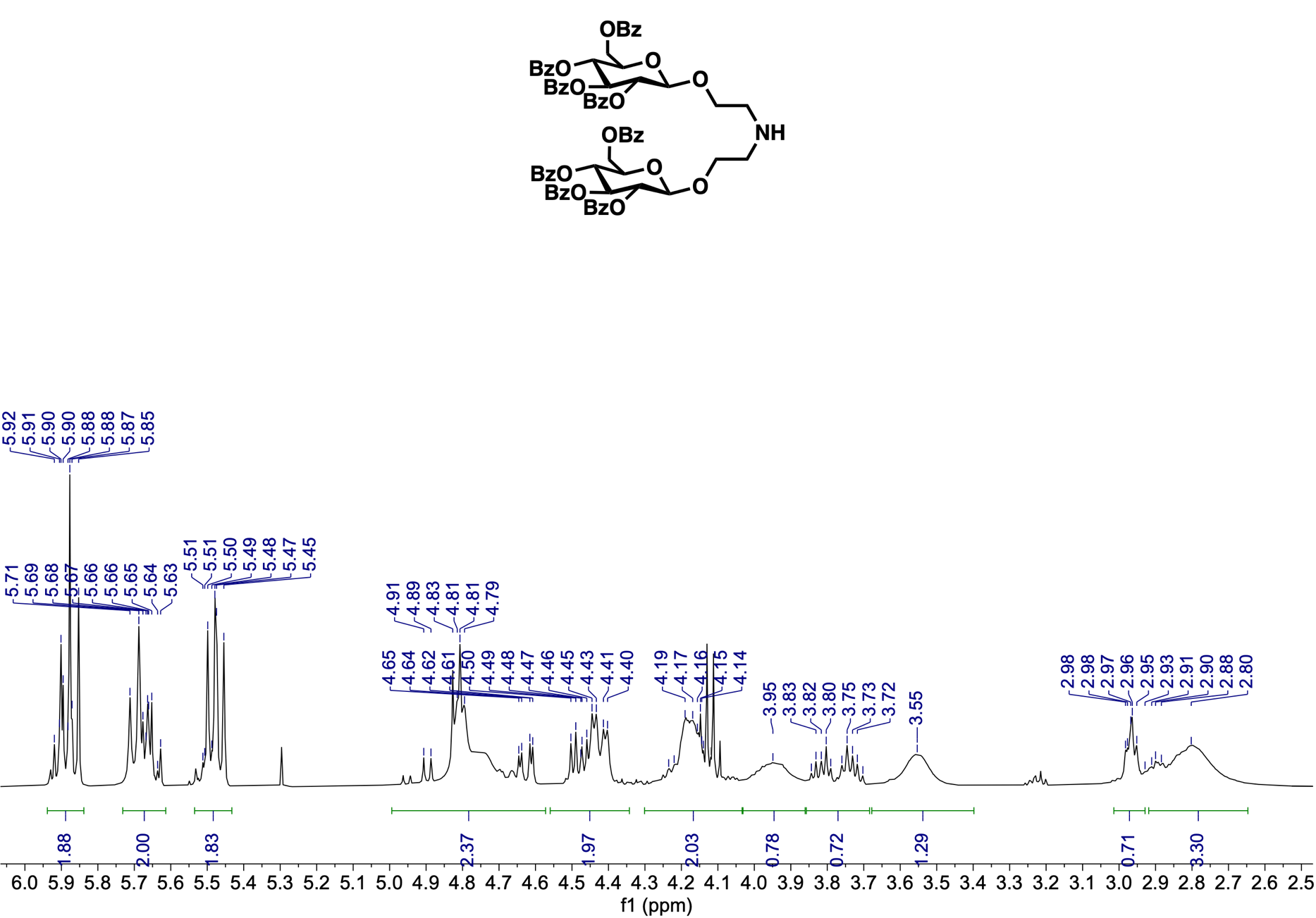
**

**Figure S31.** ^1^H NMR spectrum of **9** in CDCl_3_ at 400 MHz

**
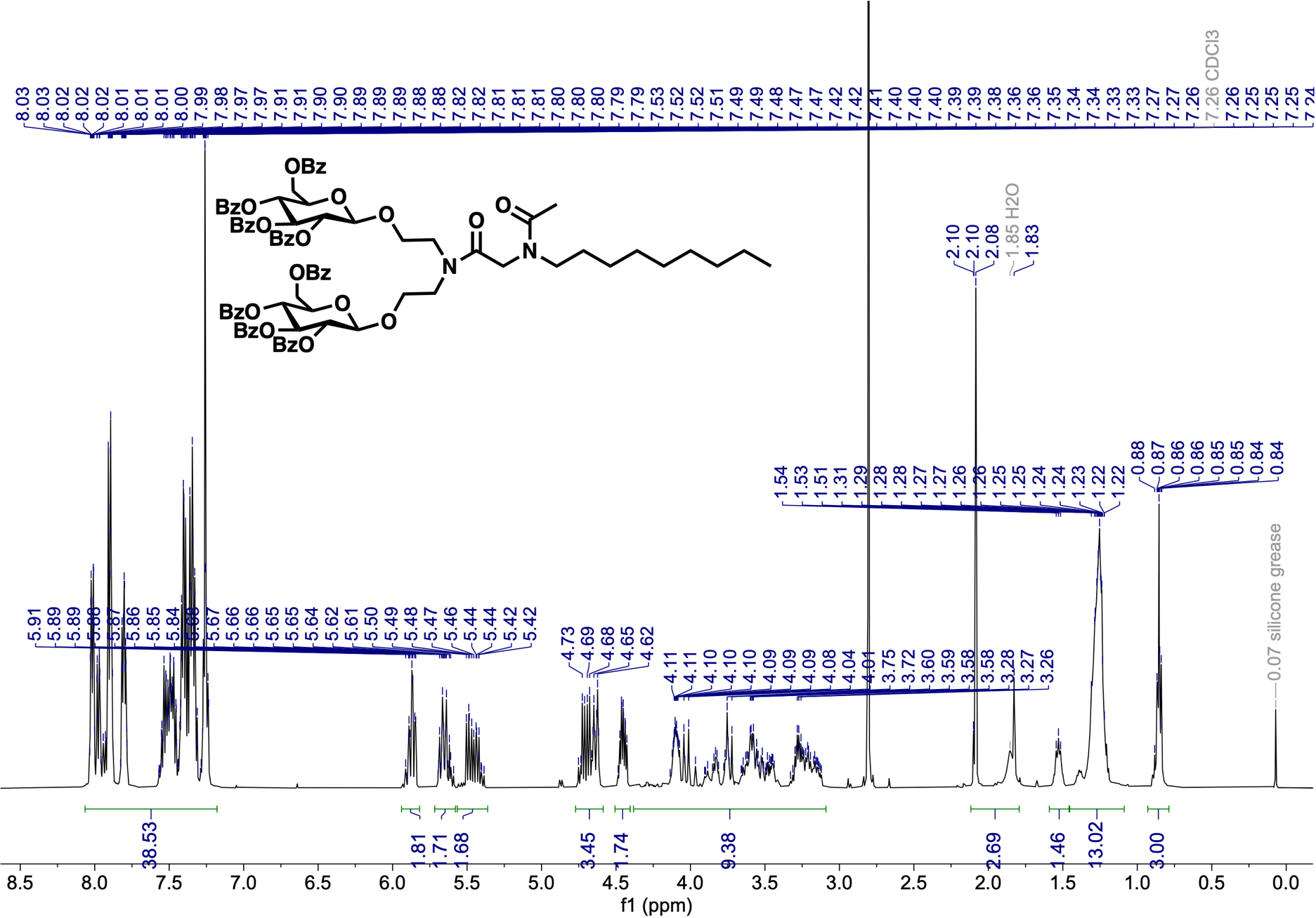
**

**Figure S32.** Enlarged view of a selected region of the ^1^H NMR spectrum of **9** in CDCl_3_ at 400 MHz

**
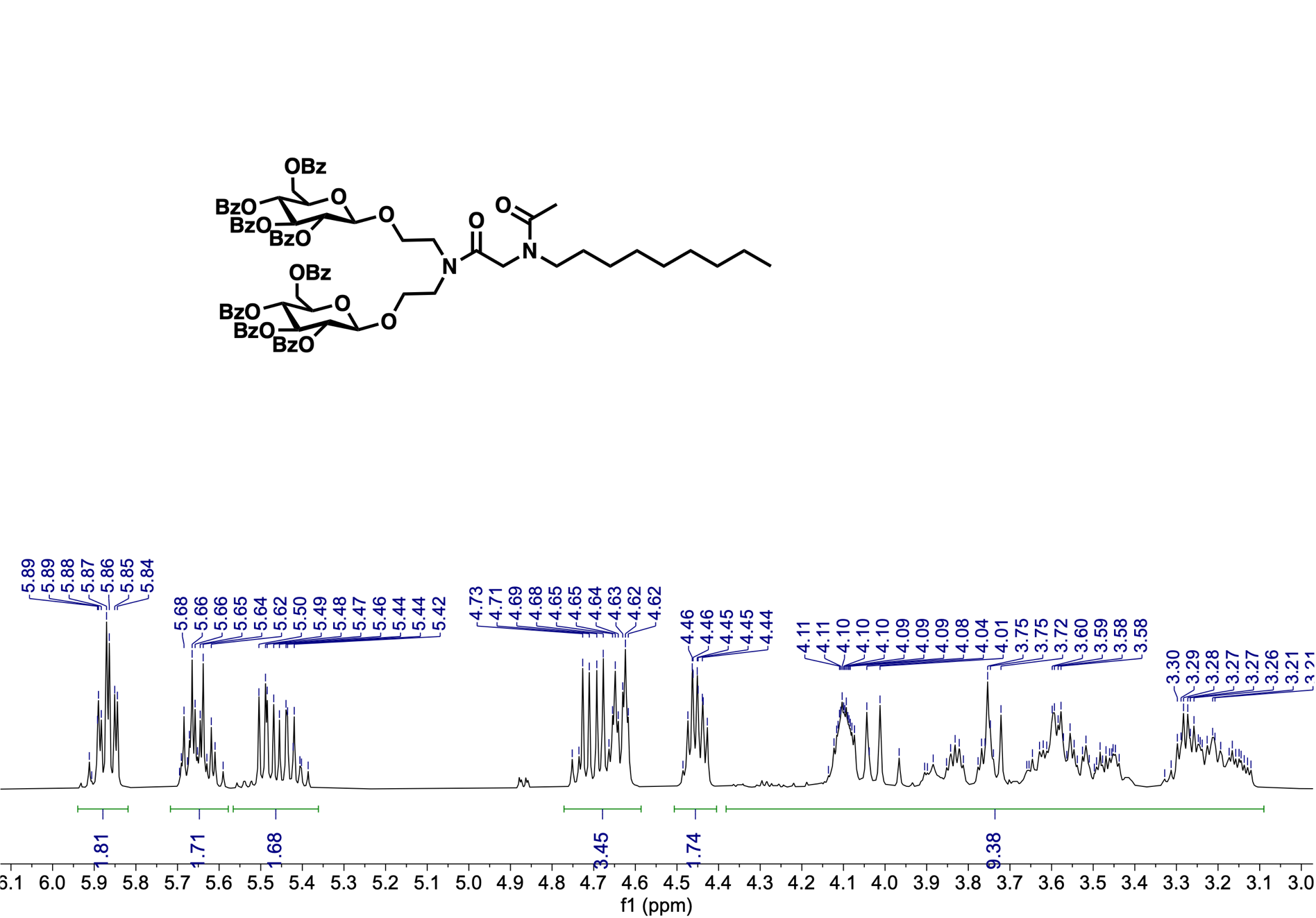
**

**Figure S33.** ^13^C NMR spectrum of **9** in CDCl_3_ at 100 MHz

**
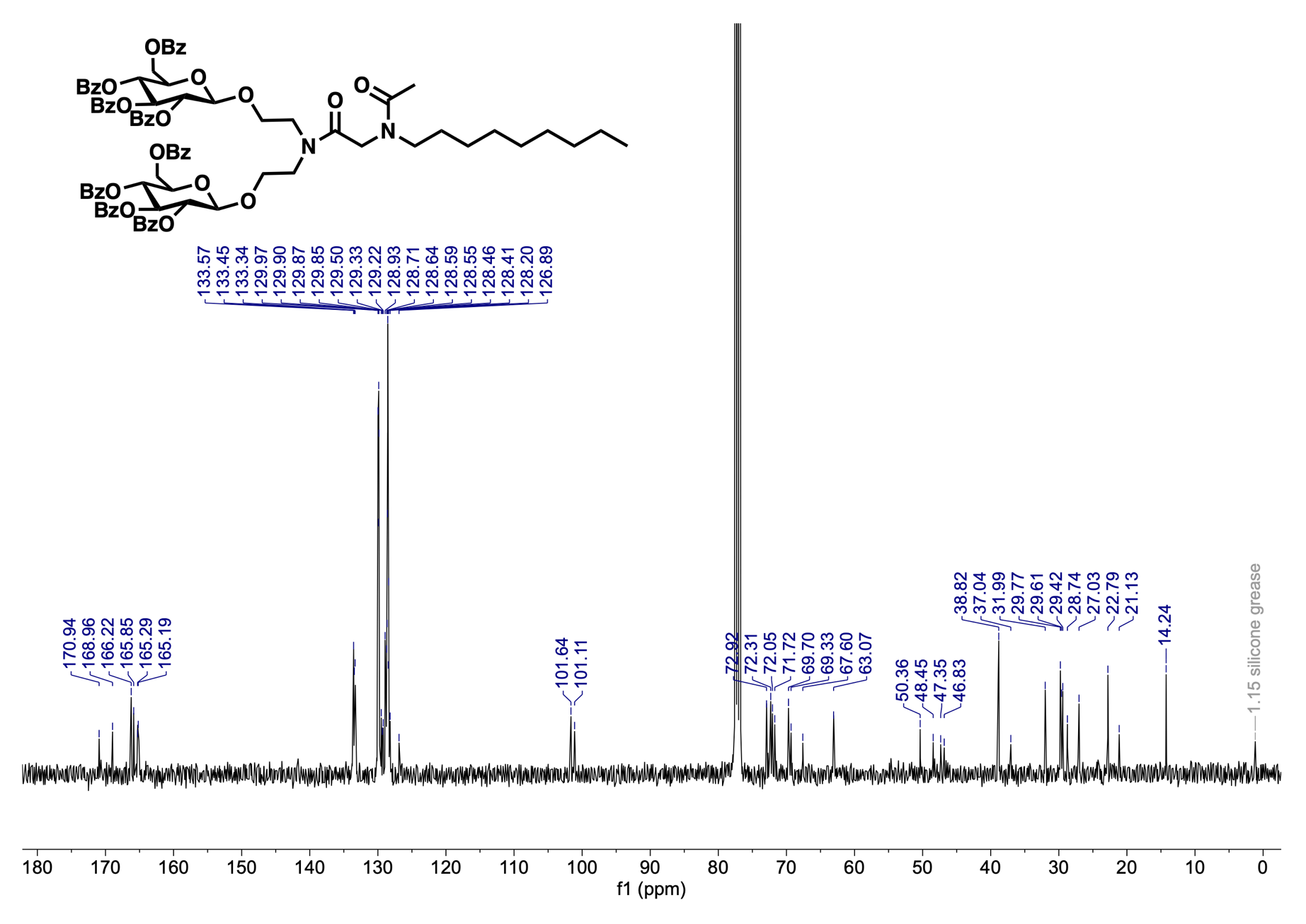
**

**Figure S34.** ^1^H NMR spectrum of **21a** in CDCl_3_ at 500 MHz

**
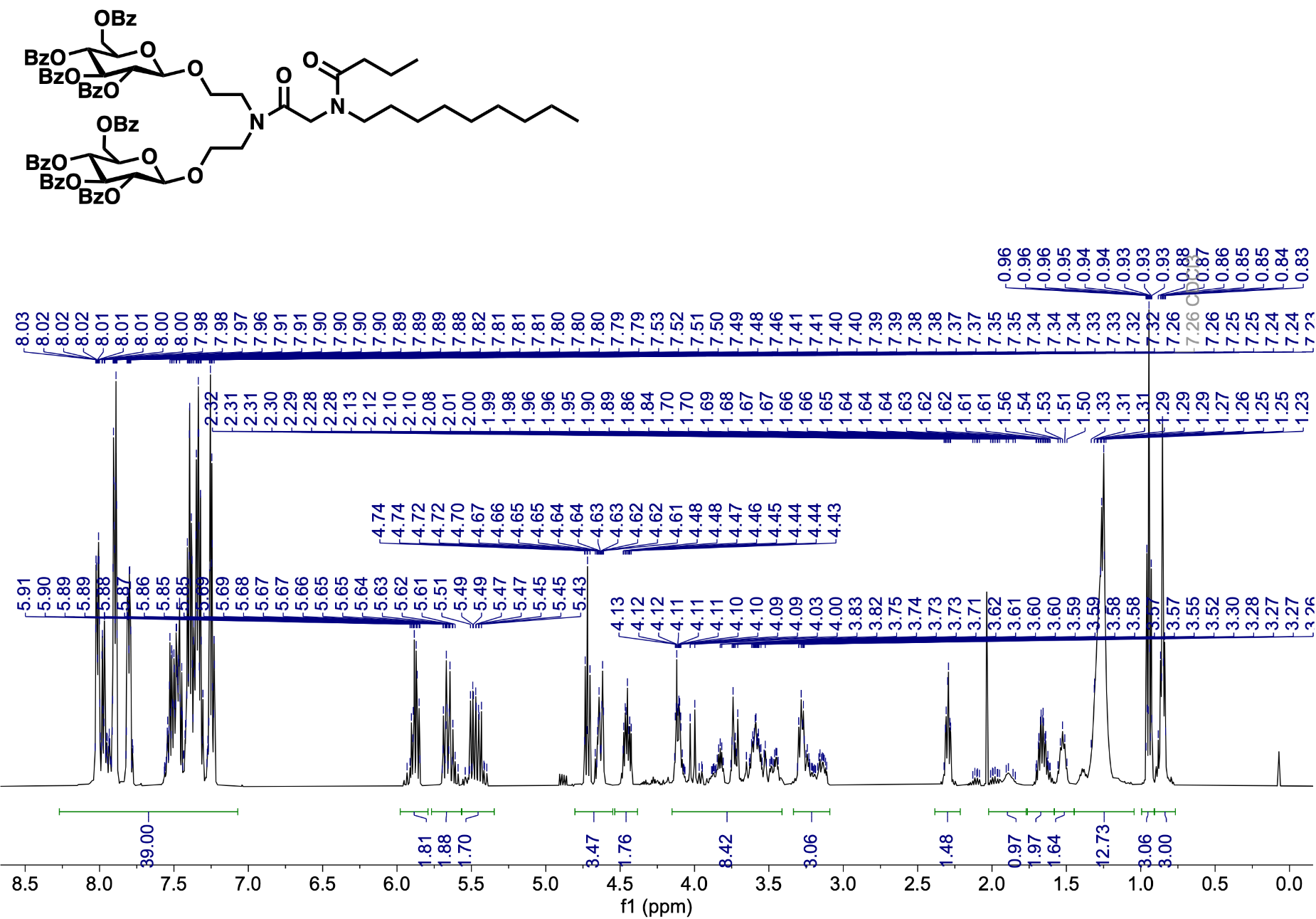
**

**Figure S35.** Enlarged view of a selected region of the ^1^H NMR spectrum of **21a** in CDCl_3_ at 500 MHz.


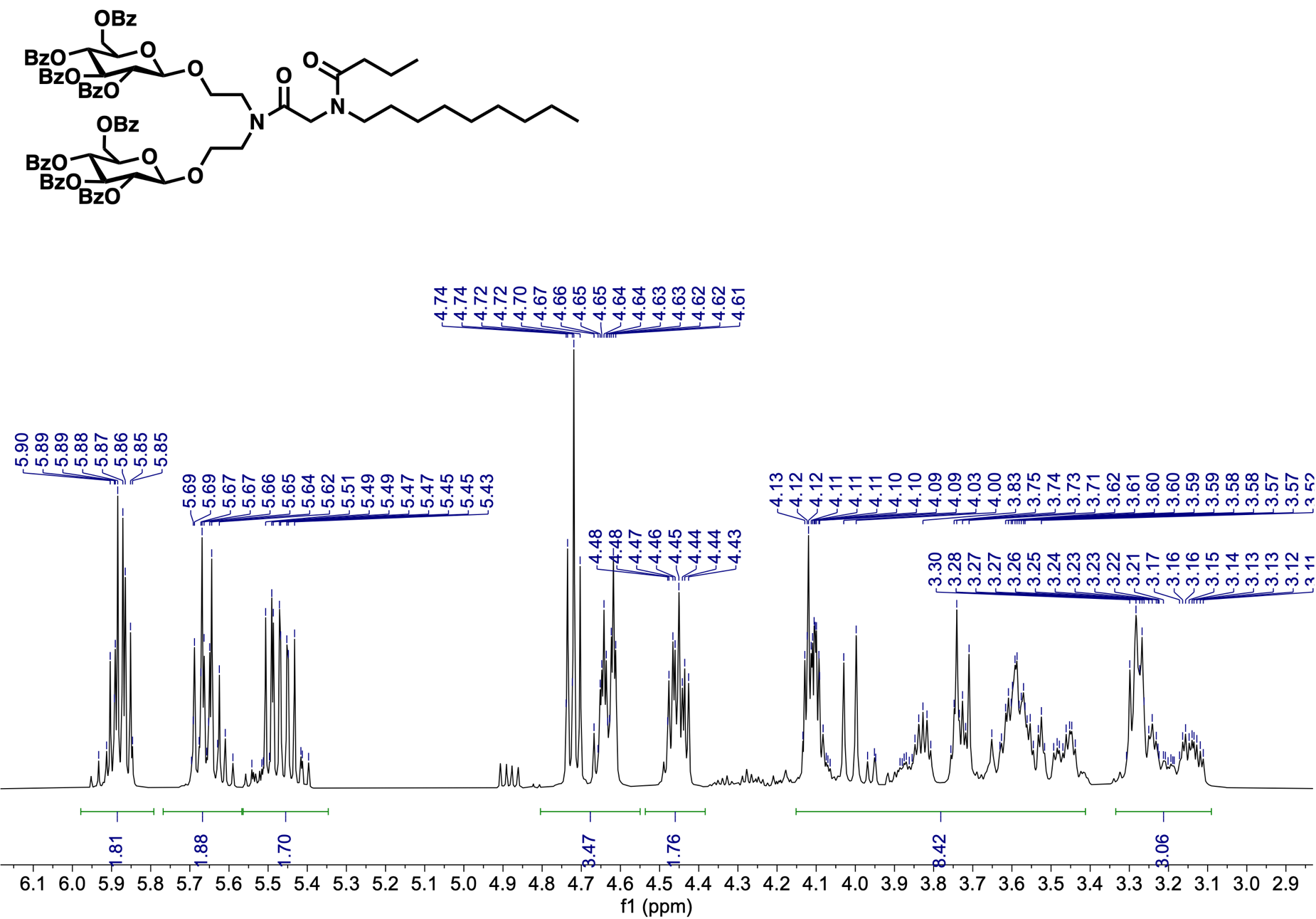


**Figure S36.** ^13^C NMR spectrum of **21a** in CDCl_3_ at 125 MHz

**
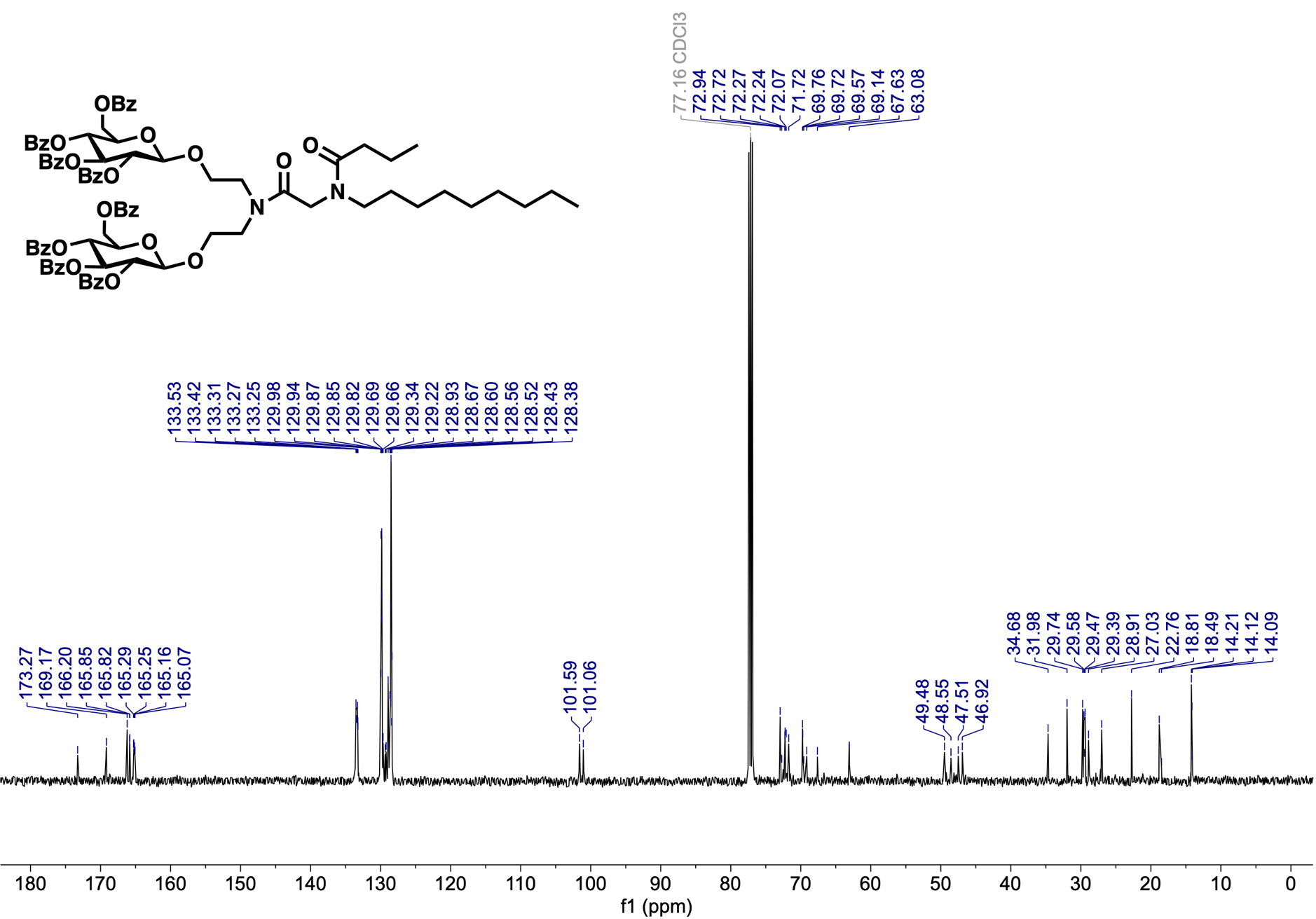
**

**Figure S37.** ^1^H NMR spectrum of **21b** in CDCl_3_ at 500 MHz

**
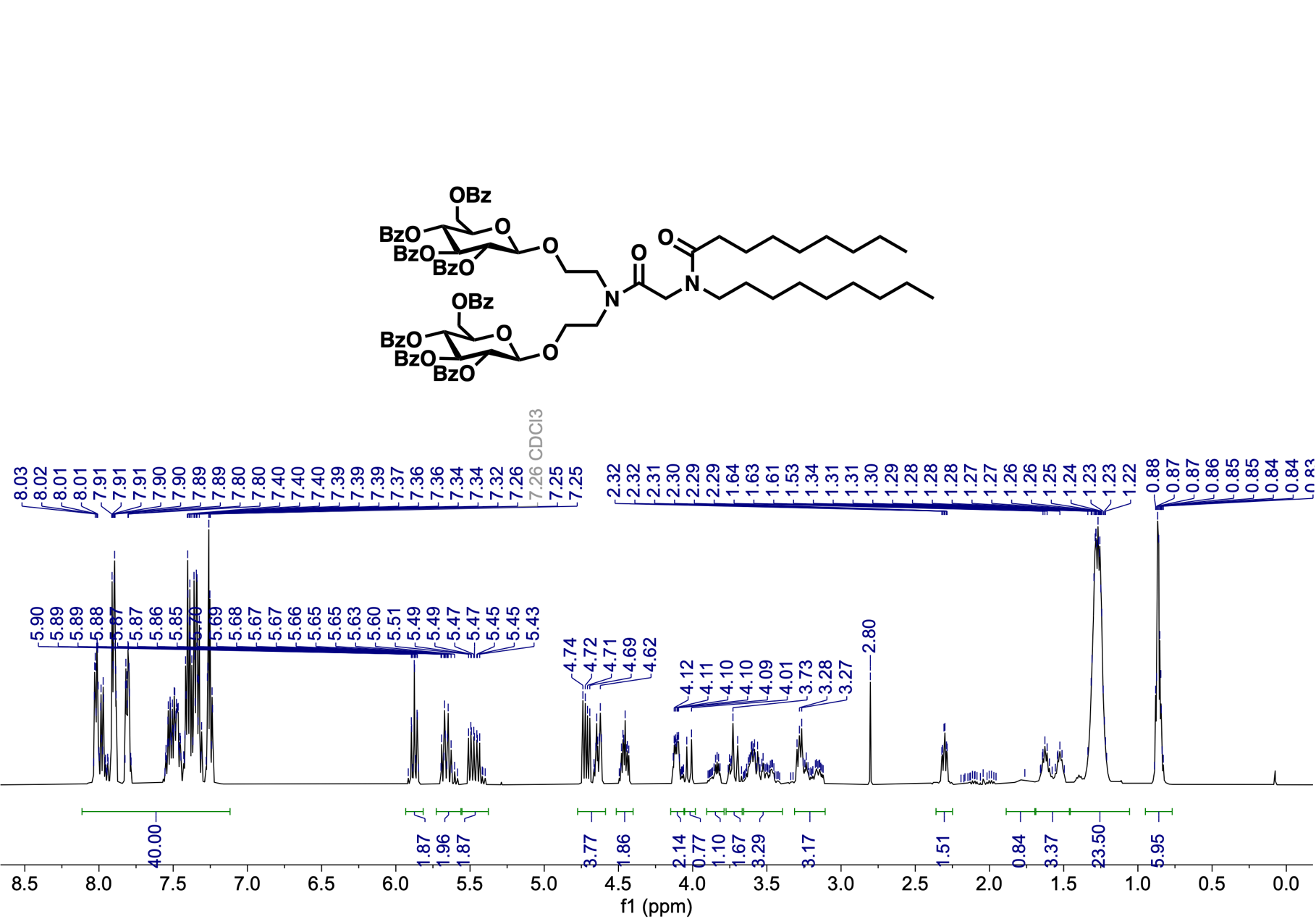
**

**Figure S38.** Enlarged view of a selected region of the ^1^H NMR spectrum of **21b** in CDCl_3_ at 500 MHz

**
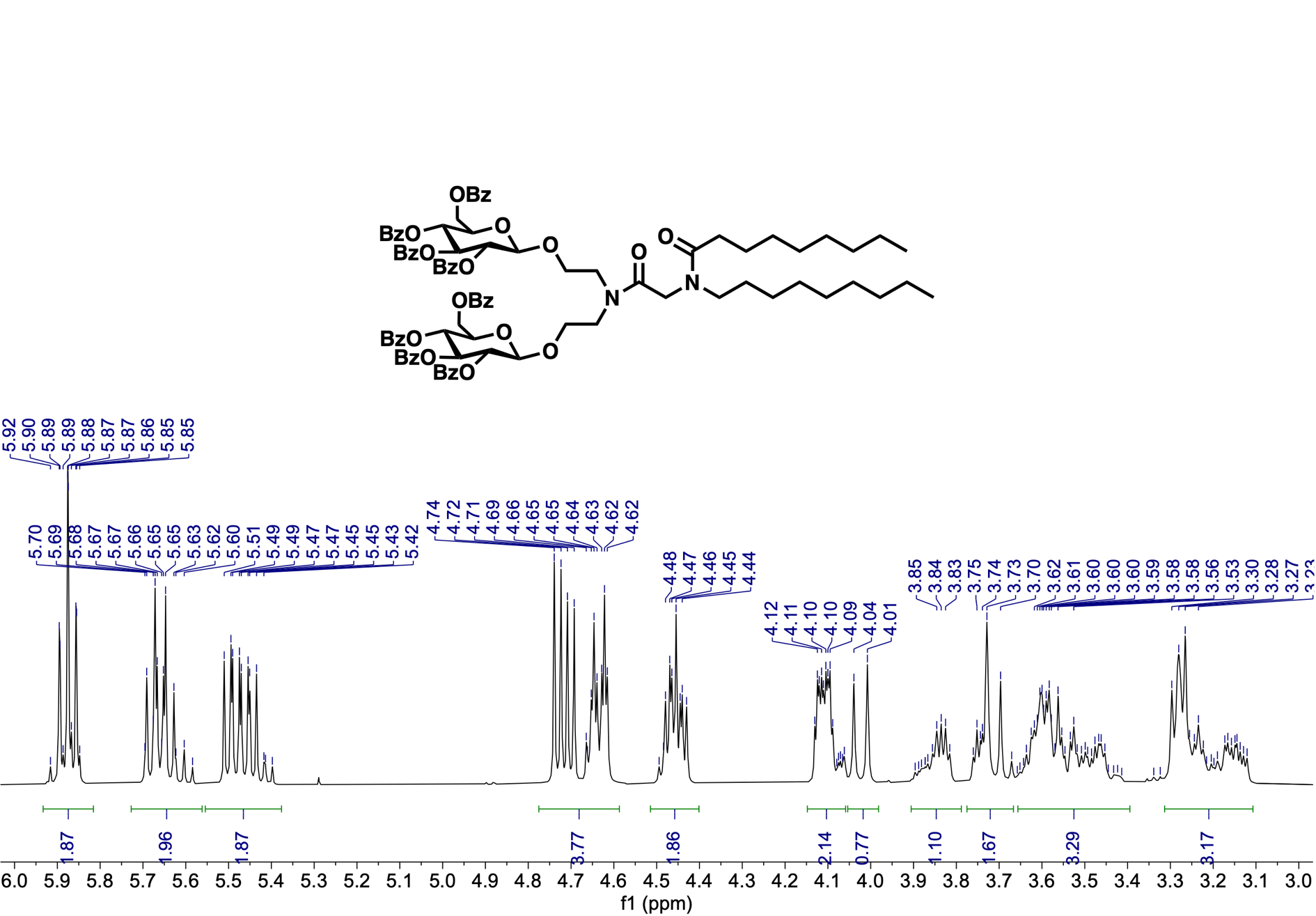
**

**Figure S39.** ^13^C NMR spectrum of **21b** in CDCl_3_ at 125 MHz

**
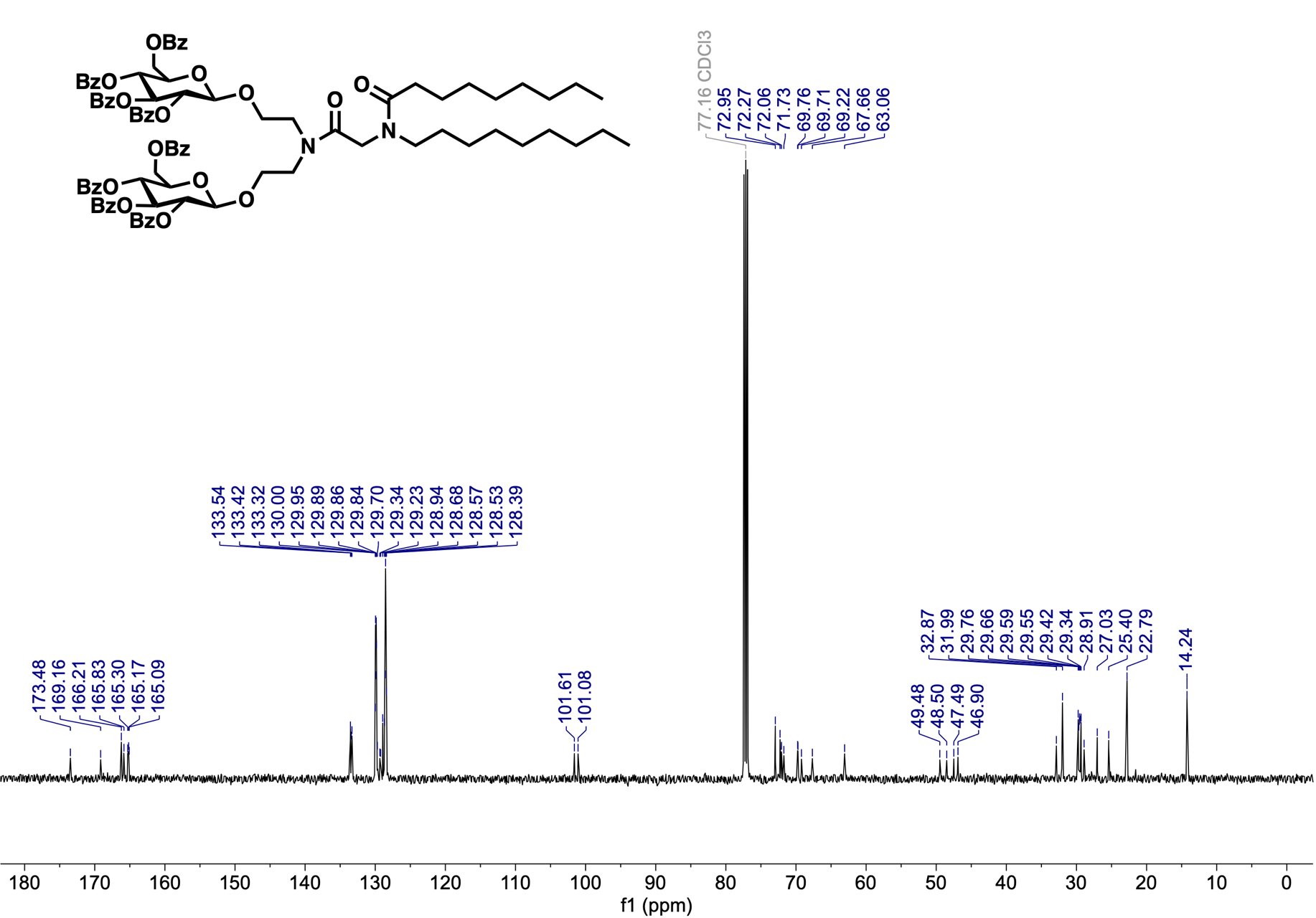
**

**Figure S40.** ^1^H NMR spectrum of **22a** in CD_3_OD at 400 MHz

**
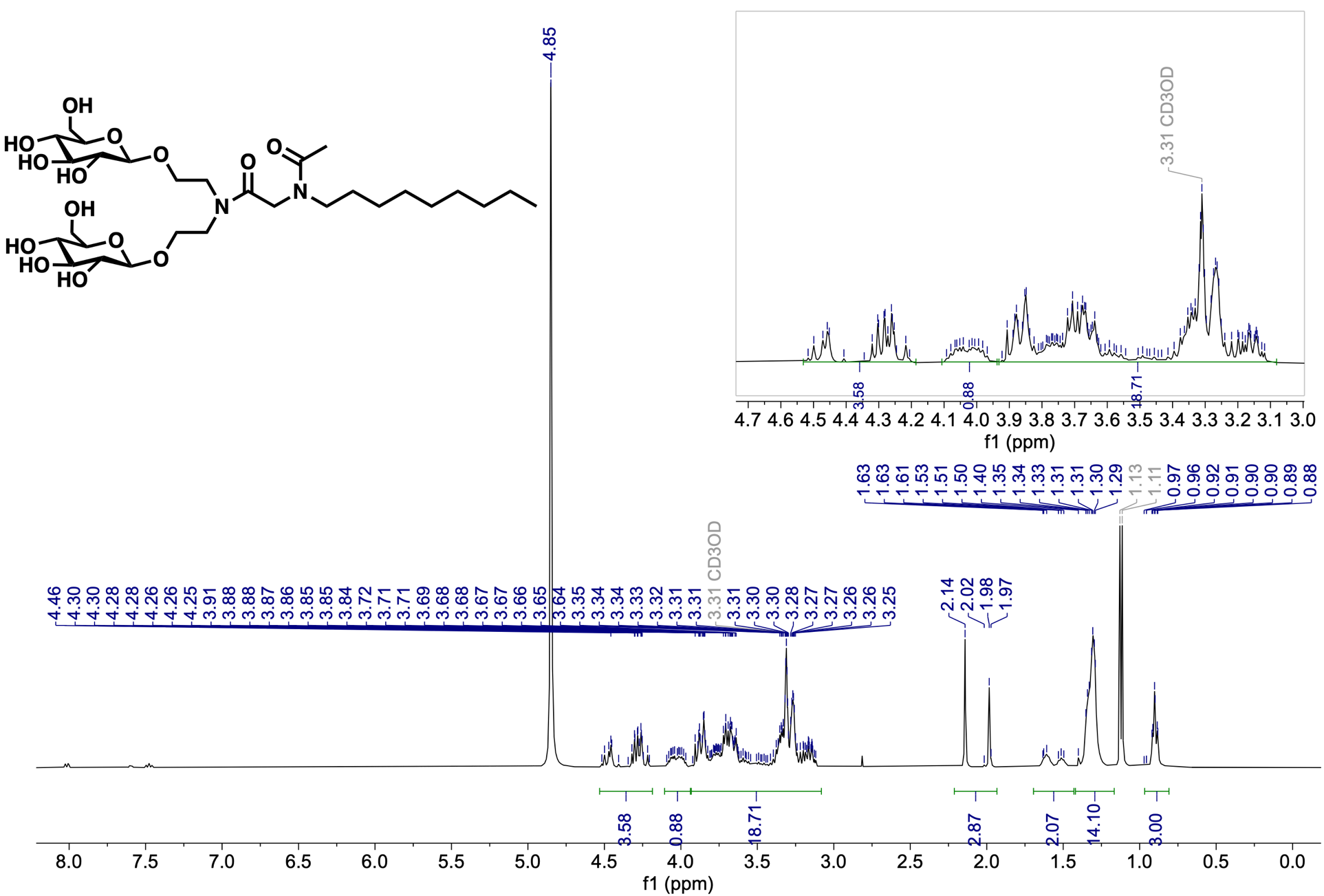
**

**Figure S41.** ^13^C NMR spectrum of **22a** in CD_3_OD at 125 MHz

**
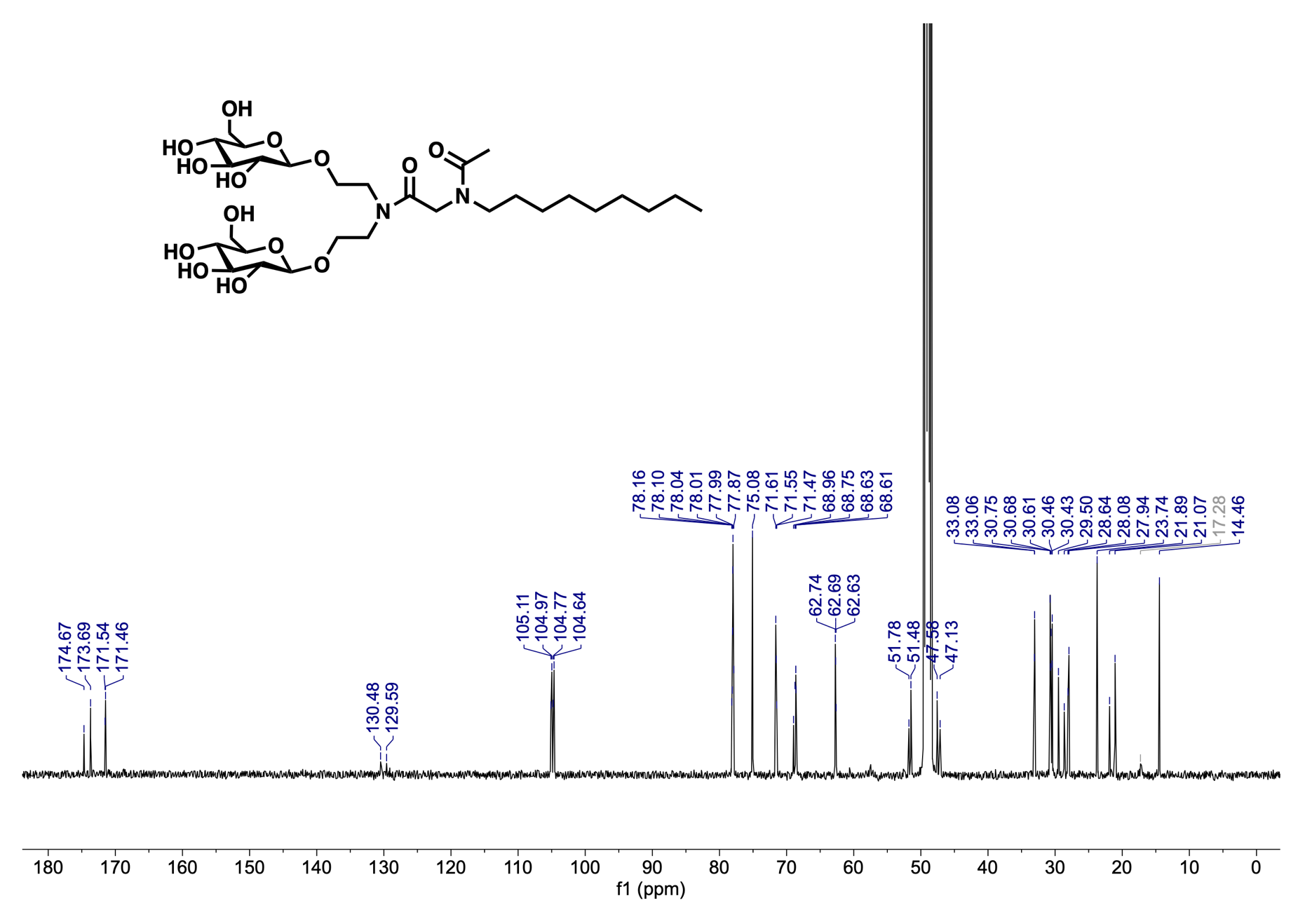
**

**Figure S42.** ^1^H NMR spectrum of **22b** in CD_3_OD at 400 MHz

**
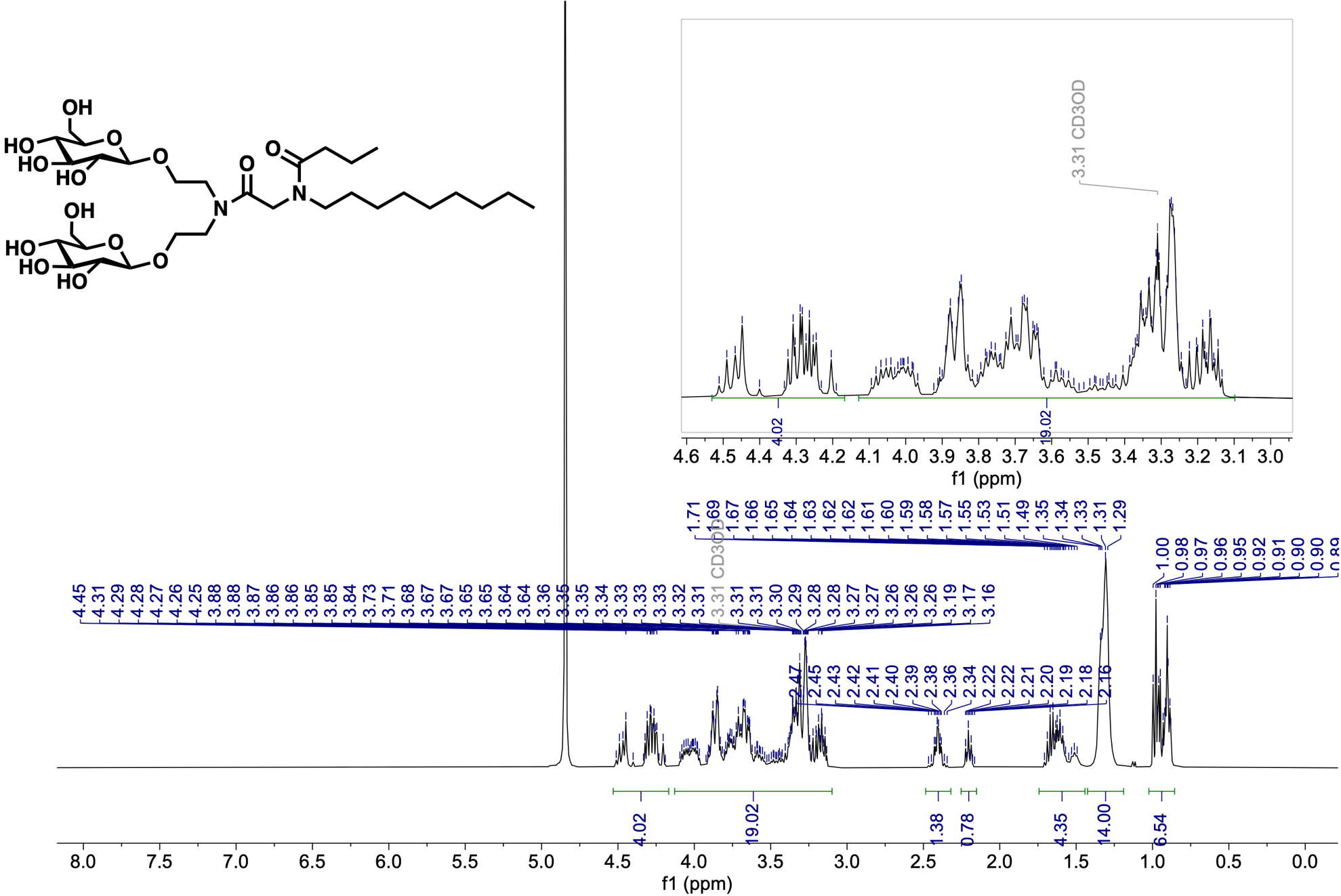
**

**Figure S43.** ^13^C NMR spectrum of **22b** in CD_3_OD at 125 MHz

**
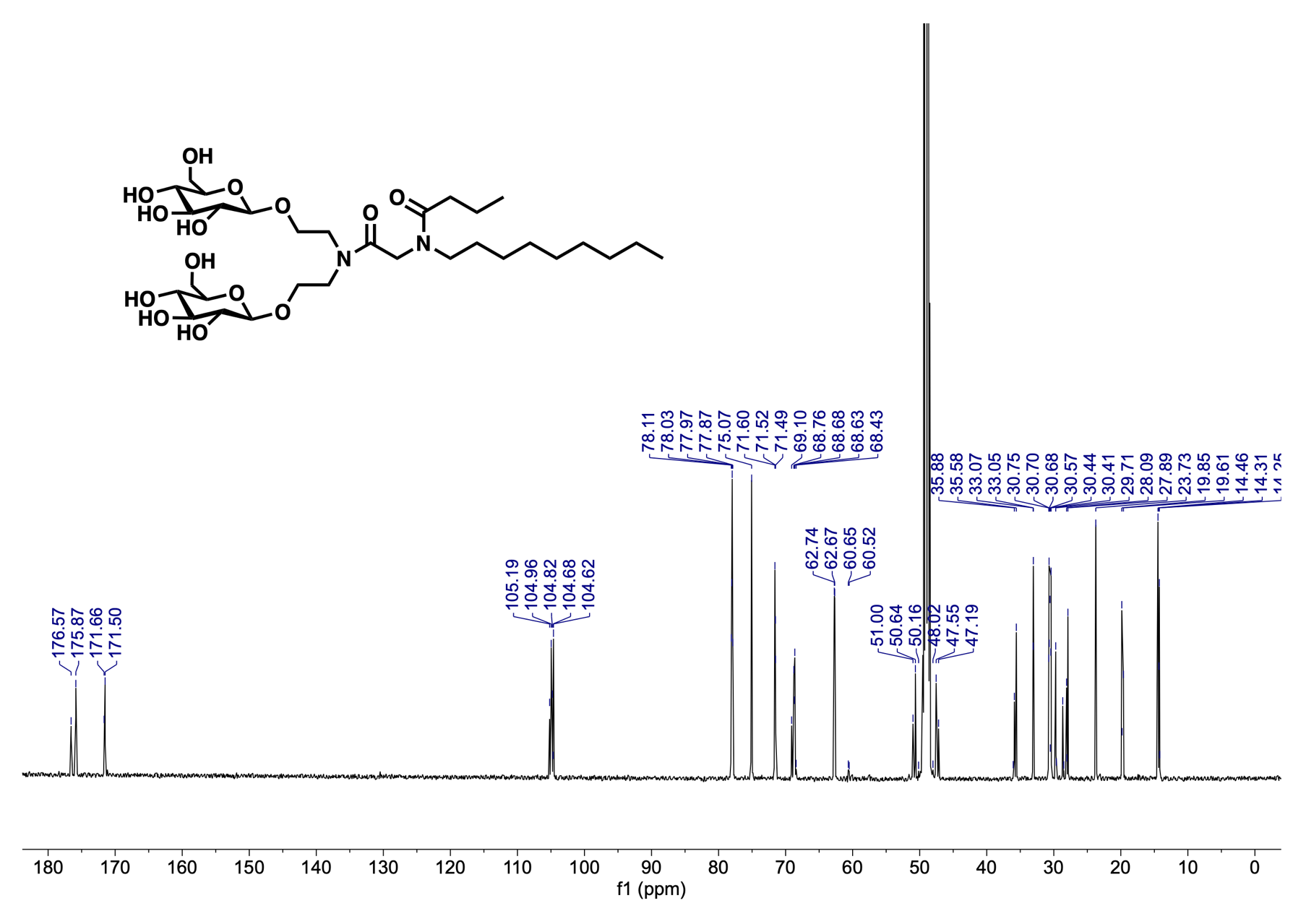
**

**Figure S44.** ^1^H NMR spectrum of **22c** in CD_3_OD at 400 MHz

**
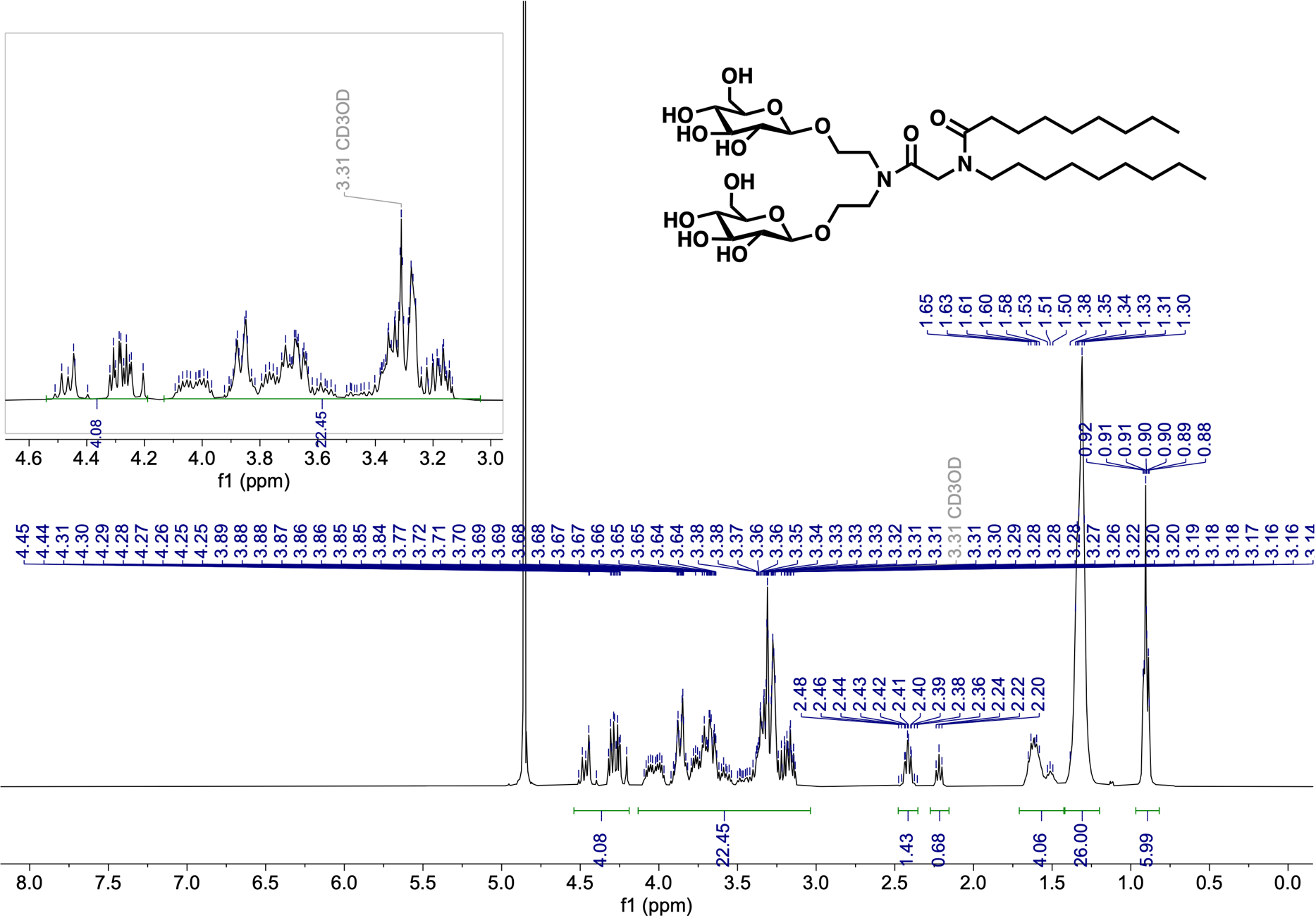
**

**Figure S45.** ^13^C NMR spectrum of **22c** in CD_3_OD at 125 MHz

**
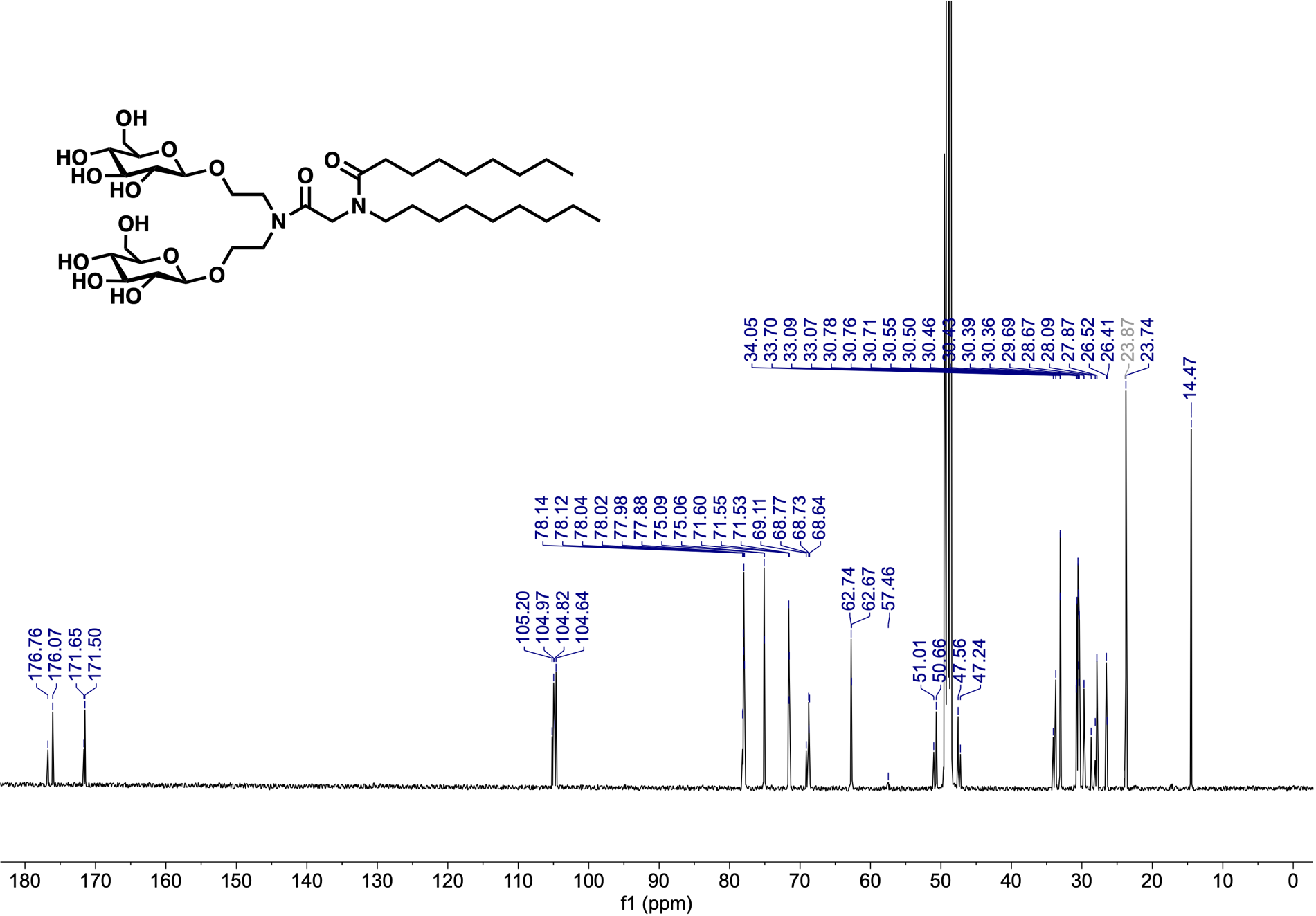
**

**Figure S46.** ^13^C NMR spectrum of **22c** in D_2_O at 100 MHz

**
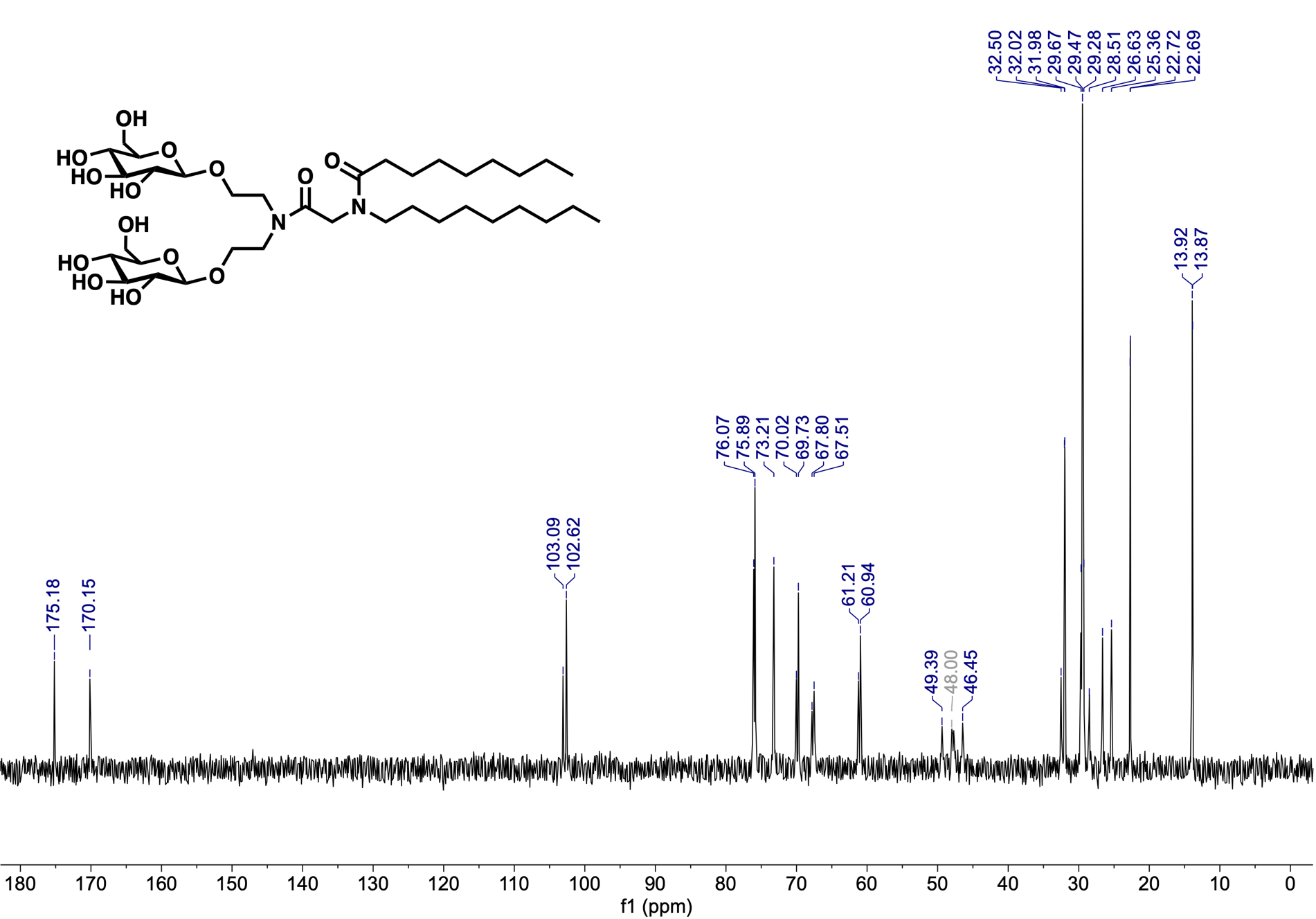
**

# References

(1) Grote Gansey, M. H. B.; Steemers, F. J.; Verboom, W.; Reinhoudt, D. N. *Synthesis* **1997**, *1997*, 643–648. doi:10.1055/s-1997-4506

(2) Schmidt, R. R. *Angew. Chem. Int. Ed. Engl.* **1986**, *25*, 212–235. doi:10.1002/anie.198602121

(3) Zhu, Z.-Y.; Yao, Q.; Liu, Y.; Si, C.-L.; Chen, J.; Liu, N.; Lian, H.-Y.; Ding, L.-N.; Zhang, Y.-M. *Journal of Asian Natural Products Research* **2012**, *14*, 429–435. doi:10.1080/10286020.2012.670220

(4) Yar, M.; Mushtaq, N.; Afzal, S.; Khan, A. S.; Khan, I. U.; Akhter, M. N.; Zareen, S.; Shahzad, S. A.; Khan, Z. A.; Raza Naqvi, S. A.; Mahmood, N.; Tahir, L.; Saleem, M. *Asian J. Chem.* **2013**, *25*, 7297–7304. doi:10.14233/ajchem.2013.14543
